# Dynamont: A comprehensive cross-species comparison of ONT segmentation tools

**DOI:** 10.1093/gigascience/giag005

**Published:** 2026-01-19

**Authors:** Jannes Spangenberg, Christian Höner zu Siederdissen, Winfried Goettsch, Lennart Köhler, Liz Maria Luke, Kai Papenfort, Manja Marz

**Affiliations:** RNA Bioinformatics and High-Throughput Analysis, Friedrich Schiller University Jena, 07743 Jena, Germany; RNA Bioinformatics and High-Throughput Analysis, Friedrich Schiller University Jena, 07743 Jena, Germany; RNA Bioinformatics and High-Throughput Analysis, Friedrich Schiller University Jena, 07743 Jena, Germany; RNA Bioinformatics and High-Throughput Analysis, Friedrich Schiller University Jena, 07743 Jena, Germany; Institute of Microbiology, Friedrich Schiller University Jena, 07743 Jena, Germany; Institute of Microbiology, Friedrich Schiller University Jena, 07743 Jena, Germany; Microverse Cluster, Friedrich Schiller University Jena, 07745 Jena, Germany; RNA Bioinformatics and High-Throughput Analysis, Friedrich Schiller University Jena, 07743 Jena, Germany; European Virus Bioinformatics Center, Friedrich Schiller University, 07743 Jena, Germany; FLI Leibniz Institute for Age Research, 07745 Jena, Germany

**Keywords:** Oxford Nanopore Technologies (ONT), signal segmentation, direct RNA sequencing (DRS), basecalling, hidden Markov model (HMM), dynamic time warping (DTW), event detection, *k*-mer models, benchmarking, RNA modifications

## Abstract

**Background:**

Oxford Nanopore Technologies (ONT) sequencing enables direct, long-read sequencing of DNA and RNA, preserving nucleotide modifications. During basecalling, deep neural networks translate raw nanopore signals into nucleotide sequences, internally segmenting the signal to align it with the corresponding bases. This is a challenging task due to uneven motor protein rotation, signal variability, low-quality reads, and the presence of nucleotide modifications. However, the signal to nucleotide assignment is critical for novel downstream signal analysis. Existing tools, such as Tombo Resquiggle, f5c Eventalign, f5c Resquiggle, and Uncalled4, operate after basecalling and rely on event-based segmentation and mapping approaches that often fail to align low-quality or modified reads and lack confidence estimates for segmentation accuracy.

**Results:**

Here, we present a large-scale comparative study in which 5 segmentation tools, including our novel tool Dynamont, are applied to 16 ONT-sequenced datasets spanning different kingdoms of life. Overall, we segmented 160,000 reads and evaluated the tools’ performance on a combination of 12 signal and downstream assembly metrics. Our study is accompanied by a comprehensive and extensible supplement that summarizes all datasets, execution instructions, and evaluation results. We score the segmentation results using an aggregated metric score, created from all our analyzed metrics.

**Conclusions:**

No tool delivered the best results for all datasets. We recommend a careful choice and normalization of evaluation metrics to select the best segmentation tool as a critical step in the process of ONT signal segmentation. Across nearly all RNA datasets, Dynamont outperforms other segmentation tools in terms of aggregated metric scores. For DNA datasets, however, the performance is more variable, with mixed results observed across tools.

Key Points

- Benchmark: 5 segmentation tools (+Dorado) across 16 ONT datasets with 12 metrics; fully reproducible.
- Dynamont: full forward-backward hidden Markov models, no mapping/events, posterior probabilities; best on most RNA datasets, segments short/low-quality reads.
- No universal winner; choose via carefully normalized, multimetric evaluation.
- Mapping/event tools (f5c, Uncalled4) often truncate/filter reads; Dorado moves are too coarse for fine signal-to-base alignment.

## Background

**Figure 1:**
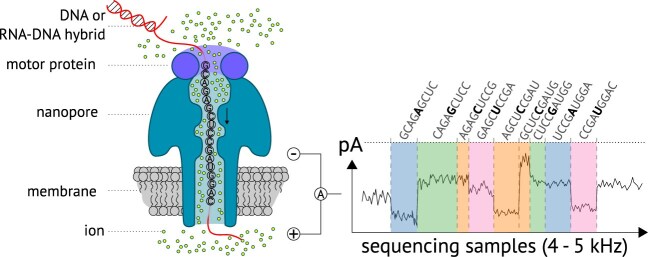
Nucleotide sequences pass the ONT nanopore directed by the motor protein upon an electrical field applied to the membrane. Nine nucleotides (or 5 for the old flow cell) are present in the pore at a time, while the ion flow is measured by a sensor in pico ampere (pA), leading to a characteristic sequencing signal. The time a 9-mer is present in the nanopre is unregular, and the signal of different nucleotides can be similar.

Oxford Nanopore Technologies (ONT) sequencing has gained widespread adoption due to several advantages: it enables cost-effective and portable sequencing, produces ultra-long reads, enables direct RNA sequencing, retains native nucleotide modifications, and allows for live basecalling during experiments. Oxford nanopore sequencing captures changes in electrical current over time as nucleotide sequences pass through a biological nanopore embedded in a membrane (Fig. 1). A sensor continuously measures these current fluctuations, which are characteristic of the nucleotide context within the pore. Depending on the sequencing settings, the data are sampled at 3–5 kHz, depending on the flow cell. In the case of DNA and the R10.4.1 flow cell sequencing, approximately 400 bases per second pass through the pore (for RNA, around 130 bases per second). For the older R9.4.1 flow cells, the translocation speeds were roughly 450 bases per second for DNA and 70 bases per second for RNA sequencing. The raw data, also called ONT signal, are stored in ONTs pod5 file format. The translocation speed it not constant and can variate. A typical analysis pipeline begins with basecalling the raw pod5 data using basecallers like Dorado. This is followed by various downstream tools for quality filtering, correction, and the detection of nucleotide modifications [1–3].

Despite its strengths, ONT sequencing is challenged by relatively high error rates [4]. These errors can arise from multiple sources: i) modified nucleotides that deviate from canonical signal models. Currently, around 170 different RNA modifications are known in the modomics database [5]. ONT provides basecalling models for N$^6$-methyladenosine (m$^6$A), pseudouridine ($\Psi$), inosine (Ino), 5-methylcytidine (m$^5$C), 2′-O-methyladenosine (mA), 2′-O-methylcytidine (mC), 2′-O-methylguanosine (mG), and 2′-O-methyluridine (mU). ONT keeps advancing in the field of RNA modification calling. Furthermore, training basecalling models with modifications showed an improvement in basecalling accuracy [6]. ii) Another error source is the uneven translocation speed of molecules through the nanopore due to the motor protein dynamics. This causes nucleotide signals to differ in length (Fig. 1). In the worst case, a nucleotide sequence can get stuck in the pore, stopping the sequencing of that read. iii) Also, homopolymer regions are a fundamental problem in ONT sequencing [7]. In the new R10 pores, 9 nucleotides are measured at once. A homopolymer stretch above 9 nucleotides is hard to predict, as it will produce a homogeneous sequencing signal. iv) Finally, and of major importance for this work, are inaccuracies in the segmentation of the raw signal. Dorado can provide a low-resolution signal segmentation when using the –emit-moves parameter.

Dorado’s main task is basecalling. Basecalling and signal segmentation represent distinct stages in ONT data interpretation. Signal segmentation divides the continuous current signal into regions corresponding to specific *k*-mers, providing an explicit mapping between signal and sequence. In contrast, basecalling directly translates the ONT signal into nucleotide sequences using neural networks, without such an intermediate alignment. Modern basecallers like Dorado perform end-to-end inference, learning the signal-to-sequence relationship implicitly rather than through predefined segmentation steps. Segmentation tools, however, typically require basecalled reads to align the ONT signal to the corresponding sequence, enabling fine-grained analyses. This explicit mapping remains essential for applications such as RNA modification detection and signal-level benchmarking, where positional accuracy and interpretability are critical.

Segmentation of the raw ONT signal is particularly critical when analyzing the raw signal directly (e.g., the signals of nucleotide modifications), for which no prediction tool exists yet. Improper segmentation can propagate errors into downstream analyses. However, current segmentation tools are still underdeveloped, and their performance has not been systematically benchmarked across diverse datasets and conditions.

In this work, we benchmark 5 segmentation tools: Tombo Resquiggle (by ONT), f5c Eventalign  [8], f5c Resquiggle  [8], Uncalled4  [9], and our proposed method Dynamont. While they all aim to align raw ONT signals to nucleotide sequences, they vary substantially in algorithm and requirements. Except for Dynamont, all tools rely on signal preprocessing via event detection, typically using a sliding window *t*-test originally implemented in Scrappie, an early ONT basecaller.

**Figure 2:**
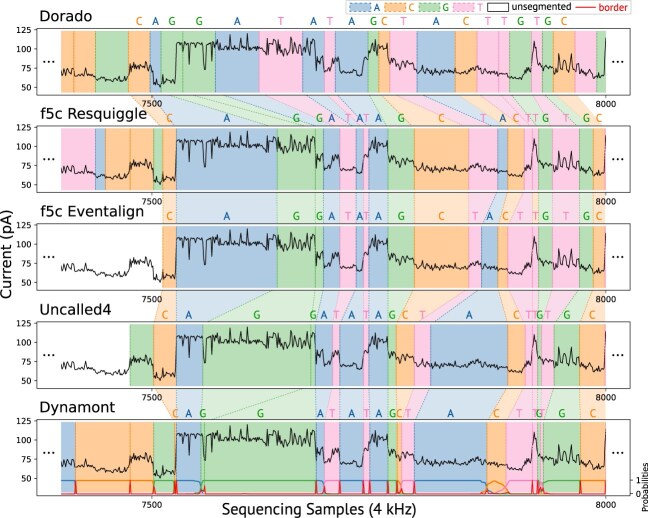
Immense differences in various tool segmentations in an RNA004 *H. sapiens* read fragment. The central base of the aligned *k* = 9-mer is colored. For segmentation improvement, the tool Dorado is input for all other tools. f5c Eventalign and Uncalled4 do not segment the read completely and neglect read information. Tombo Resquiggle is not displayed in the figure, as it is not able to segment RNA004 reads. f5c Eventalign and Uncalled4 are not segmenting the complete read due to unmapped parts of the read, as seen in the unsegmented signal part on the left. Dynamont is the only tool that returns additional segmentation probabilities (i.e., the border probability [red] or nucleotide probabilities).

Tombo Resquiggle requires prior mapping of reads to a reference sequence. It applies Scrappie’s event detection as a preprocessing step, followed by a forward-only hidden Markov model (HMM) pass. Segmentation is performed using *k*-mer–based signal distribution models that associate raw nanopore signal segments with expected current levels. f5c Eventalign is an optimized reimplementation of nanopolish eventalign. It also requires read mapping to a reference sequence and uses Scrappie’s event detection for signal preprocessing. Like Tombo Resquiggle, it applies an HMM forward pass and segments the nanopore signal using *k*-mer–based signal models. f5c Resquiggle is closely related to f5c Eventalign but operates without requiring a mapping to a reference genome. Instead, it uses the basecalled reads directly for segmentation. It applies Scrappie’s event detection and an HMM forward pass to align the detected events to the read sequence. Uncalled4 also requires read-to-reference mapping and implements Scrappie’s event detection. However, instead of using an HMM, it employs a dynamic time warping (DTW) algorithm to align the signal to the reference sequence, using an adaptive banded dynamic programming approach. Dynamont does not require any mapping or event detection. It directly uses the basecalled reads to align to the raw nanopore signal by performing both the forward and backward passes of an HMM. This full forward–backward algorithm enables the calculation of posterior probabilities for each segment, allowing for more refined segmentation and confidence estimates (Fig. 2).

### Tool comparison

**Figure 3:**
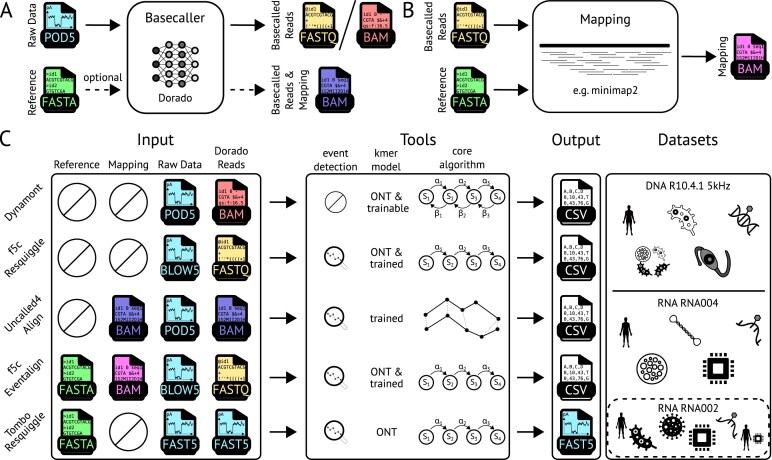
Overview of segmentation tools, their input, basic methods, and applications. (A) Dorado is input for improvement in all compared segmentation tools. Dorado translates ONT raw data (*.pod5, turquoise) into the basecalled reads. Depending on parameters and the input, reads can be stored in *.fastq (yellow), basecalled *.bam (red), or basecalled and mapped *.bam (blue) when Dorado is provided with a reference sequence (green). Internally, Dorado utilizes minimap2 to directly map the basecalled reads. (B) After basecalling, reads (*fastq, yellow) can be optionally mapped to a reference sequence (*.fasta, green) using minimap2, resulting in yet another bam file (*.bam, purple). (C) Segmentation tools require different input files and formats. All of them need the raw data and the basecalled reads (again in different file formats). The output is provided in individual formats, often in tabular *.csv format. Except for Tombo Resquiggle, every tool can process datasets of different chemistries and nanopores. Tombo Resquiggle is deprecated and cannot work with newer R10 data. We executed the tools on 16 datasets from different origins and 3 different chemistry/pore types. All tools except Dynamont use ONT’s event detection algorithm for presegmentation. Dynamont utilizes the full forward–backward algorithm, while other tools only implement the forward pass of HMM or DTW. The internal *k*-mer models used by each tool differ in their design and training approach. Tombo Resquiggle and Dynamont rely on the default ONT *k*-mer distribution models, although Dynamont additionally offers the option to train a custom model. Both f5c modules use ONT-provided models for the R9 pore as well as custom-trained models for newer pore versions. In contrast, Uncalled4 exclusively employs trained models.

These methodological differences lead to significant divergence in where in (a) the raw read signal the segmentation is assigned to (Fig. 2), (b) the number of reads segmented or truncated (due to filtered reads—unmappable or low quality) (Table 2), (c) what data are required as input (Fig. 3) and, especially, (d) handling data from different organisms and sources (Fig. 7). Full technical details for each tool are provided in the Methods.

Kovaka et al. [9] introduced a metric to measure the segmentation similarity between tools, the Jaccard similarity/distance. Unfortunately, this metric can only be used when a common reference sequence onto which the reads are mapped is available. For the mentioned tools, which perform segmentation independent of a mapping to a reference sequence, a comparison has not been possible so far.

Here, we compare in a large-scale comparative study Dorado and the 5 segmentation tools Uncalled4, f5c Eventalign, f5c Resquiggle, Tombo Resquiggle, and our own tool Dynamont. We applied them to 12 ONT-seqeunced DNA and RNA datasets spanning RNA002, RNA004, and DNA (R10.4.1 5 kHz) sequencing protocols from different types of flow cells. We evaluated the tools by a combination of 13 metrics. Furthermore, we will evaluate the usability of the tools, as well as the memory and runtime usage.

## Materials: Data Description

We included 16 datasets across 7 different sources (Table 1). The datasets cover mammals (*Homo sapiens*, fungi *Saccharomyces cerevisiae* and *Podospora anserina*), bacteria (*Escherichia coli* and *Staphylococcus aureus*), a virus (SARS-CoV-2), a plant viroid (citrus exocortis viroid [CEVd]), a synthetic metagenome dataset (Zymo HMW, containing DNA of fungi and bacteria), and synthetic data (*in vitro* transcribed [IVT]). We basecalled all datasets using the Dorado basecaller (v0.9.1). For each dataset, we extracted 10,000 randomly selected reads. Each tool was given the same 10,000 randomly selected reads per dataset. After basecalling, the number of reads can differ slightly from 10,000, as Dorado can split 1 read signal into 2 basecalled reads (Supplementary Fig. S2). The RNA002 chemistry and R9.4.1 flow cells are no longer provided by ONT. However, numerous datasets generated using this configuration remain publicly available in sequencing repositories. ONT has since transitioned to the newer R10.4.1 flow cell series.

**Table 1:**
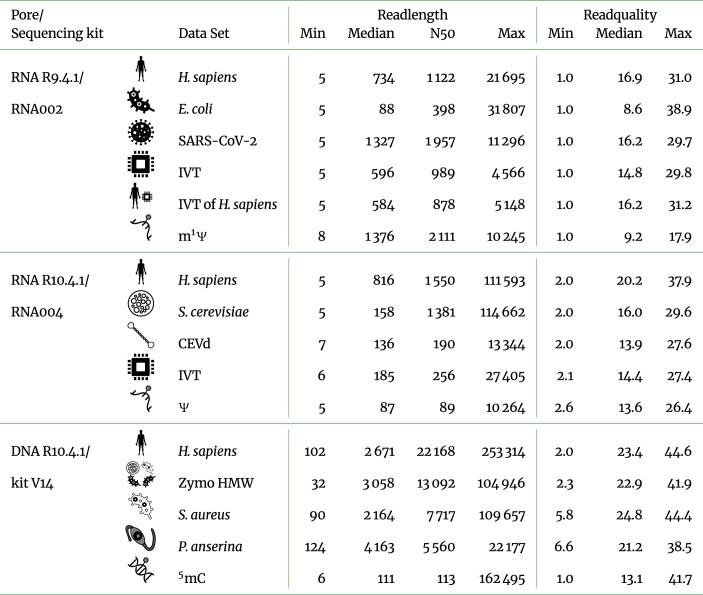
Dataset overview of read length statistics for 10,000 randomly selected reads from each of the 16 datasets. The R9.4.1 flow cells operate on $k=5$-mers, the R10.4.1 ones on $k=9$-mers. IVT: *in vitro* transcription (synthetic); CEVd: citrus exocortis viroid; Zymo high molecular weight (HMW): synthetic metagenome dataset; N$^1$-methylpseudouridine (m$^1\Psi$): dataset containing modifications; pseudouridine ($\Psi$): dataset containing modifications; 5-methylcytosine ($^5$mC): dataset containing modifications. The read length and quality are taken from the the basecalled reads after basecalling with Dorado.

### RNA002 datasets

The following 4 datasets were directly RNA sequenced using ONT’s R9.4.1 MinION FLO-MIN106 flow cells with the RNA002 protocol. We basecalled them using the rna002_70bps_hac@v3 model (see supplemental material).

#### The *H. sapiens* dataset

The dataset was downloaded from the ENA using the project ID PRJEB40872 with accession ID ERR4706156 (wild-type replicate 1) [10]. This dataset contains human transcriptomic RNA (total RNA) reads of the HEK293T cell line. As a reference sequence, we used the complementary DNA (cDNA) GRCh38 reference by the ensembl (release 113) for all required mapping processes.

#### The *E. coli* K12 dataset

This strain was grown aerobically in LB medium at 37$^{\circ }$C to OD600 of 1.0. Cells were harvested by addition of 0.2 volumes of stop mix (95 % ethanol, 5 % (*v/v*) phenol) and snap frozen in liquid nitrogen. Total RNA was isolated using the SV total RNA purification kit (Promega; AM1907), digested with TURBO DNase (Thermo Fisher Scientific), and ribosomal RNA (rRNA) was removed by the Ribo-Zero rRNA Removal Kit (Illumina; MRZGN126) according to the manufacturer’s instructions. The amount of RNA was measured by the Qubit RNA-HS (High Sensitivity) Assay Kit (ThermoFisher Scientific; Q32852) according to the manufacturer’s instructions. Finally, a poly(A) tail was added to the RNAs by incubation of 400 ng RNA with 300 nM ATP and 2.5 U *E. coli* Poly(A) Polymerase (NEB; M0276) for 30 minutes at 37$^{\circ }$C. The poly(A) tailed RNA products were again purified using the RNAClean XP beads 1:1 volume RNA reaction mix to bead volume (Beckman Coulter; A66514) following the manufacturer’s instructions. The cleaned RNA was measured by the Qubit RNA-HS (High Sensitivity) Assay Kit. RNA sequencing was performed following the protocol provided by ONT, using R9.4.1 chemistry flow cells (FLO-MIN106) and the direct-RNA chemistry sequencing kit (SQK-RNA002). For library preparation, we used 50 ng poly(A) tailed RNA, prepared as described above, using the provided polyT (RTA) adapter. For all the mapping processes, we used the *E. coli* K12 cDNA reference from ensembl release 60 GCA_000005845 ASM584v2.

#### The *SARS-CoV-2* dataset

This virus is the alpha variant, and it is available from the SRA under the project PRJNA907180 using the run accession SRR22476725. The dataset contains RNA reads derived from Vero cells of African green monkeys infected with virus isolates obtained from human nasopharyngeal swabs [11]. For reference, the cDNA reference by Ensembl ASM985889v3 was used.

#### The synthetic IVT dataset

The *in vitro* transcribed data contain unmodified RNA, created by a synthetic double-stranded DNA template (1,297 bp). DNA was ordered (gBlocks GeneFragments; Integrated DNA Technologies) containing the sequences of different 5-mers. Then, 200 ng double-stranded DNA template was used in 20-$\mu$L IVT reactions for 1 hour using the HighScribe T7 RNA synthesis Kit (NEB–E2040S), following the manufacturer’s instructions. After IVT, the DNA templates were digested by addition of 20 $\mu$L RNase-free water, including 2 U DNase I (NEC–M0303S) for 10 minutes at 37$^{\circ }$C. DNase digestion was stopped by addition of 5 mM EDTA (final concentration) and heat inactivation for 10 minutes at 75$^{\circ }$C. The RNA products were purified using the RNAClean XP beads 1:1 volume IVT/DNase reaction mix to bead volume (Beckman Coulter; A66514) following the manufacturer’s instructions. The cleaned RNA was measured by the Qubit RNA-HS (High Sensitivity) Assay Kit (ThermoFisher Scientific; Q32852) according to the manufacturer’s instructions. Finally a poly(A) tail was added to the cleaned RNA by incubation of 3 $\mu$g RNA with 1 mM ATP and 5 U *E. coli* poly(A) Polymerase (NEB–M0276) for 30 minutes at 37$^{\circ }$C. The poly(A) tailed RNA products were again purified using the RNAClean XP beads 1:1 volume RNA reaction mix to bead volume (Beckman Coulter; A66514) following the manufacturer’s instructions. The cleaned RNA was measured by the Qubit RNA-HS (High Sensitivity) Assay Kit. RNA sequencing was performed following the instruction provided by ONT, using R9.4.1 chemistry flow cells (FLO-MIN106) and the direct RNA chemistry sequencing kit (SQK-RNA002). For library preparation, we used 1 $\mu$g of poly(A) tailed IVT RNA templates, prepared as described above, using the provided poly(T) (RTA) adapter.

#### The IVT dataset of *H. sapiens*

This dataset is an IVT experiment of the human transcriptome available via the SRA Project PRJNA947135 with the accession ID SRR23950400. The data are derived from the messenger RNA (mRNA) of an HeLa cell line [12]. Therefore, it contains human mRNA without any RNA modifications. We used the same reference sequence as in the *H. sapiens* dataset.

#### The m$^1\Psi$ dataset

This dataset contains synthetic RNAs known as “curlcakes” that contain m$^1\Psi$ RNA modifications instead of the canonical U [13]. The data are available via the ENA project PRJEB67632 and run accession ERR12146341. “Curlcakes” sequences are used as the reference.

### RNA004 datasets

All datasets were directly RNA sequenced with ONT’s MinION FLO-MIN004RA or FLO-PRO004RA flow cell using the RNA004 protocol. Dorado with the rna004_130bps_supv5.1.0 model was used to basecall the data (see supplemental material).

#### The *H. sapiens* dataset

This universal human reference RNA benchmark dataset is taken from the Garvan Institute Long Read Sequencing Benchmark Data. This dataset contains *in vivo* direct RNA sequencing (DRS) reads, sequenced on a PromethION (FLO-PRO004RA), and is available via the Amazon web service. For this dataset, the same reference was used as for RNA002.

#### The *S. cerevisiae* dataset

The fungi originate from the BY4741 parental strain [14]. The dataset contains *in vivo* RNA reads, sequenced on a FLO-MIN004RA using the RNA004 protocol, and is available via the SRA under the project PRJNA1150648 using the run accession SRR30335016. The *S. cerevisiae* R64-1-1 cDNA reference from the Ensembl (release 113) database was used.

#### The CEVd dataset

This dataset contains reads from small circular noncoding RNAs, which act as infectious pathogens in higher plants [15]. It was sequenced on the FLO-MIN004RA flow cell using the RNA004 protocol and is available via Zenodo (PV259 [16]). Here we used the CEVd reference sequence found in the ENA under the accession AJ490825.1. As the CEVd is a circular RNA, it introduces mapping complications if a read spans the end and start of the reference sequence. To counter this, the reference sequence was once concatenated with itself.

#### The synthetic IVT dataset

This was synthetized CEVd RNA, free of any modifications [15]. The dataset was also sequenced on the FLO-MIN004RA flow cell using the RNA004 protocol and is available via Zenodo (PV260 [16]). The same reference was used as for the CEVd dataset.

#### The $\Psi$ dataset

This dataset is from ONT directly (Open Data project—RNA mod validation data) and can be downloaded from ONT’s EPI2ME website. It contains RNAs with $\Psi$ modifications in random contexts. We used the $\Psi$ replicate 1 dataset. The used reference sequence is also available via the website.

### DNA R10.4.1 datasets

We used the dna_r10.4.1_e8.2_400bps_sup@v5.0.0 model to basecall the following datasets.

#### The *H. sapiens* dataset

The DNA R10.4.1 5-kHz dataset is obtained from the universal human reference benchmark dataset Garvan Institute Long Read Sequencing Benchmark Data with the ID: NA24385 (HG002). The dataset contains human DNA sequenced with the SQK-LSK114 kit on a PromethION flow cell with a coverage of ~40 %. As a reference, the GRCh38 toplevel dna Ensembl (release 112) was used.

#### The Zymo HMW dataset

This dataset is taken from the ENA database with the project ID PRJEB64570 containing DNA sequenced on a FLO-MIN114 flow cell using the SQK-LSK114 sequencing kit and a sampling rate of 5 kHz. This dataset contains DNA from a mix of organisms, representing a synthetic metagenomic dataset: *Pseudomonas aeruginosa* (14%), *E. coli* (14%), *Salmonella enterica* (14%), *Enterococcus faecalis* (14%), *S. aureus* (14%), *Listeria monocytogenes* (14%), *Bacillus subtilis* (14%), and *S. cerevisiae* (2%). We took the reference sequences from Sereika et al. [17].

#### The *S. aureus* dataset

This bacteria dataset is taken from the BioProject PRJNA1091452 with the run ID SRR31990262, which was published in Dabernig-Heinz et al. [18]. It is a Gram-positive, spherically shaped bacterium sequenced on a R10.4.1 flow cell with the v14 sequencing kit (SQK-LSK114). The reference sequence is taken from the assembly ASM2249454v1.

#### The *P. anserina* dataset

These ascomycete fungus genomic data from the CaDa-strain are taken from the BioProject PRJNA1216259 under the run ID SRR32173960 from Ament-Velásquez et al. [19]. The fungus was sequenced using a MIN-FLO114 flow cell with the SQK-NBD114.24 sequencing kit. We used the assembly provided in the study as a reference sequence.

#### The $^5$mC dataset

This dataset is taken from ONT’s Open Data project (mod validation data) available on the EPI2ME website. It contains the $^5$mC modification in all 5-mer contexts. We used the $^5$mC replicate 1. The used reference sequence is also available via the website.

## Methods: Segmentation Tools in Detail

### Dorado

Dorado is ONT’s state-of-the-art basecaller and was used to generate the input for all of the compared segmentation tools. Standard basecaller models are trained on canonical bases A, C, G, and T/U. Special models were additionally trained on specific base modifications, for example, for the DNA modifications N4-methylcytosine ($^4$mC), $^5$mC, 5-hydroxymethylcytosine ($^5$hmC), and N$^{6}$-methyladenine ($^6$mA) of 17 known modifications [20]. For the 170 RNA modifications, ONT provides models for m$^6$A, $\Psi$, m$^5$C, Ino, mA, mC, mG, and mU. During basecalling, Dorado provides an optional segmentation (–emit-moves parameter) with a low resolution. This resolution results from downsampling the input signal, within the neural network (Supplementary Fig. S1). The model’s focus is to basecall the data, not to assign signals to nucleotides, as is the case for segmentation tools (see Fig. 3).

### Tombo Resquiggle only works on R9 data

Tombo Resquiggle (v1.5.1) is a segmentation tool developed by ONT. Tombo Resquiggle is deprecated and does not support the signal file formats multi fast5 and pod5, nor does it support sequencing protocols for the new RNA or DNA chemistries (R10.4.1 pores). It utilizes an HMM forward pass to segment the signal and takes multiple inputs: the signal in the old single fast5 format, the basecalled reads that need to be added to the single fast5 files, and a reference sequence in fasta format. Tombo Resquiggle preprocesses the data first in 3 steps: *i) Mapping reads to a reference sequence:* The segmentation process begins by mapping reads to the reference sequence, to correct for sequencing errors. Unmapped nucleotides are not segmented by Tombo Resquiggle. *ii) Normalizing the signal:* Read signals are normalized using the signal median shift and median absolute deviation (MAD) scaling to standardize signal levels across reads. *iii) Performing an event detection on the signal:* The event detection is taken from Scrappie, an older ONT basecaller. It presegments the signal in homogeneous regions using a window-based *t*-test. The events reduce the runtime complexity from segmenting the whole signal to presegmented events. They are assigned with nucleotides using dynamic programming (DP) with an adaptive banding strategy, which further reduces complexity.

For the assignment, Tombo Resquiggle uses a pore model, which holds mean and standard deviation parameters for expected signal values per *k*-mer and pore used for sequencing. Using the presegmented events and expected signal values, Tombo Resquiggle calculates and sums up *z*-scores for mapped nucleotides. Afterward, the optimal segmentation is taken by following the path that maximizes the sum of *z*-scores. Tombo Resquiggle does not provide any probability or confidence value for its segmentation.

### f5c eventalign and f5c resquiggle

The f5c (v1.5) [8] package is an optimized reimplementation of nanopolish [21]. It also uses a forward pass of an HMM to segment the ONT signal. F5c has 2 modes: f5c Eventalign, which is similar to Tombo Resquiggle and requires a mapping of the reads to a reference sequence. The other mode, f5c Resquiggle, segments the signal using the basecalled reads directly, without any mapping to a reference sequence. Otherwise, both modes work the same way using *k*-mer–based pore models and are similar to Tombo Resquiggle. They also presegment the ONT signal using Scrappies event detection algorithm, with individual parameters, to reduce runtime complexity.

Additionally, f5c uses a different signal normalization. It is performed using a method-of-moments approach to adjust the pore model’s mean and variance to fit the observed pico ampere (pA) signal. Segmentation is performed on the events and signal using DP with an adaptive banding strategy. Within the HMM forward pass, emission probabilities are calculated using the Gaussian probability density function (PDF), while transition probabilities are estimated based on the ratio of detected events to reference *k*-mers.

F5c does not report any probability or confidence value for its segmentation. Alarmingly, low-quality and unmapped reads are neglected: they are excluded from further analysis and are not included in the output segmentation. Additionally, some reads are not fully segmented and can be truncated (Fig. 2).

### Uncalled4 utilizes dynamic time warping

Uncalled4 (v4.1) [9] segments the ONT signal using DTW and the basecaller’s optional low-resolution segmentation. It has 2 modes, similar to f5c Eventalign and f5c Resquiggle, one that requires a mapping of reads to a reference sequence and one without. We were not able to use the segmentation mode without mapping, which was raised as an issue in April 2025, so in this work, we focus on the mode that requires mapping.

Uncalled4 uses the mapping for error correction. Afterward, it employs Scrappies event detection algorithm with custom parameters. Uncalled4 performs a reference-guided signal normalization using a method-of-moments approach. This technique scales the event’s current level to match the expected mean and standard deviation of the corresponding reference *k*-mer, as determined by the Dorado segmentation. It then uses DTW through a DP approach that minimizes the absolute distance between the observed event current and expected current. Uncalled4 uses an adaptive banding strategy derived from the basecaller segmentation, which reduces runtime complexity. The authors state that by using the basecaller segmentation, they can be even more restricted with their band width than existing methods, making the algorithm more efficient. Finally, a linear regression step refines the segmentation, further improving the accuracy in modification detection and reference mapping. Uncalled4 does not report any probability or confidence value for its segmentation.

### Dynamont: Our HMM-based segmentation tool

Dynamont (v0.7.1) is a novel segmentation tool that leverages an HMM to probabilistically align basecalled nucleotide sequences *N* to nanopore sequencing signals *T*. Unlike existing approaches that rely on event detection and forward-only algorithms, Dynamont implements the full Baum–Welch algorithm [22, 23], performing both forward and backward passes to obtain the maximum *a posteriori* (MAP) alignment between *N* and *T*. This enables more flexible and robust segmentation, especially beneficial for low-quality reads, short sequences, or regions affected by nucleotide modifications.

**Figure 4:**
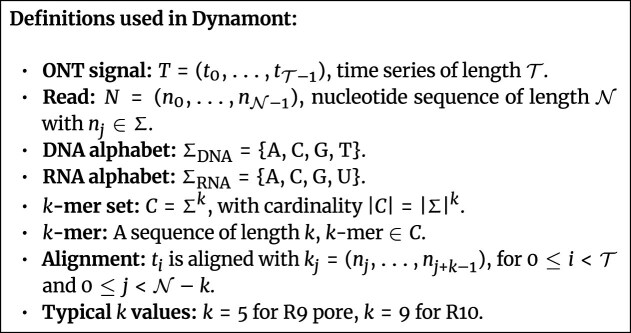
Definitions relevant to the Dynamont segmentation algorithm.

In the ONT context, the sequencing signal *T* represents a time series of current measurements generated as nucleotides translocate through a biological nanopore (Fig. 4). These observations are modeled as emissions from hidden states corresponding to overlapping *k*-mers in the nucleotide sequence. Transition probabilities govern state changes between *k*-mers, while emission probabilities are derived from *k*-mer–specific Gaussian distributions provided by ONT’s pore model.

A distinguishing feature of Dynamont is that it does not use predefined event detection to segment the signal. While this increases computational complexity, it allows for more accurate determination of segment boundaries. Additionally, Dynamont calculates posterior probabilities for each aligned *k*-mer and signal segment, offering quantitative confidence scores (Fig. 2). These scores reflect the model’s certainty in assigning a nucleotide to a segment and can be aggregated or visualized to provide further insights and capabilities not available in competing tools.

Dynamont is modular and includes several functionalities: a basic segmentation mode (used in this study), a resquiggle mode that jointly performs segmentation and error correction (Supplementary Section S2.5), and a training module that enables users to retrain *k*-mer emission and transition parameters on custom data using the Baum–Welch algorithm (Supplementary Section S2.6). This flexibility makes Dynamont highly adaptable to different datasets and sequencing chemistries, providing a robust foundation for downstream analyses such as RNA modification detection, error source identification, and signal interpretation.

#### Emission probability using the Gaussian PDF

ONT signals are influenced by the nucleotide context within the pore and noise in measurement, leading to approximately Gaussian-shaped distributions. To efficiently model this variability, the Gaussian PDF $\phi$, defined in Eq. 1, is used to describe the likelihood of observing a given signal *t* for a specific *k*-mer.


(1) \begin{eqnarray*}
\phi (t, \mu _k, \sigma _k) = \frac{1}{\sqrt{2 \pi \sigma _k^2}} \exp \left(-\frac{(t - \mu _k)^2}{2 \sigma _k^2}\right)
\end{eqnarray*}

For each *k*-mer in the nucleotide sequence *N*, the mean current $\mu _k$ and standard deviation $\sigma _k$ are provided by ONT as part of publicly available *k*-mer models. These parameters define the expected signal characteristics for the different sequencing chemistries. The Gaussian PDF assigns a probability to each pair of signal *t* and *k*-mer, allowing HMMs to evaluate how well a particular *k*-mer explains the observed signal. In Dynamont, $\phi$ is used to calculate emission probabilities, ensuring a probabilistic relationship between the observed signal *T* and the underlying sequence *N*.

#### Dynamont forward-backward algorithm

**Figure 5:**
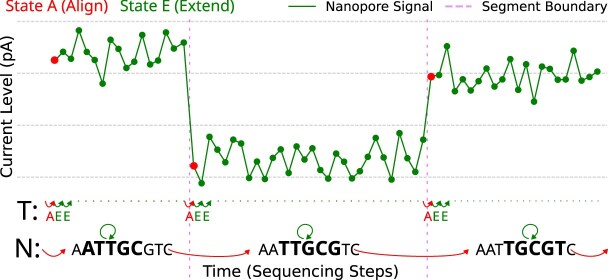
The forward pass functionality of Dynamont: The iteration starts at $t_0$ of signal *T* and $k_0$ of nucleotide sequence *N*. It ends at $t_{\mathcal {T}}$ and $k_{\mathcal {N} - k}$.

Dynamont’s HMM consists of the states *A* (align) and *E* (extend) (Fig. 5). State *A* moves (M) in both dimensions *N* and *T*, marking the start of a new segment, while *E* moves in *T* and halts (H) in *N*, extending a segment (Eqs. 2–3).

Between the states, the transitions $a1$, $e1$, and $e2$ exist. $a1$ starts a new segment. $e1$ is the first extension of a segment, and $e2$ further extends the segment. This model architecture limits the minimum segment size to 2 data points of *t*. State emissions are calculated as shown before with Eq. 1 for both states.

The forward algorithm $\alpha$ of Dynamont iterates *N* and *T* from $t_0$ and $k_0$ to $t_{\mathcal {T}-1}$ and $k_{\mathcal {N}-k}$. For each iteration, $\alpha$ calculates a score using the paths that could be taken to end up in the current state, Eq. 2 [22, 23]. Within the dynamic programming implementation, a matrix exists for each state. The position $i,j$ in each matrix stores the alignment score for the alignment of $(n_0, \dots , n_{j+k-1})$ and $(t_0, \dots , t_i)$. The emission to match *k* and *t* is denoted by $$[ {\begin{array}{l}k_j\\t_i\end{array}}]$$. The initial position is $M_{0,0}$, denoted by $$[{\begin{array}{l}\epsilon _{j=0}\\\epsilon _{i=0}\end{array}}]$$. The final alignment score *Z* can be found in $E_{\mathcal {N}-k,\mathcal {T}-1}$.


(2) \begin{eqnarray*}
A:\left(\begin{array}{l}M_j\\M_i\end{array}\right) &\rightarrow {a1} & \left(\begin{array}{l}H_{j-1}\\
M_{i-1}\end{array}\right) \left[{\begin{array}{l}k_j\\
t_i\end{array}} \right] \left|\vphantom{{\begin{array}{l}k_j\\
t_i\end{array}} }\right.\! \, \left[{\begin{array}{l}\epsilon _{j=0}\\
\epsilon _{i=0}\end{array}} \right] \\
E:\left({\begin{array}{l}H_j\\
M_i\end{array}} \right)&\rightarrow {e1}& \left( {\begin{array}{l}M_j\\
M_{i-1}\end{array}} \right) \left[{\begin{array}{l}k_j\\
t_i\end{array}} \right]\left|\vphantom{{\begin{array}{l}k_j\\
t_i\end{array}} }\right.\, {e2} \left({\begin{array}{l}H_j\\
M_{i-1}\end{array}} \right) \! \!\left[{\begin{array}{l}k_j\\
t_i\end{array}} \right]
\end{eqnarray*}

State *A* can be reached only from state *E*; therefore, only 1 transition $a_1$ exists. The probability of this transition is calculated by multiplying the value of state *E* at position $(j-1, i-1)$ with the emission $$[{\begin{array}{l}k_j\\t_i\end{array}}]$$ and the transition probability $a1$.

State *E* can be reached from both states *A* and *E*. Accordingly, its probability is obtained as the sum of both possible paths. In each case, the previous value is taken at position $(j, i-1)$, once from state *A* and once from state *E*, and multiplied by the emission $$[ {\begin{array}{l}k_j\\t_i\end{array}}]$$ and the corresponding transition probability. The resulting values are then summed to yield the final probability in *E*.

The backward algorithm $\beta$ of Dynamont is derived from the forward algorithm. It is the second pass over the data, iterating from $t_{\mathcal {T}-1}$ and $k_{\mathcal {N}-k}$ toward $t_0$ and $k_0$ [22, 23]. The backward algorithm calculates a score using the paths that could be taken next, starting from the current state. Calculating the backward pass is necessary to obtain the posterior probabilities, Eq. 4. With these, segment border probabilities or nucleotide probabilities, as shown in Dynamont (Fig. 2), can be calculated, which is otherwise not possible. The initial position is denoted by $$[{\begin{array}{l}\delta ^{*}_{j=\mathcal {N}-k}\\\delta ^{*}_{i=\mathcal {T}-1}\end{array}}]$$ in position $E_{\mathcal {N}-k,\mathcal {T}-1}$. A final alignment score $Z^{*}$ can be found in $M_{0,0}$.


(3) \begin{eqnarray*}
A^{*} &:& \left({\begin{array}{l}M_j\\
M_i\end{array}} \right)\!\rightarrow {e1} \left( {\begin{array}{l}H_j\\
M_{i+1}\end{array}} \right) \! \!\left[{\begin{array}{l}k_j\\
t_{i+1}\end{array}} \right] \\
E^{*} &:& \left({\begin{array}{l}H_j\\
M_i\end{array}} \right)\rightarrow {a1} \left({\begin{array}{l}M_{i+1}\\
M_{i+1}\end{array}} \right) \left[{\begin{array}{l}k_{j+1}\\
t_{i+1}\end{array}} \right]\left|\vphantom{{\begin{array}{l}k_{j+1}\\
t_{i+1}\end{array}} }\right. \, {e2} \left({\begin{array}{l}H_j\\
M_{i+1}\end{array}} \right) \left[{\begin{array}{l}k_j\\
t_{i+1}\end{array}} \right]\left|\vphantom{ {\begin{array}{l}k_j\\
t_{i+1}\end{array}} }\right. \left[{\begin{array}{l}\delta ^{*}_{j=\mathcal {N}-k}\\
\delta ^{*}_{i=\mathcal {T}-1}\end{array}} \right] \\
\end{eqnarray*}

To reduce the number of calculations, the forward and backward algorithm of Dynamont can be calculated only for a band (Supplementary Fig. S4). Using this band, *N* is not fully iterated, which reduces the runtime complexity and memory usage.

### Alignment extraction using MAP path

Posterior probabilities $P_Q$ are computed for each state *Q* of the forward algorithm and its corresponding state $Q^{*}$ of the backward algorithm. To normalize these probabilities, they are standardized by the final alignment score *Z*, as defined in Eq. 4:


(4) \begin{eqnarray*}
P_{Q_{i,j}} = \frac{Q_{i,j} Q^{*}_{i,j}}{Z}
\end{eqnarray*}

The MAP path is then extracted using $P_{Q_{i,j}}$ with the Viterbi algorithm [23]. This path represents the most probable alignment of the sequence between *N* and *T*, providing the optimal alignment solution (Supplementary Fig. S3).

## Metrics

To evaluate the segmentation tools, we introduce metrics that will measure i) how well a segment is placed, ii) how many reads are (fully) processed, and iii) how well subsequent tools can work with the output of the segmentation tools. We calculate the scores for only those reads, which were segmented by all tools for each dataset.

**Figure 6:**
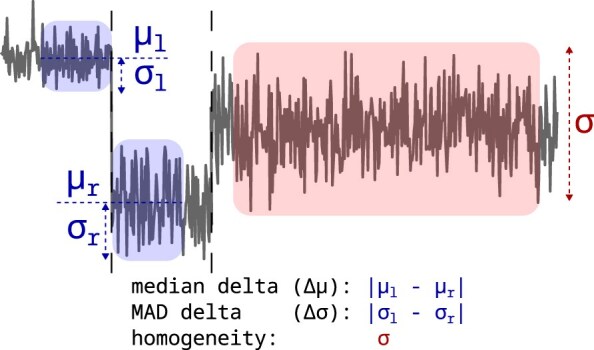
Placements of segments: The median and MAD differences of the window’s left (width: 6 samples) and right (width: 6 samples) to a placed segment are calculated (blue). A higher difference is better, as nucleotide-specific signal segments should, at least in most cases, differ in signal level and variation from one another. A window size of 6 was chosen because it matches the smallest segment size used by Dorado, f5c Eventalign, and f5c Resquiggle. Additionally, the homogeneity is calculated in a window within a segment (red). Since a segment should be internally homogeneous, a lower value is preferable. The homogeneity window covers the inner 80% of each segment, chosen to exclude signals transitioning from one segment to the next when calculating the standard deviation.

To answer how well segments are placed, we calculated the median delta, MAD delta, and segment homogeneity (Fig. 6). Our assumption is that nucleotides within the nanopore produce characteristic signals with respect to intensity and variation. A well-placed segment should, therefore, separate regions within the signal that differ in the signal level and variation. In other words, segments separate heterogeneous regions of the signal, while containing homogeneous regions. As a segment is flanked by 2 borders, for each border, we looked at 6 signal data points left and right of it, collected the median and MAD of these windows, and calculated the absolute difference between the windows. Both values are expected to be maximized by the tools. As the signal within a segment should be produced by 1 *k*-mer, it should be homogeneous in intensity and variation. To measure the homogeneity, we look at segments that span at least 10 signal data points and calculate the standard deviation of the inner 80%, leaving out data points close to the segment borders. A high homogeneity is equal to a low signal variation and vice versa. The segment homogeneity metric is expected to be minimal. For each of these 3 metrics, many values are calculated per tool and dataset. The median value is reported as a score for each tool and dataset (Table 2).

**Table 2:** Example comparison of segmentation tools on the dataset *H. sapiens* RNA004. The metric score each ranges from 0.0 (worst) to 1.0 (best). Absolute values are subscripted. Tombo Resquiggle could not process RNA004 reads. All segmentation tools use the output of Dorado as input, which often prevents them from surpassing Dorado in read statistics or downstream metrics. As seen in all datasets, dedicated segmentation tools greatly improve the segmentation metrics (median delta, MAD delta, and homogeneity). However, a major drawback for these tools involves the metrics regarding read statistics. Dynamont has the smallest drawback and shows an exception for minimum read length. Notice that the other tools neglect short reads, which can be disadvantageous (e.g., when working with small RNAs). The results of the other datasets can be found in Supplementary Tables S1– S15.

Tool	Dorado	f5c R.	f5c E.	Uncalled4	Dynamont
median delta ($\Delta \mu$)	$0.240_{~15.5}$	$0.915_{~59.0}$	$0.938_{~60.5}$	$0.946_{~61.0}$	$1.000_{~64.5}$
MAD delta ($\Delta \sigma$)	$0.750_{~6.0}$	$0.688_{~5.5}$	$0.688_{~5.5}$	$0.750_{~6.0}$	$1.000_{~8.0}$
homogeneity	$0.000_{~16.0}$	$0.219_{~12.5}$	$0.219_{~12.5}$	$0.188_{~13.0}$	$0.094_{~14.5}$
segmented reads	$1.000_{~10088.0}$	$0.916_{~9245.0}$	$0.881_{~8884.0}$	$0.925_{~9329.0}$	$1.000_{~10088.0}$
truncated reads	$1.000_{~0.0}$	$0.000_{~9245.0}$	$0.041_{~8865.0}$	$0.026_{~9005.0}$	$0.662_{~3124.0}$
min read length	$0.975_{~5.0}$	$0.000_{~201.0}$	$0.627_{~75.0}$	$0.667_{~67.0}$	$0.970_{~6.0}$
n50 read length	$1.000_{~1550.0}$	$0.957_{~1483.0}$	$0.897_{~1391.0}$	$0.932_{~1445.0}$	$0.997_{~1546.0}$
max read length	$1.000_{~111593.0}$	$0.374_{~41777.0}$	$0.086_{~9609.0}$	$0.090_{~10027.0}$	$1.000_{~111594.0}$
flye total length	$1.000_{~100285.0}$	$0.939_{~94190.0}$	$0.789_{~79174.0}$	$0.983_{~98534.0}$	$0.931_{~93379.0}$
flye n50	$0.974_{~2786.0}$	$0.955_{~2732.0}$	$0.986_{~2819.0}$	$1.000_{~2860.0}$	$0.978_{~2797.0}$
flye mean coverage	$1.000_{~7.6}$	$0.980_{~7.5}$	$0.565_{~4.3}$	$0.549_{~4.2}$	$0.993_{~7.6}$
svim structural variants	$1.000_{~4.0}$	$0.000_{~0.0}$	$0.000_{~0.0}$	$0.000_{~0.0}$	$0.000_{~0.0}$
AM score	9.94	6.94	6.72	7.05	9.63

To measure how many reads are (fully) processed by each tool, we compared the numbers and lengths of input reads against the output. We decided to measure the number of segmented reads and truncated reads, as well as the minimal, N50, and maximal length of the resulting reads. For the minimal read length and truncated reads, lower values are better, as they indicate fewer filtered short reads and incomplete reads, respectively. This favors tools that retain a large number of reads without excessive filtering and truncation. For all other metrics, the greater the value, the better.

Finally, we wanted to evaluate how well downstream tools could work with the results of the tools. We converted the output of the tools to fasta files and executed flye [24], an assembly tool, and svim [25], a tool to detect structural variants. For flye, we looked at the total length, N50, and mean coverage of the resulting contigs and used them as metrics. For svim, we looked at the number of structural variants svim could find and used it as a metric. These metrics evaluate the possibility of additional data processing after segmentation.

### Converting the metrics to scores

To enable fair comparisons across different metrics and datasets, each metric is converted into a normalized score ranging from 0 to 1. For every tool and dataset, we determine the metric’s maximum values and scale the interval from 0 to the maximum value linearly, such that 0 corresponds to a score of 0 and the maximum value to a score of 1. For metrics where lower values indicate better performance, such as minimal read length or number of truncated reads, the scores are inverted so that lower values yield higher scores (i.e., a raw value of 0 maps to a score of 1.0 and the maximum raw value maps to 0.0). This transformation is applied consistently across all tools and datasets, allowing for a unified and intuitive performance comparison (see Table 2).

### The aggregated metric score

**Figure 7:**
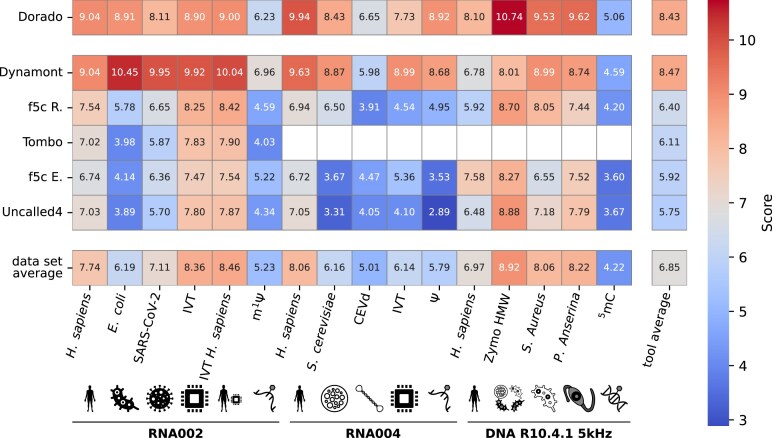
The aggregated metric score (AM score) for each tool and dataset. Tools are sorted for their mean value across all datasets. The AM score is the sum of all metric scores, as shown in Table 2. It describes the overall segmentation performance per tool and dataset. The best average AM score (last column) across all segmentation tools (except Dorado, which we do not declare a segmentation tool) is achieved by Dynamont.

To obtain an overall performance measure, we compute an aggregated metric (AM) score for each tool and dataset. This score is calculated as the sum of all individual normalized metric scores for the respective tool on the given dataset. By aggregating the metric scores in this way, we capture a tool’s overall performance across multiple evaluation criteria in a single value, facilitating direct comparisons between tools. A higher aggregated score indicates better performance across the evaluated metrics for the respective tool and dataset (see Fig. 7).

#### Computational resources

The calculations were executed on a single machine with a 12-core AMD Ryzen 9 3900X CPU, the 6.1.0-30-amd64 x86_64 Kernel; 4 dual in-line memory modules of Kingston 64 GB 2,666 MHz; and a PRIME B550-PLUS Mainboard. Each tool was executed using 20 threads.

#### Usability

We installed all tools without root privileges, using either Conda or precompiled binaries, on a Debian GNU/Linux 12.11 (64-bit) system. The ease of installation and execution varies significantly depending on the user’s system setup, experience, and familiarity with the programming languages the tool relies on. Despite these factors, publicly available software should strive to be as user-friendly as possible. To support this, we documented our experience installing and running each tool, summarized in Table 3.

**Table 3:** Overview of segmentation tool performance. For runtime and memory, we report the minimum, maximum, and median statistics of the tools across the 16 datasets. Dorado utilizes the GPU for the calculations, which is why we reported the virtual RAM (VRAM) in parentheses in addition to the RAM usage. To ensure comparability, both f5c Eventalign and f5c Resquiggle were executed on the CPU, though GPU execution is possible and results in faster runtimes. Runtime and memory measurements were performed on a system with CPU: 12-core AMD Ryzen 9 3900X; Kernel: 6.1.0-30-amd64 x86_64; RAM: Kingston 64 GB 2,666 MHz; and Mainboard: PRIME B550-PLUS, GPU: NVIDIA GeForce RTX 2080 Ti. We assessed the usability (“Use”) of each tool by expressing our experience through emojis. All tools were easily installable. For f5c Resquiggle and f5c Eventalign, the input data format needed to be converted from the state-of-the-art pod5 to the older slow5 format. Tombo Resquiggle requires the input data to be converted to an even older single fast5 format. Additionally, the output format is stored in the same (user-unfriendly) format.

		Runtime			RAM usage in GB (VRAM usage)				
Segmentation Tool (Ver)	Use	Min	Max	Median	Min	Max	Median	Source	Year
Dorado (0.9.6)	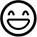	31s	11m 11s	1m 15s	1.1 (6.8)	3.2 (11.0)	2.5 (10.5)	–	2025
Tombo Resquiggle (1.5.1)	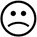	1m 13s	10m 45s	6m 54s	2.1 (0.0)	68.3 (0.0)	2.4 (0.0)	–	2017
f5c Resquiggle (1.5.0)	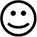	1s	59s	10s	0.1 (0.0)	2.2 (0.0)	0.4 (0.0)	[8]	2020
f5c Eventalign (1.5.0)	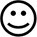	3s	7m 43s	31s	$<$ 0.1 (0.0)	4.8 (0.0)	0.6 (0.0)	[8]	2020
Uncalled4 (4.1.0)	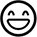	14s	2m 23s	25s	2.5 (0.0)	15.9 (0.0)	5.3 (0.0)	[9]	2024
Dynamont (0.7.2)	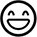	6m 39s	6h 59m 16s	42m 52s	10.3 (0.0)	62.2 (0.0)	39.5 (0.0)	–	2025

## Analyses

We analyzed 16 datasets using 5 segmentation tools and the basecaller Dorado, evaluating performance across 12 metrics. The datasets cover a broad range of sequencing contexts, including 6 DNA samples sequenced on the R10.4.1 pore using the DNA 5-kHz protocol, 5 RNA samples sequenced on the new RNA flow cells using R10.4.1 and the RNA004 protocol, and 5 RNA samples sequenced on the R9.4.1 pore using the RNA002 protocol. Our comparison provides detailed insights into how each segmentation tool performs across these diverse datasets and metrics. The datasets were selected to represent not only different biological origins—from various kingdoms of life—but also different sequencing strategies used in ONT workflows. Our metrics assess 3 key aspects of tool performance: the quality of signal segmentation, the efficiency of read processing, and the compatibility with downstream analysis workflows. For each combination of dataset and metric, we normalized the raw results from all tools to a 0.0–1.0 range. This approach, similar to a *z*-score transformation followed by min–max normalization, ensures fair comparisons across datasets, tools, and metrics (Table 2). Additional detailed tables for all datasets are provided in the Supplementary Tables S1–S15. Finally, for each tool and dataset, we computed an AM score by summing the individual normalized scores, corresponding to the last row in Table 2. This allows a concise, high-level comparison of overall performance across all metrics (Fig. 7).

### Segmentation tool performance is diverse regarding different datasets and quality metrics

All evaluated tools are summarized in Fig. 3 and Table 3. While tuning each tool for optimal performance on every dataset would be ideal, it falls beyond the scope of this evaluation. To ensure a fair comparison, we used each tool’s default settings and adjusted only those parameters that minimize read filtering, where possible. The exact execution commands are provided in Supplementary Section S2.1. In practical applications, we recommend testing multiple parameter configurations, tailored to specific datasets and goals. In case of tools that require mapping, we also recommend trying different reference sequences and mapping parameters for optimal processing. For cases where a tool could not run on a particular dataset or in a specific mode, we made an effort to debug the source code and contacted the authors for support. Based on these experiences, we have evaluated the ease of installation and execution for each tool in Table 3.

### Dorado

Dorado can generate a low-resolution segmentation using its “move table” when the –emit-moves parameter is enabled. However, the resulting segmentation is less precise compared to dedicated segmentation tools, as shown in Fig. 2 and seen in the segmentation metrics (Table 2). The segmentation boundaries derived from the Dorado “move table” often fall within regions of relatively stable signal intensity, rather than aligning precisely with the abrupt current shifts or distinct changes in signal behavior that typically mark true nucleotide transitions.

For the *H. sapiens* RNA004 dataset, Dorado performs worst in segmentation accuracy, with a median delta score of just 0.24—approximately 4 times lower than the other tools—and a homogeneity score of 0.0, as reported in Table 2. Although its raw homogeneity value is similar to that of other tools, its contrast score lags significantly behind. Dorado’s MAD delta score reaches 0.75, comparable to all tools except Dynamont. Because Dorado’s basecalled reads serve as input for all other segmentation tools, it naturally achieves the highest scores for metrics related to read length and count. This dependency also explains Dorado’s strong performance in metrics evaluating downstream tool compatibility.

Dorado’s strong performance in both read-level and downstream tool metrics contributes to its consistently high AM scores across all datasets (Fig. 7). It achieves the a high average AM score overall, with a value of 8.43. However, since Dorado’s basecalled reads serve as the input for all segmentation tools, its performance is expected to be among the top tools. For this reason, we display Dorado separately in Fig. 7 and exclude it from detailed comparisons with the segmentation tools. Despite its strengths, Table 2 shows that Dorado underperforms in key segmentation metrics, highlighting its limited ability to achieve high accuracy in signal segmentation.

### Tombo Resquiggle

Tombo Resquiggle also uses a forward-pass-only HMM for segmentation, preceded by an event detection step that preprocesses the raw signal. However, since Tombo Resquiggle is officially deprecated and no longer maintained, it is excluded from the analysis of the RNA004 *H. sapiens* dataset presented here. Despite this, we chose to include Tombo Resquiggle in our broader study for completeness and historical comparison. In the RNA002 datasets (Supplementary Tables S1–S4), Tombo Resquiggle performs comparably well to other tools. It records the lowest AM score in 1 of 5 datasets and ranks second to last in the *E. coli* dataset. These results reflect both its aging architecture and its limitations in handling more recent data and chemistries. Tombo Resquiggle is installable via Conda but requires multiple data conversions, as it requires single fast5 files as input. The output of Tombo Resquiggle is also stored in the fast5 format, which is not human readable as, for example, tabular csv files.

### f5c Resquiggle

f5c Resquiggle is a segmentation tool that leverages event detection and a forward-only HMM to align and segment the ONT signal directly using basecalled reads (in fastq format). f5c Resquiggle significantly improves segmentation performance on the RNA004 *H. sapiens* dataset compared to Dorado, achieving a median delta score of 0.915 and a MAD delta score of 0.688, as shown in Table 2. While slightly behind the top-performing segmentation tools in these metrics, it performs well in homogeneity, tying with f5c Eventalign at a leading score of 0.219. f5c Resquiggle filters approximately 10% of the reads and exhibits the highest rate of truncation among all tools. Many short reads are excluded while the dataset includes reads as short as 5 nt; the shortest read segmented by f5c Resquiggle is 201 nt. Similarly, the longest read in the dataset (111,593 nt) is only segmented up to 41,777 nt by f5c Resquiggle. As a result, the N50 of segmented reads is reduced by about 5%. Despite the filtering and truncation, f5c Resquiggle performs very well in downstream tool compatibility, achieving scores ranging from 0.85 to 0.98, indicating robust integration in typical ONT analysis workflows.

f5c Resquiggle consistently ranks among the top segmentation tools across all datasets. In terms of the AM score, it secures second place among segmentation tools with an average score of 6.40. Its best performance is observed in both of the RNA002 IVT datasets. Additionally, it ranks second in multiple datasets, including RNA002 *E. coli* and SARS-CoV-2, RNA004 *H. sapiens, S. cerevisiae*, and IVT, as well as the DNA Zymo HMW and *S. aureus* datasets (Fig. 7).

### f5c Eventalign

f5c Eventalign closely resembles f5c Resquiggle in its segmentation approach, relying on event detection and a forward-only HMM. However, unlike f5c Resquiggle, which segments the signal directly using basecalled reads, f5c Eventalign requires reads to be mapped to a reference genome prior to segmentation. This additional mapping step slightly improves segmentation in some metrics, such as achieving a higher median delta score of 0.938 on the RNA004 *H. sapiens* dataset. The MAD delta and homogeneity scores remain unchanged at 0.688 and 0.219, respectively—-identical to those of f5c Resquiggle. Notably, like other tools, f5c Eventalign outperforms Dorado in signal segmentation, achieving a nearly 4 times higher median delta score. In terms of read-level performance, f5c Eventalign filters out more reads than f5c Resquiggle due to its dependence on mapping. While it truncates a significant number of reads, it retains more short reads than f5c Resquiggle, with the shortest segmented read being 75 nt long. However, a major drawback is its handling of long reads. The longest segmented read in this dataset is only 9,609 nt—just 8.6% of the true longest read (111,593 nt). This limitation stems from the initial mapping step, as demonstrated by f5c Resquiggle’s ability to segment reads over 41,000 nt in length. f5c Eventalign shows mixed performance in downstream tool compatibility. For example, flye assemblies generated from its outputs are shorter in total length and coverage, though the N50 is improved. Structural variant detection using svim also identifies more variants, likely due to base modifications introduced during the mapping step, which alters the nucleotide sequences.

On average, f5c Eventalign ranks third among segmentation tools, with an AM score of 5.92 across all datasets (Fig. 7). It achieves the best overall score on the DNA *H. sapiens* dataset, outperforming the second-best tool by 12 %.

### Uncalled4

Uncalled4 utilizes event detection for preprocessing but differs from other tools by applying a forward-pass DTW algorithm instead of an HMM for segmentation. Like f5c Eventalign, it requires a prior mapping step before segmentation can begin. In the RNA004 *H. sapiens* dataset, Uncalled4 achieves higher segmentation accuracy than both f5c Eventalign and f5c Resquiggle. It reaches a median delta score of 0.946 and a MAD delta of 0.75. The homogeneity score is slightly lower at 0.188, though still within a competitive range. From a read-level perspective, Uncalled4 segments 9,329 reads—more than f5c Resquiggle—indicating strong performance despite the mapping prerequisite. The shortest successfully segmented read is 67 nt long. Truncation rates and N50 values are comparable to those of f5c Eventalign and f5c Resquiggle. However, the longest segmented read (10,027 nt) is 91% shorter than the dataset’s longest read, likely due to limitations imposed by the mapping step shared with f5c Eventalign. In downstream analysis, Uncalled4 delivers competitive results. Its assembly from flye has the second-longest total length after Dorado, and it achieves the highest N50 of 2,860. The mean coverage, however, remains on the lower end, similar to f5c Eventalign. Notably, Uncalled4 receives the lowest score (0.549) in the svim structural variant detection metric, suggesting limitations in preserving variant-relevant features.

Across all datasets, Uncalled4 attains an average AM score of 5.75, placing it last among the segmentation tools evaluated (Fig. 7). It performs particularly well in the DNA Zymo HMW dataset, where it achieves the highest overall score and ranks second in the *P. anserina* dataset.

### Dynamont

Dynamont does not rely on event detection or premapping. Instead, it directly segments the ONT signal using a forward and backward pass of a custom HMM, aligning the signal to the basecalled reads. Dynamont achieves the highest segmentation quality among all tools, with a median delta approximately 6% higher and a MAD delta around 33% higher than the next best method. Its homogeneity score is slightly lower than the top-performing tools but still outperforms Dorado and remains competitive overall. Dynamont exhibits excellent performance in preserving read integrity. It segments all 10,088 reads without discarding or truncating a significant portion, resulting in roughly 66% fewer truncations than other tools. It is able to process both very short reads (as short as 6 nt) and very long reads (up to ~111,500 nt). As a result, its N50 read length (1,550 nt) closely matches the input read length distribution provided by Dorado. Although slight differences in read structure lead to a marginally shorter total assembly length with flye (93,379 nt), Dynamont produces a slightly higher assembly N50 (2,797 nt) and near-identical coverage (score: 0.993). Still, Dynamont reaches the second highest AM score on the RNA004 *H. sapiens* dataset with a score of 9.63.

Across all datasets, Dynamont achieves the highest average AM score among the segmentation tools, with a score of 8.47, approximately 32% higher than f5c Resquiggle. Dynamont performs best in all RNA002 datasets, all RNA004 datasets, and the DNA *S. aureus, P. anserina*, and $^5$mC datasets. In 7 of 11 RNA datasets, Dynamont outperforms Dorado itself.

## Tool Usability and Runtime Performance

We evaluated each tool not only by its analytical performance but also in terms of usability, runtime, and memory efficiency. These practical aspects are especially important when deploying signal segmentation pipelines on diverse systems or large datasets. Our experiences are summarized in Tab 3. These results do apply for the presented datasets with a read count of 10 000 reads per dataset and their specific read statistics, Table 1.

### Installation and setup

All tools (Tombo Resquiggle, f5c Resquiggle, f5c Eventalign, Uncalled4, and Dynamont) were easy to install via Conda or as precompiled binaries, with minimal setup overhead. Dorado, in particular, was easy to deploy, offering official binaries and comprehensive documentation on GitHub. However, several tools lacked compatibility with ONT’s current pod5 file format. In particular, Tombo Resquiggle, f5c Resquiggle, and f5c Eventalign required conversion to other file formats, adding a preprocessing step. By contrast, Uncalled4 and Dynamont support pod5 directly, reducing preprocessing complexity and improving user experience. Execution commands are generally well documented. Especially for tools that require input format conversion, command-line execution was more complex but manageable with guidance provided in Supplementary Section S2.1.

### Runtime and memory efficiency

Runtime and memory usage varied significantly across tools. f5c Resquiggle was the fastest, completing all datasets in under 1 minute and requiring at most 2.2 GB of RAM. f5c Eventalign had a longer runtime, up to 7 minutes and 43 seconds, and slightly higher memory usage. Uncalled4 performed moderately in runtime but showed a higher peak in memory usage at 15.9 GB. Dorado, while primarily a basecaller, exhibited longer runtimes (up to 11 minutes on a GPU-enabled system) due to the overhead of neural network inference. Nevertheless, it remained within reasonable limits for most workloads, utilizing 1.8 to 3.2 GB of RAM. Dynamont, implementing a full forward–backward HMM alignment without prefiltering or event detection, was the most computationally intensive. It required up to 7 hours of runtime and higher memory consumption, trading efficiency for improved segmentation accuracy and flexibility. These trade-offs should be carefully weighed when choosing a segmentation tool depending on project size, available computational resources, and desired output fidelity.

For all evaluated algorithms, runtime and memory usage scale with both the number and length of input reads. Longer reads require more computation during segmentation, leading to a runtime that increases linearly with read length. Similarly, a larger number of reads proportionally extend total execution time. Memory usage also grows linearly, as all tools implement DP approaches—typically using banded matrices, where one dimension remains fixed relative to the input data. Another influence for the memory can be the number of processes used in parallel (e.g., Dynamont calculates multiple reads in parallel when using more than 1 process).

## Discussion

The heatmap in Fig. 7 provides an overview of the aggregated performance of 5 segmentation tools and Dorado across 16 diverse datasets covering RNA002, RNA004, and DNA R10.4.1 5-kHz chemistries. Each cell in the matrix reflects the average normalized score across a range of metrics designed to capture segmentation quality, read retention, and downstream usability.

### Dorado as a baseline: High compatibility but limited segmentation precision

Dorado, while not a segmentation tool in the strictest sense, consistently achieves very high AM scores across nearly all datasets, with an overall average score of 8.43. This is largely due to its role as the basecaller whose output serves as the input for all other tools. As such, it retains all reads without truncation and shows strong compatibility with downstream tools. However, as mentioned earlier, its segmentation metrics—particularly median delta and homogeneity—are considerably lower across all datasets. This underscores that Dorado, in the strictest sense, is not a segmentation tool. Its primary objective is to translate the ONT signal into nucleotide sequences, without explicitly modeling the assignment of individual nucleotides to signal segments (see Fig. 2). This is evident in the segmentation metrics and in Fig. 2, Table 2, and Supplementary Tables S1– S15, making it a less suitable candidate for direct signal interpretation.

### Tombo Resquiggle: Limited support for modern chemistries

Tombo Resquiggle, the oldest tool included, underperforms with an average score of 5.55. It ranks last in 2 of the 4 RNA002 datasets and, due to its deprecation, lacks support for modern chemistries. Its reliance on outdated formats and limited flexibility in signal handling significantly reduce its usability and performance.

Tombo Resquiggle’s median delta scores are higher than Dorado’s but lower than those of other segmentation tools across all RNA002 datasets. Its MAD delta consistently ranks high (Supplementary Tables S1–S4), achieving the best score in the SARS-CoV-2 dataset, while its homogeneity score rarely surpasses that of other tools. The number of segmented and truncated reads is comparable to other segmentation tools, and it achieves the second shortest minimum read length. Overall, Tombo Resquiggle performs best in the MAD delta metric for the SARS-CoV-2 dataset. Tombo Resquiggle’s comparatively low segmentation performance likely stems from its early development as one of the first segmentation tools released in 2017 (Table 3). Subsequent tools have benefited from additional years of development, allowing for refinement of models, tuning of parameters, and building upon the foundational work introduced by Tombo Resquiggle to achieve improved segmentation of the ONT signal.

### f5c Resquiggle: Efficient and homogeneous segmentation but unsuitable for short reads

f5c Resquiggle ranks second among the segmentation tools, with an average score of 6.40 (Fig. 7) showing a strong performance in read retention and moderate segmentation quality. It benefits from fast runtime and low memory usage, making it practical for large datasets. However, it relies on Scrappie’s event detection, which may limit its segmentation flexibility. f5c Resquiggle consistently achieves the highest homogeneity scores across all evaluated datasets and appears particularly effective at segmenting homogeneous regions of the signal.

Surprisingly, f5c Resquiggle appears to impose a lower bound on the minimum read length it segments. Across all evaluated datasets, the shortest segmented read consistently measures either 200 nt, resulting in a uniform score of 0.0 for this metric. We could not identify a command-line parameter to modify this cutoff. This limitation has important implications: users working with short reads ($<$200 nt) should avoid using f5c Resquiggle, as it will systematically exclude these sequences from segmentation. Interestingly, f5c Resquiggle also shows a tendency to retain longer reads in DNA datasets more effectively than in RNA datasets, which could be caused by generally higher read qualities in ONT DNA sequencing.

f5c Resquiggle segments a smaller fraction of reads in RNA datasets compared to DNA datasets. While f5c Resquiggle consistently segments over 95% of reads in all DNA datasets—with the remaining reads not segmented and effectively neglected—the segmentation rate for RNA varies widely, from as low as 16% in the CEVd dataset to 91% in the RNA004 *H. sapiens* dataset. This discrepancy is likely driven by differences in read quality and length (Table 1), which are influenced by the sequencing protocol, pore type, sample origin, and the presence of diverse RNA modifications in *in vivo* samples.

### f5c Eventalign: High-precision median delta segmentation with mapping-induced read loss

f5c Eventalign, a close relative of f5c Resquiggle that incorporates mapping, achieves a slightly lower overall score (5.92) and performs particularly poorly on datasets with high signal variation or mapping difficulty, such as SARS-CoV-2, CEVd, and the datasets with RNA modifications. Its reliance on reference-based alignment causes excessive filtering of long reads, impacting its read metrics and downstream compatibility. f5c Eventalign matches or surpasses f5c Resquiggle’s median delta score in 13 of 16 datasets, being the overall best tool in that metric for the RNA004 CEVd and IVT dataset. The MAD delta is worse in the RNA004 $\Psi$ and DNA Zymo HMW dataset, otherwise either matching or surpassing f5c Resquiggle’s. Due to additional mapping, the number of segmented reads drops slightly compared to f5c Resquiggle, except for dataset RNA002 m$^1\Psi$, which drops by around 90%. Although being related to f5c Resquiggle, f5c Eventalign is able to segment reads shorter than 200 nt, the shortest being 43 nt in the RNA004 CEVd dataset. f5c Eventalign is not as good as f5c Resquiggle in segmenting the signal into homogeneous regions, as seen in the homogeneity metric (Table 2 and Supplementary Tables S1–S15). Compared to f5c Resquiggle, the maximum read length segmented by f5c Eventalign is even shorter, most probably caused by the mapping prior to segmentation, which can introduce additional read filtering and truncation.

### Uncalled4: DTW-based segmentation with high homogeneity in DNA but read length limitations

Uncalled4, with an average score of 5.75, shows moderate performance. It outperforms f5c Eventalign in many RNA datasets and even leads in the DNA Zymo HMW dataset. However, it also suffers from high memory usage and substantial read filtering due to its dependence on mapping. Interestingly, its segmentation quality (in terms of delta scores) rivals that of f5c tools, despite using a different core algorithm (DTW). Uncalled4 has the highest median delta score for all RNA002 datasets. Within the DNA datasets, the tool matches or surpasses the highest homogeneity score of f5c Resquiggle. Overall, Uncalled4 has comparable segmentation metric scores across all datasets. Uncalled4 is in 12 of 16 datasets able to segment more reads compared to f5c Eventalign and f5c Resquiggle. But, its reported max read length is among the shortest across the tools, scoring lowest in 7 datasets and generally not being able to improve a lot compared to f5c Eventalign and f5c Resquiggle. This is possibly caused by the mapping and the similar event detection prior to segmentation.

### Dynamont: Robust full HMM segmentation outperforming existing tools across most datasets

Dynamont clearly stands out among the true segmentation tools. It achieves the highest overall average score (8.47) among the segmentation tools. It demonstrates its versatility by outperforming all tools in 7 of 11 RNA datasets, often even surpassing Dorado, which sets a benchmark in terms of read and compatibility metrics. This confirms Dynamont’s robustness across organism/origins, signal characteristics, and sequencing kits. It consistently ranks first or second in all 11 RNA datasets (Table 2 and Supplementary Tables S1–S10). Although Dynamont does not reach the highest AM score in the DNA *H. sapiens* dataset, the median delta is around 25%, and the MAD delta is 33% higher compared to all other segmentation tools (Supplementary Table S11). This improvement likely stems from skipping event detection and at the same time utilizing not only the forward pass but also the backward pass of the HMM.

By avoiding enforced read filtering, Dynamont is able to segment nearly all reads in all datasets. It attempts to segment even very short or low-quality reads that other tools would typically filter. Through its use of a full forward–backward HMM pass, Dynamont not only takes on the challenge of segmenting these difficult reads but also provides model-derived confidence probabilities for each segment. This enables users to make informed, data-driven decisions about whether to retain or exclude individual segments based on confidence scores. Allowing this level of control is particularly valuable when analyzing reads containing modifications, which often lead to basecalling errors and reduced quality.

Dynamont’s segmentation metrics on the Zymo HMW dataset are notably lower compared to all other tools, a result that contrasts sharply with its otherwise consistently strong performance across datasets. Despite extensive analysis, we were unable to identify a definitive cause for this decline. One potential explanation could be the metagenomic nature of the Zymo dataset, which may pose specific challenges. However, given that this conclusion is drawn from a single synthetic metagenomic dataset, it remains speculative and warrants further investigation using a broader range of metagenomic samples. Its superior segmentation performance is attributed to its full forward–backward HMM implementation, which avoids signal presegmentation and mapping, allowing it to retain more reads, including very short and very long ones, and to truncate less reads. Additionally, its ability to compute posterior probabilities enhances interpretability for the user (Fig. 2), especially for difficult-to-segment signals like modified nucleotides or general basecalling errors.

### Dataset-dependent tool performance: Short and low-quality reads

Tool performance varies across datasets. RNA004 datasets, especially CEVd and IVT, generally yield lower average scores, indicating that these samples present more challenging conditions for segmentation, likely due to shorter reads and lower quality (Table 1). A similar picture can be derived from the *E. coli* dataset from the RNA002 chemistry. This dataset has the shortest reads and quality of RNA002. All tools struggle for this dataset except for Dynamont, which still provides segmentations for a lot of reads. Dynamont outperforms other tools on datasets with shorter reads according to the AM score. The CEVd and IVT reference sequence is very short, which introduces additional difficulties in the flye assembly (Supplementary Table S8 and Supplementary Table S9). Segmentation tools, which require mapping prior to segmentation, show a higher number of truncated and a lower number of segmented reads, which also leads to difficulties in the flye assembly or the detection of structural variants with svim. In contrast, datasets such as DNA Zymo HMW and *P. anserina* consistently yield higher scores across tools, benefiting from higher read quality and length of the DNA sequencing protocol. In general, a trend can be suspected when comparing the scores of Fig. 7 with the read statistics of Table 1: the higher the quality and the longer the reads, the better the AM score. Not only is the AM score higher, but the raw segmentation metric values are also higher in the DNA results for all tools compared to RNA. DNA datasets with longer and better-quality reads seem to be easier to be segmented than RNA reads.

RNA modifications introduce basecalling errors and lead to lower read qualities (Table 1), which imposes a big challenge for all segmentation tools. Overall, the scores drop for the m$^1\Psi$, $\Psi$ and $^5$mC datasets across all tools (Fig. 7). F5c especially filters a lot of reads from the $\Psi$ and $^5$mC datasets, effectively segmenting below 100 reads each. Uncalled4 segments much more, but the read lengths are shortened a lot, probably due to the mapping of reads containing a lot of errors. In these datasets, Dynamont can shine, as it still tries to segment all reads while reaching a high median and MAD delta. Dynamont will also have difficulties segmenting these reads containing basecalling errors and signals of modifications. In this case, the output of Dynamont about the segmentation probability can help the user to evaluate the segment quality.

## Potential Implications

Here, we summarize some key findings for our comparative study: i) No tools was best in all datasets. Depending on the users’ needs, different tools are favorable above others. ii) Users working with short or low-quality RNA reads should avoid tools like f5c Resquiggle, which systematically exclude reads shorter than 200 nt. In such cases, Dynamont offers the best alternative, as it retains nearly all reads with minimal truncation and provides high segmentation accuracy. iii) Projects requiring accurate segmentation in homogeneous signal regions may benefit from using f5c Resquiggle due to its superior homogeneity scores, especially in DNA datasets. If accurate segmentation for heterogeneous regions is more favorable, than Dynamont with its consistently high median delta and MAD delta scores is recommendable. iv) For workflows emphasizing downstream applications (e.g., assembly, variant detection), Dorado offers strong baseline compatibility, but its low segmentation accuracy limits utility in modification detection or fine-grained signal analysis. v) Tools like f5c Eventalign and Uncalled4, while promising, may under perform in datasets requiring robust long-read retention due to mapping-related filtering. vi) Memory and runtime considerations become critical for large-scale studies. Dynamont, while delivering the most accurate results, demands substantially more memory and computational time, which may limit its usability in high-throughput pipelines unless paired with adequate hardware. vii) Dynamont introduces a new benchmark in ONT signal segmentation but highlights the classic trade-off between performance and efficiency. viii) Despite recent progress in modification-aware basecalling from ONT, their models function as black-box end-to-end predictors and do not provide interpretable links between signal and sequence. Signal segmentation remains essential for transparent signal interpretation, independent validation, and downstream analyses such as *de novo* modification detection that depend on precise signal-to-nucleotide alignment.

## Limitations and Future Work

Despite the breadth and depth of our benchmarking study, several limitations must be acknowledged, and they offer opportunities for future work.

In this study, we evaluated each segmentation tool using its default configuration or minimally adapted parameters to ensure fairness and reproducibility. However, tool performance may vary significantly under different parameter settings, particularly when fine-tuning segmentation thresholds or adjusting for read quality. We encourage future studies to investigate the effect of broader parameter sweeps and optimization techniques, ideally tailored to specific datasets or organisms. Similarly, the requirement for read-to-reference mapping prior to segmentation may also influence performance and should be systematically evaluated. While our metrics aim to quantify segmentation performance, access to a ground-truth signal segmentation would provide a more definitive and meaningful evaluation.

### Dataset and chemistry scope

While our evaluation spans 12 datasets across three ONT chemistries (RNA002, RNA004, and DNA R10.4.1), it still represents only a subset of the growing diversity of sequencing protocols and pore types. Future work could extend this comparison by including additional pore chemistries (e.g., R10.4.2 or future releases), newer RNA sequencing kits, and broader organismal diversity, including metagenomic and single-cell datasets.

### Tool and metric expansion

As new tools emerge, our comparison inevitably becomes temporally constrained. We provide a detailed electronic supplement that can be dynamically extended with new segmentation tools, metrics, and datasets in future iterations of this work. This modular approach will support continuous benchmarking and allow researchers to adapt the framework to their specific needs. One limitation of our segmentation metrics is that we currently cannot capture if a tool is able to detect subtle changes in the signal, which result from a transition of *k*-mers in the pore. Currently, larger signal differences at a segment border are rewarded, while smaller ones are penalized. Dynamont often shows a high median and MAD delta, while being worse in the homogeneity score than other tools. Dynamont tends to stack segment borders near larger signal differences, when it could not find a better-fitting segment according to the provided pore (*k*-mer) model. These segments often contain only 2 signal data points and should be handled with care by the user. Such segments will appear more often if the basecalling errors are high and, respectively, the read quality is lower. We tried to reduce their impact on the scores by only selecting segments of a sufficient size for the homogeneity score. Generally, the better basecallers and *k*-mer models get, the better the segmentation algorithms will work in the future.

### Integration into downstream workflows

Ultimately, segmentation tools serve as preprocessing stages for downstream analyses such as RNA modification detection, transcript isoform mapping, or structural variant calling. Future work should evaluate how segmentation quality influences these downstream tasks in a quantitative and biologically meaningful way. Integrating segmentation performance directly into end-to-end pipelines will help determine the practical utility of each method in real-world research applications.

### Toward ensemble segmentation

Our results reveal complementary strengths among the segmentation tools. For example, while f5c Resquiggle achieves the best homogeneity, Dynamont shows superior overall robustness and delta metrics. This suggests a potential benefit in combining tool outputs into an ensemble segmentation approach—similar to ensemble methods in genome assembly or variant calling. Future research could explore consensus segmentation pipelines that select the best segments from each tool or fuse segmentations probabilistically based on alignment confidence and signal characteristics.

### Standardization and ground truth

A key limitation in ONT signal segmentation remains the absence of a validated ground truth. This restricts evaluations to proxy metrics like segmentation similarity, read retention, and downstream compatibility. The development of simulated or experimentally validated benchmark datasets with known signal-to-sequence mappings would greatly enhance tool assessment. We advocate for a community-driven effort to define such standards.

## Conclusion

In conclusion, while this study establishes a foundational benchmark for ONT segmentation tools, it also highlights the dynamic nature of this field. Continued benchmarking, tool refinement, and method integration are essential to ensure robust and accurate interpretation of nanopore sequencing signals.

Dynamont offers a robust approach for nanopore signal segmentation and alignment, particularly excelling in challenging conditions such as short or low-quality reads. Although Dynamont incurs a longer runtime and higher memory usage compared to existing tools, these costs are offset by its ability to segment significantly more reads and provide additional confidence probabilities for each segment. This is an innovation that cannot be found in other methods. These confidence scores, which quantify the reliability of each aligned *k*-mer and signal segment, are invaluable for downstream analyzes, including modification studies, error rate assessment, and source attribution of basecalling errors.

Dynamont achieves higher or comparable segmentation scores in independent datasets across DNA and RNA chemistries, while maintaining similar segmentation homogeneity. Furthermore, Dynamont incorporates a more complex algorithm for error correction (see Supplementary Section S2.5) and offers functionality for training custom *k*-mer models for specific datasets (see Supplementary Section S2.6), providing flexibility for customized analyses.

Future development includes the reduction of runtime and memory complexity, which could be achieved by incorporating a presegmentation step to reduce runtime and memory complexity. This enhancement could diverge from the current Scrappie-based event detection method used by other tools, further optimizing Dynamont for large-scale and real-time applications. Overall, the unique features of Dynamont, its comprehensive segmentation coverage, confidence scoring, and error correction capabilities, position it as a powerful tool for advanced nanopore signal analysis.

## Availability of Source Code and Requirements

- Project name: Dynamont
- Project homepage: https://github.com/rnajena/dynamont
- License: GPL 3.0
- Operating system: Linux-64
- Programming language: Python, C++
- Hardware requirement: at least 32 GB RAM (depends on read length and threads used)
- Biotools: dynamont
- RRID: SCR_027357

Dynamont’s code is available via Zenodo [26], Conda, and Pypi.

## Supplementary Material

giag005_Supplemental_File

giag005_Authors_Response_To_Reviewer_Comments_Original_Submission

giag005_GIGA-D-25-00327_Original_Submission

giag005_GIGA-D-25-00327_Revision_1

giag005_Reviewer_1_Report_Original_Submission Ziyuan Wang -- 9/16/2025

giag005_Reviewer_1_Report_Revision_1 Ziyuan Wang -- 1/5/2026

giag005_Reviewer_2_Report_Original_Submission Daniel Depledge -- 9/25/2025

giag005_Reviewer_2_Report_Revision_1 Daniel Depledge -- 1/7/2026

## Data Availability

The pod5 files containing the 10,000 randomly selected reads for each dataset and our reference sequences are available via Zenodo [27].

## Additional Files

**Supplementary Fig. S1**. The Dorado RNA004 “super accuracy” basecalling model consists of 5 convolution layers, 18 transformer layers, and 2 linear layers. The model outputs a table in the end for each input signal window of 12,000 samples. This table downsamples the 12,000 input signals to 2,000 and holds the model’s prediction for the nucleotide sequence (rows: 1,024 times 4 bases). It can be decoded using algorithms like viterbi or beam search, which will yield the nucleotide sequence for that window. Overlapping nucleotide sequences from overlapping windows are merged later on.

**Supplementary Fig. S2**. Preprocessing overview of the signal. The unnormalized time series (left y-axis) is normalized (right y-axis) to standardize the data. Detected outliers (red circles) mark points that deviate significantly from the expected signal pattern. These outliers can introduce very low probabilities in the HMM forward–backward algorithm. The time series presented here originates from an RNA read. It is sequenced from the 3′ to 5′ end, divided into “Adapter,” “Poly A,” and “Transcript.” A DNA signal would be sequenced from the 5′ to 3′ end and contain a different adapter signal, no “Poly A” region, and a part that looks similar to the “Transcript.” Within a single signal, these segments can appear multiple times, caused by ligated reads or reads that are sequenced nearly instantaneous after each other. Doradowill split these reads during basecalling.

**Supplementary Fig. S3**. Logarithmic probabilities of full (N $\times$ T) Dynamontmatrices, calculated during (a) forward pass, (b) backward pass, and (c) the combined posterior probability. The matrices show the probability flux of the nucleotide sequence (N) to ONT signal (T) alignment, using the HMM implemented in Dynamont. The highest probability can be found around the diagonal, which can be used for further optimization.

**Supplementary Fig. S4**. Logarithmic probabilities of banded DP (N $\times$ T) Dynamontmatrices, calculated during (a) forward pass, (b) backward pass, and (c) the combined posterior probability. The matrices show the probability flux of the nucleotide sequence (N) to ONT signal (T) alignment, using the HMM implemented in Dynamont. The highest probability can be found around the diagonal, which can be used for further optimization.

**Supplementary Fig. S5**. Logarithmic probabilities of reduced banded DP (N $\times$ T) Dynamontmatrices, calculated during (a) forward pass, (b) backward pass, and (c) the combined posterior probability. The matrices show the probability flux of the nucleotide sequence (N) to ONT signal (T) alignment, using the HMM implemented in Dynamont. The highest probability can be found around the diagonal, which can be used for further optimization.

**Supplementary Table S1**. Metrics for the *H. sapiens* RNA002 dataset.

**Supplementary Table S2**. Metrics for the *E. coli* RNA002 dataset.

**Supplementary Table S3**. Metrics for the SARS-CoV-2 RNA002 dataset.

**Supplementary Table S4**. Metrics for the IVT RNA002 dataset.

**Supplementary Table S5**. Metrics for the IVT *H. sapiens* RNA002 dataset.

**Supplementary Table S6**. Metrics for the m1$\psi$ RNA002 dataset.

**Supplementary Table S7**. Metrics for the *S. cerevisiae* RNA004 dataset.

**Supplementary Table S8**. Metrics for the CEVd RNA004 dataset.

**Supplementary Table S9**. Metrics for the IVT RNA004 dataset.

**Supplementary Table S10**. Metrics for the $\psi$ RNA004 dataset.

**Supplementary Table S11**. Metrics for the *H. sapiens* DNA dataset.

**Supplementary Table S12**. Metrics for the Zymo HMW DNA dataset.

**Supplementary Table S13**. Metrics for the *S. aureus* DNA dataset.

**Supplementary Table S14**. Metrics for the *P. anserina* DNA dataset.

**Supplementary Table S15**. Metrics for the $^5$mC DNA dataset.

## Abbreviations

AM: aggregated metric; cDNA: complementary DNA; CEVd: citrus exocortis viroid; DP: dynamic programming; DRS: direct RNA sequencing; DTW: dynamic time warping; HMM: hidden Markov model; IVT: *in vitro* transcribed; MAD: median absolute deviation; MAP: maximum *a posteriori*; mRNA: messenger RNA; ONT: Oxford Nanopore Technologies; pA: pico ampere; PDF: probability density function.
