# Supplementary material for: Dynamont: A comprehensive cross-species comparison of ONT segmentation tools
Source: Gigascience. 2026 Jan 19;15:giag005. doi: 10.1093/gigascience/giag005 (PMC13014473; doi:10.1093/gigascience/giag005)
Supplement: giag005_Supplemental_File [file giag005_supplemental_file.pdf]

S2 Supplemental Material

S2.1 Commands

S2.1.1 Benchmark Preparation Commands

These commands can also be found on protocols.io https://www.protocols.io/view/dynamont-x54v9528pl3e/v1.

```
# Extract 10.000 Random Reads
pod5 view <dataset.pod5> --ids --no-header -o all_ids.txt
sort --random-sort all_ids.txt | head --lines 10000 > 10k_ids.txt
pod5 filter <dataset.pod5> -o <dataset_r10k.pod5> --ids 10k_ids.txt
# Convert To Other Data Formats
blue-crab p2s <dataset_r10k.pod5> -o <dataset_r10k.blow5>
pod5 convert to_fast5 <dataset_r10k.pod5> -o fast5/
multi_to_single_fast5 -i fast5/ -s single_fast5/
# Basecalling
## explicitely using rna002_70bps_hac@v3 for RNA002 data
dorado basecaller sup -x cuda:0 <dataset_r10k.pod5> > <dataset_r10k.bam>
samtools bam2fq <dataset_r10k.bam> > <dataset_r10k.fastq>
dorado summary <dataset_r10k.bam> > sequencing_summary.txt
# converting sequencing summary to tombo format (single fast5)
awk -F'\t' 'NR == 1 {print; next} {$1 = $2 ".fast5"; print}' OFS='\t' sequencing_summary.txt > tombo_sequencing_summary.txt
# Mapping
## preset = splice if RNA and h_sapiens, s_cerevisiae, e_coli, sarscov2
## preset = lr:hq for DNA R10.4.1
## preset = map-ont else
minimap2 <ref.fa> <dataset_r10k.fastq> -x <preset> -a | samtools view -hbF4 | samtools sort > <dataset_r10k_mapping.bam>
samtools index <dataset_r10k_mapping.bam>
```

S2.1.2 Dynamont Segmentation Commands

```
# model can be added explicitely, otherwise default pore model is chosen
python segment.py --raw <path/to/pod5/dataset_r10k/> --basecalls <dataset_r10k.bam> --mode basic -o <dynamont.csv> --pore <pore>
```

S2.1.3 Dorado Segmentation Commands

```
# Basecalling with emit moves
## explicitely using rna002_70bps_hac@v3 for RNA002 data
dorado basecaller sup -x cuda:0 --emit-moves <dataset_r10k.pod5> > <dataset_r10k_moves.bam>
# Extracting moves as segmentation borders
python extractDoradoMoves.py <dataset_r10k_moves.bam> -o <dataset_r10k_moves.tsv>
```

S2.1.4 f5c Segmentation Commands

```
f5c index --slow5 <dataset_r10k.blow5> <dataset_r10k.fastq>
# Eventalign
## added --rna in case of RNA
f5c Eventalign -b <dataset_r10k_mapping.bam> -g <ref.fa> -r <dataset_r10k.fastq> --slow5 <dataset_r10k.blow5> --signal-index --collapse-events --pore <pore> --min-mapq 0 --summary <dataset_r10k_event.sum> > <dataset_r10k_event.tsv>
# Resquiggle
## added --rna in case of RNA
f5c Resquiggle --pore <pore> <dataset_r10k.fastq> <dataset_r10k.blow5> > <dataset_r10k_resqu.tsv>
```

S2.1.5 Tombo Segmentation Commands

```
tombo preprocess annotate_raw_with_fastqs --fast5-basedir single_fast5/ --fastq-filenames <dataset_r10k.fastq> --sequencing-summary-filenames sequencing_summary.txt
# only executed on RNA002
tombo Resquiggle --q-score 0 --rna single_fast5/ <ref.fa>
```

S2.1.6 Uncalled4 Segmentation Commands

```
# preset = splice if RNA and h_sapiens, s_cerevisiae, e_coli, sarscov2
# preset = lr:hq for DNA R10.4.1
# preset = map-ont else
dorado basecaller sup -x cuda:0 --reference <ref.fa> --mm2-opts "-x <preset> --secondary=no" --emit-moves <dataset_r10k.pod5> > <mapped_basecalls.bam>
samtools view -hbF 2304 <mapped_basecalls.bam> > <primary_mapped_basecalls.bam>
uncalled4 align --ref <ref.fa> --reads <dataset_r10k.pod5> --bam-in <primary_mapped_basecalls.bam> --tsv-out <uncalled4_segmentation.tsv> --tsv-cols aln.read_id,dtw --min-aln-length 1
```

S2.2 Normalizing the ONT Signal

All signals are provided in `fast5` or `pod5` (from ONT), or in `slow5` [6] format. Within these formats, they are stored as integer values T_{DACs} to reduce memory space. They can be converted to pA T_{pA} using Equ. 5. The required parameters are also for each read. Each signal is normalized, Equ. 6 and Fig. S2, using the parameters provided by `Dorado`, the state-of-the-art basecaller from ONT. They are found in the `bam` output after basecalling. The shift parameter is stored in the `sm` tag (scaling midpoint) and the scale parameter in the `sd` tag (scaling dispersion). In case of the RNA002 chemistry, `sm` and `sd` are used to normalize the pA signal. For the new RNA004 chemistry, ONT changed the `sm` and `sd` parameter to normalize the raw integer values directly, skipping the conversion to the pA signal.

$$T_{\mathrm{pA}} = \frac{(T_{\mathrm{DACs}} + \mathrm{offset}) * \mathrm{range}}{\mathrm{digitization}} \quad (5)$$

$$T_{\mathrm{norm}} = \frac{T_{\mathrm{pA}} - \mathrm{shift}}{\mathrm{scale}} \quad (6)$$

S2.3 Filtering Outliers

The signal T can contain errors, Fig. S2. They manifest as sudden peaks or drops within the signal. Which is why we apply a Hampel filter [4] to each signal. It removes outliers in time series data, by iterating with a sliding window approach. Within the window, each data point is compared to the window median. When a data point deviates from the median by more than a defined threshold, typically expressed as a multiple of the MAD, it is identified as an outlier. Each outlier is replaced by the window median. The Hampel filter is insensitive to extreme values, making it effective in handling non-Gaussian noise and preserving the integrity of the underlying signal. We use a window size of 6, and a deviation threshold of 5.0.

S2.4 Trimming Adapter

The `bam` file from `Dorado` contains additional tags (https://github.com/nanoporetech/bonito/blob/master/documentation/SAM.md). The `ts` tag holds the number of trimmed signal data points (t) from the start of the signal, e.g. sequencing adapters, Fig. S2. Tag `ns` is the number of signal data points in the signal prior to trimming. During library preparation, multiple reads can get ligated, Fig. S2. Their concatenated signal will be split by `Dorado` into two reads. Split reads get assigned with a new read ID, different from the one of the signal. They have the additional `pi` tag, containing the signal ID and the `sp` tag marking the split position in the signal T. The

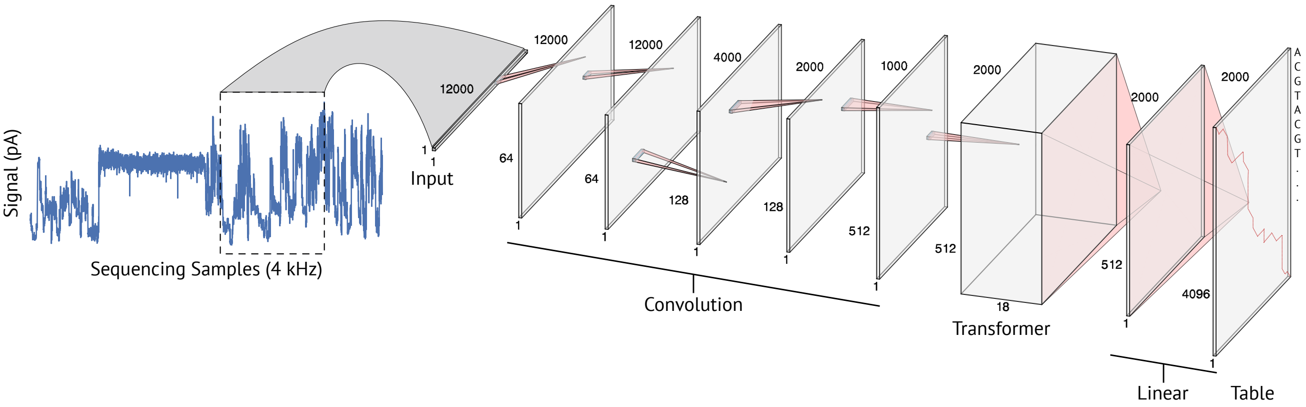

Figure S1. The `Dorado` RNA004 'super accuracy' basecalling model consists of five convolution layers, eighteen transformer layers, and two linear layers. The model outputs a table in the end for each input signal window of 12 000 samples. This table downsamples the 12 000 input signals to 2 000 and holds the models prediction for the nucleotide sequence (rows: 1024 times 4 bases). It can be decoded using algorithms like viterbi or beam search, which will yield the nucleotide sequence for that window. Overlapping nucleotide sequences from overlapping windows are merged later on.

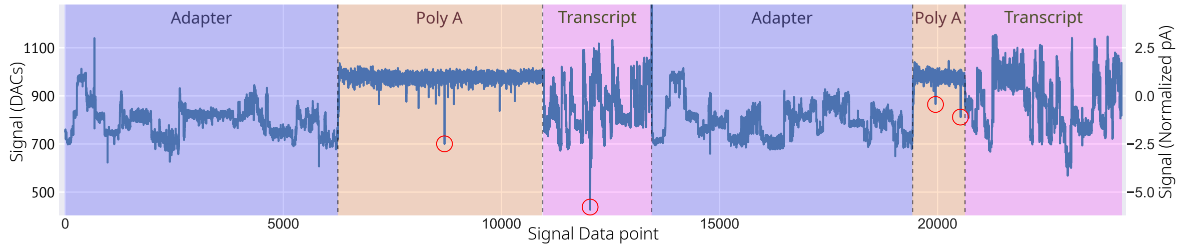

Figure S2. Preprocessing Overview of the Signal. The unnormalized time series (left y-axis) is normalized (right y-axis) to standardize the data. Detected outliers (red circles) mark points that deviate significantly from the expected signal pattern. These outliers can introduce very low probabilities in the HMM Forward-Backward algorithm. The time series presented here originates from a RNA read. It is sequenced from 3' to 5' end, divided into "Adapter," "Poly A," and "Transcript". A DNA signal would be sequenced from 5' to 3' end, contain a different adapter signal, no "Poly A" region, and a part that looks similar to the "Transcript" Within a single signal these segments can appear multiple times, caused by ligated reads or reads, that are sequenced nearly instantaneous after each other. `Dorado` will split these reads during basecalling.

normalized and trimmed signal T_{trimmed}, Equ. 7, and read N is passed to `Dynamont`.

$$T_{\text{trimmed}} = (t_{sp+ts}, \dots, t_{sp+ns-1}) \tag{7}$$

For the sake of simplicity, the trimmed signal T_{trimmed} will be named T with the length $\mathcal{T}$ in the following text.

S2.5 Dynamont with Error Correction

`Dynamont` with error correction aligns N to T while doing the correction on an additional dimension C. It consists of 5 states: A (align), E (extend), I (insertion), S (sequence), and P (polish). A and E are similar to before. A moves in all dimensions, aligning t_i, k_j, and c_h. c_h is a k-mer of C with the index h and k_j is the k-mer of N at position j. c_h and k_j may differ. A starts a new segment for k_j and c_h at the same "time" t_i. E moves in T, while halting in N and C, extending the current segment. I handles the insertion of nucleotides in N, by moving only in N, while halting in T and C. P does the opposite, by moving in T and C it can handle deletions and additional polishings. S moves in N and T, while halting in C. While A allows a simultaneous segment start in all dimensions, P and S allow for different segment starts and ends of the N to T alignment versus the T to C alignment.

The forward algorithm α iterates N and T similar to `NT HMM` but the iteration of C is different. To move from one c to the next, $p(h)$ is introduced. This function returns the indices of preceding k-mers of c_h in C. For example, if c_h = AGGTC then $c_{p(h)}$ are AAGGT, CAGGT, GAGGT, and GAGGT, if Σ ={A, C, G, T}. All rules using $p(h)$ are called $|\Sigma|$ times.

$$
\begin{aligned}
A&: \begin{pmatrix} M_j \\ M_i \\ M_h \end{pmatrix} \to a1 \begin{pmatrix} H_{j-1} \\ M_{i-1} \\ H_{p(h)} \end{pmatrix} \begin{bmatrix} k_j \\ t_i \\ c_h \end{bmatrix} \Bigg| \; a2 \begin{pmatrix} M_{j-1} \\ H_{i-1} \\ H_{p(h)} \end{pmatrix} \begin{bmatrix} k_j \\ t_i \\ c_h \end{bmatrix} \Bigg| \; \begin{bmatrix} \epsilon_{j=0} \\ \epsilon_{i=0} \\ c_h \in C \end{bmatrix} \\
I&: \begin{pmatrix} M_j \\ H_i \\ H_h \end{pmatrix} \to i1 \begin{pmatrix} H_{j-1} \\ M_i \\ H_h \end{pmatrix} \begin{bmatrix} k_j \\ t_i \\ c_h \end{bmatrix} \Bigg| \; i2 \begin{pmatrix} M_{j-1} \\ H_i \\ H_h \end{pmatrix} \begin{bmatrix} k_j \\ t_i \\ c_h \end{bmatrix} \\
P&: \begin{pmatrix} H_j \\ M_i \\ M_h \end{pmatrix} \to p1 \begin{pmatrix} M_j \\ M_{i-1} \\ H_{p(h)} \end{pmatrix} \begin{bmatrix} k_j \\ t_i \\ c_h \end{bmatrix} \Bigg| \; p2 \begin{pmatrix} H_j \\ M_{i-1} \\ H_{p(h)} \end{pmatrix} \begin{bmatrix} k_j \\ t_i \\ c_h \end{bmatrix} \Bigg| \; p3 \begin{pmatrix} M_j \\ H_{i-1} \\ H_{p(h)} \end{pmatrix} \begin{bmatrix} k_j \\ t_i \\ c_h \end{bmatrix} \\
S&: \begin{pmatrix} M_j \\ M_i \\ H_h \end{pmatrix} \to s1 \begin{pmatrix} H_{j-1} \\ M_{i-1} \\ M_h \end{pmatrix} \begin{bmatrix} k_j \\ t_i \\ c_h \end{bmatrix} \Bigg| \; s2 \begin{pmatrix} H_{j-1} \\ M_{i-1} \\ H_h \end{pmatrix} \begin{bmatrix} k_j \\ t_i \\ c_h \end{bmatrix} \Bigg| \; s3 \begin{pmatrix} M_{j-1} \\ H_{i-1} \\ H_h \end{pmatrix} \begin{bmatrix} k_j \\ t_i \\ c_h \end{bmatrix} \\
E&: \begin{pmatrix} H_j \\ M_i \\ H_h \end{pmatrix} \to e1 \begin{pmatrix} M_j \\ M_{i-1} \\ M_h \end{pmatrix} \begin{bmatrix} k_j \\ t_i \\ c_h \end{bmatrix} \Bigg| \; e2 \begin{pmatrix} H_j \\ M_{i-1} \\ M_h \end{pmatrix} \begin{bmatrix} k_j \\ t_i \\ c_h \end{bmatrix} \Bigg| \; e3 \begin{pmatrix} M_j \\ M_{i-1} \\ H_h \end{pmatrix} \begin{bmatrix} k_j \\ t_i \\ c_h \end{bmatrix} \Bigg| \; e4 \begin{pmatrix} H_j \\ M_{i-1} \\ H_h \end{pmatrix} \begin{bmatrix} k_j \\ t_i \\ c_h \end{bmatrix}
\end{aligned}
\tag{8}
$$

As the alignment is not limited in C, the final alignment score Z is not found at a specific index, but as a sum over the last column:

$$Z = \sum_{c_h \in C} E_{\mathcal{N}-k, \mathcal{T}-1, h}$$

The backward algorithm β iterates, backwards over the indices. Here $s(h)$ is the inverse function of $p(h)$. $s(h)$ returns the indices in

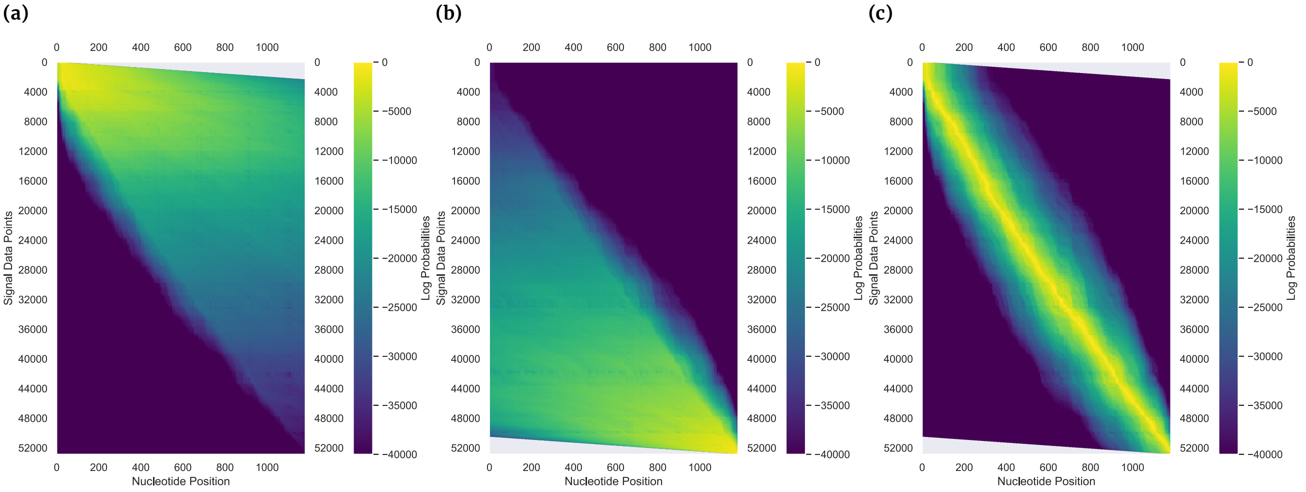

Figure S3. Logarithmic probabilities of full ($N \times T$) `Dynamont` matrices, calculated during a) forward pass; b) backward pass; and c) the combined posterior probability. The matrices show the probability flux of the nucleotide sequence (N) to ONT signal (T) alignment, using the HMM implemented in `Dynamont`. The highest probability can be found around the diagonal, which can be used for further optimizations.

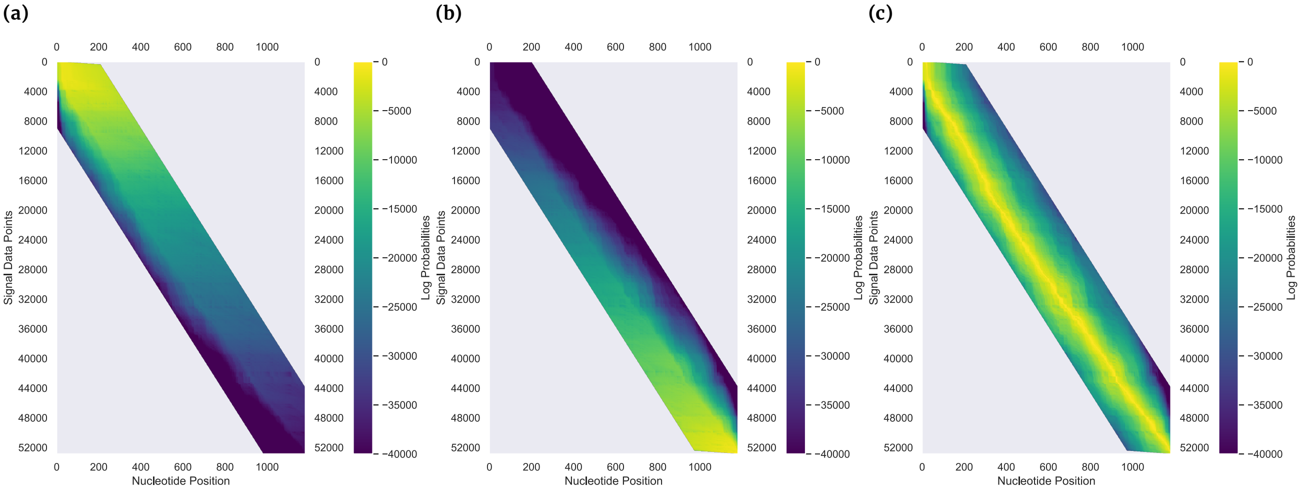

Figure S4. Logarithmic probabilities of banded DP ($N \times T$) `Dynamont` matrices, calculated during a) forward pass; b) backward pass; and c) the combined posterior probability. The matrices show the probability flux of the nucleotide sequence (N) to ONT signal (T) alignment, using the HMM implemented in `Dynamont`. The highest probability can be found around the diagonal, which can be used for further optimizations.

C of the succeeding k-mers of c_h, e.g. if c_h = AGGTC, the succeeding k-mers are GGTCA, GGTCC, GGTCG, and GGTCT.

$$
\begin{aligned}
A^*: \begin{pmatrix} M_j \\ M_i \\ M_h \end{pmatrix} &\longrightarrow e1 \begin{pmatrix} H_j \\ M_{i+1} \\ H_h \end{pmatrix} \begin{bmatrix} k_j \\ t_{i+1} \\ c_h \end{bmatrix} \\
I^*: \begin{pmatrix} M_j \\ H_i \\ H_h \end{pmatrix} &\longrightarrow a2 \begin{pmatrix} M_{j+1} \\ M_{i+1} \\ M_{s(h)} \end{pmatrix} \begin{bmatrix} k_{j+1} \\ t_{i+1} \\ c_{s(h)} \end{bmatrix} \Bigg| \; i2 \begin{pmatrix} M_{j+1} \\ H_i \\ H_h \end{pmatrix} \begin{bmatrix} k_{j+1} \\ t_i \\ c_h \end{bmatrix} \Bigg| \\
&\quad\; p3 \begin{pmatrix} H_j \\ M_{i+1} \\ M_{s(h)} \end{pmatrix} \begin{bmatrix} k_j \\ t_{i+1} \\ c_{s(h)} \end{bmatrix} \Bigg| \; s3 \begin{pmatrix} M_{j+1} \\ M_{i+1} \\ H_h \end{pmatrix} \begin{bmatrix} k_{j+1} \\ t_{i+1} \\ c_h \end{bmatrix} \\
P^*: \begin{pmatrix} H_j \\ M_i \\ M_h \end{pmatrix} &\longrightarrow s1 \begin{pmatrix} M_{j+1} \\ M_{i+1} \\ H_h \end{pmatrix} \begin{bmatrix} k_{j+1} \\ t_{i+1} \\ c_h \end{bmatrix} \Bigg| \; e2 \begin{pmatrix} H_j \\ M_{i+1} \\ H_h \end{pmatrix} \begin{bmatrix} k_j \\ t_{i+1} \\ c_h \end{bmatrix} \\
S^*: \begin{pmatrix} M_j \\ M_i \\ H_h \end{pmatrix} &\longrightarrow p1 \begin{pmatrix} H_j \\ M_{i+1} \\ M_{s(h)} \end{pmatrix} \begin{bmatrix} k_j \\ t_{i+1} \\ c_{s(h)} \end{bmatrix} \Bigg| \; e3 \begin{pmatrix} H_j \\ M_{i+1} \\ H_h \end{pmatrix} \begin{bmatrix} k_j \\ t_{i+1} \\ c_h \end{bmatrix} \\
E^*: \begin{pmatrix} H_j \\ M_i \\ H_h \end{pmatrix} &\longrightarrow a1 \begin{pmatrix} M_{j+1} \\ M_{i+1} \\ M_{s(h)} \end{pmatrix} \begin{bmatrix} k_{j+1} \\ t_{i+1} \\ c_{s(h)} \end{bmatrix} \Bigg| \; i1 \begin{pmatrix} M_{j+1} \\ H_i \\ H_h \end{pmatrix} \begin{bmatrix} k_{j+1} \\ t_i \\ c_h \end{bmatrix} \Bigg| \; p2 \begin{pmatrix} H_j \\ M_{i+1} \\ M_{s(h)} \end{pmatrix} \begin{bmatrix} k_j \\ t_{i+1} \\ c_{s(h)} \end{bmatrix} \Bigg| \\
&\quad\; s2 \begin{pmatrix} M_{j+1} \\ M_{i+1} \\ H_h \end{pmatrix} \begin{bmatrix} k_{j+1} \\ t_{i+1} \\ c_h \end{bmatrix} \Bigg| \; e4 \begin{pmatrix} H_j \\ M_{i+1} \\ H_h \end{pmatrix} \begin{bmatrix} k_j \\ t_{i+1} \\ c_h \end{bmatrix} \Bigg| \Bigg| \begin{bmatrix} \delta^*_{j=\mathcal{N}-k} \\ \delta^*_{i=\mathcal{T}-1} \\ c_h \in C \end{bmatrix}
\end{aligned} \tag{9}
$$

The alignment score Z^* is calculated by:

$$Z^* = \sum_{c_h \in C} A_{0,0,h}$$

Analogous to `Dynamont`s basic algorithm, the runtime and memory complexity for the error correction is $O(\mathcal{B}\mathcal{T}|C|)$.

S2.6 Training Parameters with the Baum-Welch Algorithm

Using the Forward-Backward algorithm, we can utilize the Baum-Welch algorithm [5, 1] to train our parameters. It is a special case of the Expectation Maximization Algorithm to find unknown transition and emission parameters of an HMM.

S2.6.1 Training transition parameters

The transition parameters are trained by calculating how many times they were used given a training pair of T and N. Given the

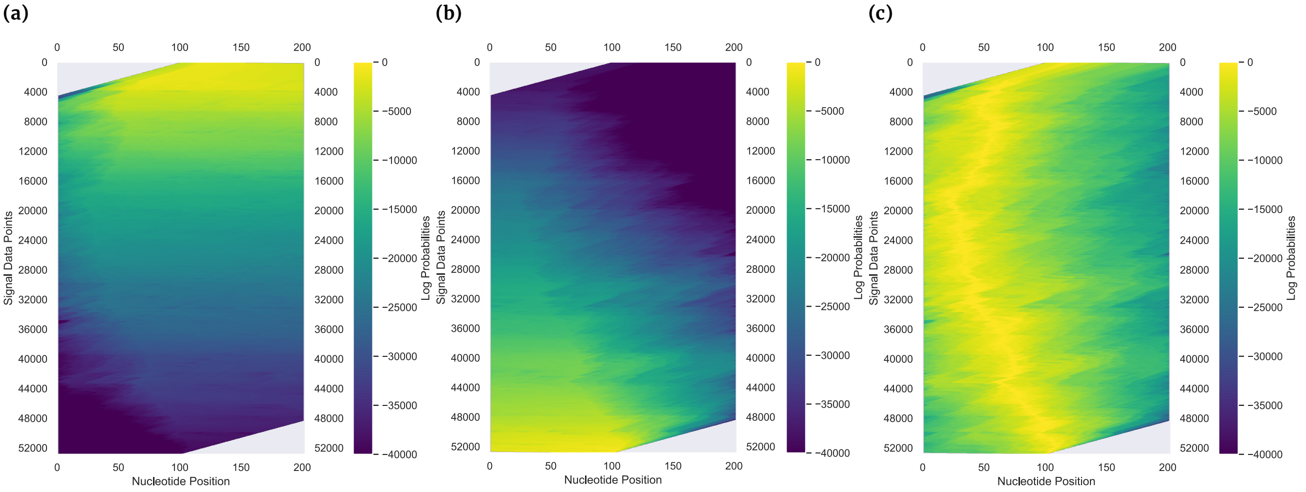

Figure S5. Logarithmic probabilities of reduced banded DP ($N \times T$) `Dynamont` matrices, calculated during a) forward pass; b) backward pass; and c) the combined posterior probability. The matrices show the probability flux of the nucleotide sequence (N) to ONT signal (T) alignment, using the HMM implemented in `Dynamont`. The highest probability can be found around the diagonal, which can be used for further optimizations.

Table S1. Metrics for *H. sapiens* RNA002 data set.

Tool	Dorado	Tombo	f5c R.	f5c E.	Uncalled4	Dynamont
median delta ($\Delta\mu$)	0.240 $_{18.5}$	0.740 $_{57.0}$	0.916 $_{70.5}$	0.961 $_{74.0}$	1.000 $_{77.0}$	0.929 $_{71.5}$
mad delta ($\Delta\sigma$)	0.875 $_{7.0}$	0.938 $_{7.5}$	0.750 $_{6.0}$	0.750 $_{6.0}$	0.750 $_{6.0}$	1.000 $_{8.0}$
homogeneity	0.000 $_{17.0}$	0.118 $_{15.0}$	0.176 $_{14.0}$	0.176 $_{14.0}$	0.176 $_{14.0}$	0.118 $_{15.0}$
segmented reads	1.000 $_{10946.0}$	0.666 $_{7294.0}$	0.705 $_{7713.0}$	0.629 $_{6883.0}$	0.752 $_{8232.0}$	1.000 $_{10946.0}$
truncated reads	1.000 $_{0.0}$	0.132 $_{7020.0}$	0.046 $_{7713.0}$	0.151 $_{6865.0}$	0.000 $_{8087.0}$	0.905 $_{771.0}$
min read length	0.975 $_{5.0}$	0.770 $_{46.0}$	0.000 $_{200.0}$	0.625 $_{75.0}$	0.625 $_{75.0}$	0.975 $_{5.0}$
n50 read length	1.000 $_{1122.0}$	0.730 $_{819.0}$	0.799 $_{896.0}$	0.705 $_{791.0}$	0.707 $_{793.0}$	0.773 $_{867.0}$
max read length	1.000 $_{21695.0}$	0.222 $_{4819.0}$	0.224 $_{4857.0}$	0.214 $_{4653.0}$	0.222 $_{4811.0}$	0.248 $_{5387.0}$
flye total length	0.998 $_{11983.0}$	0.777 $_{9330.0}$	0.970 $_{11644.0}$	0.394 $_{4732.0}$	0.966 $_{11589.0}$	1.000 $_{12002.0}$
flye n50	0.956 $_{2341.0}$	0.939 $_{2299.0}$	0.951 $_{2328.0}$	1.000 $_{2449.0}$	0.931 $_{2281.0}$	0.959 $_{2349.0}$
flye mean coverage	1.000 $_{5.8}$	0.991 $_{5.8}$	1.000 $_{5.8}$	0.948 $_{5.5}$	0.897 $_{5.2}$	1.000 $_{5.8}$
svim structural variants	0.000 $_{0.0}$	0.000 $_{0.0}$	1.000 $_{245.0}$	0.188 $_{46.0}$	0.004 $_{1.0}$	0.135 $_{33.0}$
AM-score	9.04	7.02	7.54	6.74	7.03	9.04

states q and r, a_{qr} is the transition probability from q to r. The new transition $\hat{a}_{qr}$ is calculated with Equ. 10.

$$A_{qr} = \frac{\sum_x \alpha_q(x) a_{qr} \phi(o_{x+1}) \beta_r(x+1)}{Z}$$
$$\hat{a}_{qr} = \frac{A_{qr}}{\sum_{r'} A_{qr'}} \tag{10}$$

$\sum_x$ iterates the observations o, in our case k_j and t_i. $\alpha_q(x)$ is the score in state q in α at x. $\beta_r(x+1)$ is the score of state r in β when taking a_{qr} to $x+1$. a_{qr} is the transition probability for the path from $\alpha_q(x)$ to $\beta_r(x+1)$. $\phi(o_{x+1})$ is the emission for observing o_{x+1}. Each transition probability needs to be normalized afterwards to ensure that all outgoing transitions sum up to 1.

S2.6.2 *Training emission parameters*

New model parameters $\hat{\mu}_k$ and $\hat{\sigma}_k$ for the Gaussian distributions are trained with the Baum-Welch algorithm [2, 3]. First, the expectation step calculates weights for all observations, Equ. 11. For each i and j, the sum of scores of all matrices Q in α get multiplied with the sum of scores of all matrices Q^* in β, which is standardized by Z. γ is a matrix that stores these weights for all i and j.

$$\gamma_{i,j} = \frac{\sum_Q Q_{i,j} \sum_{Q^*} Q^*_{i,j}}{Z} \tag{11}$$

Within the maximization step, Equ. 12, the weights in γ are used to calculate a weighted sum of the observations t_i, which will be standardized with the sum of weights over i of γ, to get $\hat{\mu}_j$. As a k-mer can appear multiple times in N, $\hat{\mu}_k$ is calculated by taking the average $\hat{\mu}_j$ for all $k_j = k$ in N. The same happens for $\hat{\sigma}_k$.

$$\hat{\mu}_j = \frac{\sum_{i=1}^{\mathcal{T}-1} \gamma_{i,j} t_i}{\sum_{i=1}^{\mathcal{T}-1} \gamma_{i,j}} \qquad \hat{\sigma}_j^2 = \frac{\sum_{i=1}^{\mathcal{T}-1} \gamma_{i,j} (t_i - \hat{\mu}_j)^2}{\sum_{i=1}^{\mathcal{T}-1} \gamma_{i,j}}$$
$$\hat{\mu}_k = \frac{\sum_{\forall j: k_j = k} \hat{\mu}_j}{\text{counts}_k(N)} \qquad \hat{\sigma}_k = \sqrt{\frac{\sum_{\forall j: k_j = k} \hat{\sigma}_j^2}{\text{counts}_k(N)}} \tag{12}$$

Table S2. Metrics for *E. coli* RNA002 data set.

Tool	Dorado	Tombo	f5c R.	f5c E.	Uncalled4	Dynamont
median delta (Δμ)	$0.248_{19.5}$	$0.783_{61.5}$	$0.866_{68.0}$	$0.936_{73.5}$	$1.000_{78.5}$	$0.955_{75.0}$
mad delta (Δσ)	$0.882_{7.5}$	$0.941_{8.0}$	$0.706_{6.0}$	$0.765_{6.5}$	$0.765_{6.5}$	$1.000_{8.5}$
homogeneity	$0.000_{18.0}$	$0.139_{15.5}$	$0.222_{14.0}$	$0.167_{15.0}$	$0.167_{15.0}$	$0.167_{15.0}$
segmented reads	$1.000_{10717.0}$	$0.170_{1821.0}$	$0.242_{2593.0}$	$0.145_{1559.0}$	$0.183_{1959.0}$	$1.000_{10717.0}$
truncated reads	$1.000_{0.0}$	$0.324_{1754.0}$	$0.000_{2593.0}$	$0.399_{1558.0}$	$0.253_{1938.0}$	$0.953_{121.0}$
min read length	$0.975_{5.0}$	$0.780_{44.0}$	$0.000_{200.0}$	$0.700_{60.0}$	$0.640_{72.0}$	$0.950_{10.0}$
n50 read length	$0.643_{397.0}$	$0.765_{472.0}$	$1.000_{617.0}$	$0.900_{555.0}$	$0.754_{465.0}$	$0.624_{385.0}$
max read length	$1.000_{31807.0}$	$0.075_{2372.0}$	$0.099_{3163.0}$	$0.084_{2675.0}$	$0.074_{2364.0}$	$1.000_{31812.0}$
flye total length	$0.918_{5093.0}$	$0.000_{0.0}$	$1.000_{5550.0}$	$0.000_{0.0}$	$0.000_{0.0}$	$0.919_{5098.0}$
flye n50	$0.912_{2648.0}$	$0.000_{0.0}$	$1.000_{2903.0}$	$0.000_{0.0}$	$0.000_{0.0}$	$0.914_{2653.0}$
flye mean coverage	$1.000_{17.0}$	$0.000_{0.0}$	$0.647_{11.0}$	$0.000_{0.0}$	$0.000_{0.0}$	$0.971_{16.5}$
svim structural variants	$0.331_{44.0}$	$0.000_{0.0}$	$0.000_{0.0}$	$0.045_{6.0}$	$0.060_{8.0}$	$1.000_{133.0}$
aggregated metric score	8.91	3.98	5.78	4.14	3.89	10.45

Table S3. Metrics for SARS-CoV-2 RNA002 data set.

Tool	Dorado	Tombo	f5c R.	f5c E.	Uncalled4	Dynamont
median delta (Δμ)	$0.248_{18.5}$	$0.718_{53.5}$	$0.906_{67.5}$	$0.899_{67.0}$	$1.000_{74.5}$	$0.960_{71.5}$
mad delta (Δσ)	$0.933_{7.0}$	$1.000_{7.5}$	$0.733_{5.5}$	$0.800_{6.0}$	$0.800_{6.0}$	$1.000_{7.5}$
homogeneity	$0.000_{16.5}$	$0.152_{14.0}$	$0.212_{13.0}$	$0.182_{13.5}$	$0.152_{14.0}$	$0.152_{14.0}$
segmented reads	$1.000_{10569.0}$	$0.548_{5788.0}$	$0.845_{8935.0}$	$0.541_{5718.0}$	$0.599_{6333.0}$	$1.000_{10569.0}$
truncated reads	$1.000_{0.0}$	$0.353_{5777.0}$	$0.000_{8935.0}$	$0.360_{5718.0}$	$0.293_{6315.0}$	$0.898_{910.0}$
min read length	$0.975_{5.0}$	$0.721_{56.0}$	$0.000_{201.0}$	$0.607_{79.0}$	$0.592_{82.0}$	$0.950_{10.0}$
n50 read length	$0.991_{1957.0}$	$0.632_{1248.0}$	$1.000_{1974.0}$	$0.880_{1737.0}$	$0.630_{1243.0}$	$0.978_{1930.0}$
max read length	$1.000_{11296.0}$	$0.525_{5935.0}$	$0.995_{11246.0}$	$0.802_{9062.0}$	$0.524_{5927.0}$	$1.000_{11301.0}$
flye total length	$0.932_{35529.0}$	$0.312_{11879.0}$	$0.952_{36284.0}$	$0.032_{1232.0}$	$0.256_{9774.0}$	$1.000_{38108.0}$
flye n50	$0.999_{8919.0}$	$0.903_{8062.0}$	$0.997_{8905.0}$	$0.138_{1232.0}$	$0.810_{7233.0}$	$1.000_{8929.0}$
flye mean coverage	$0.006_{23.2}$	$0.008_{33.5}$	$0.005_{21.9}$	$1.000_{4171.0}$	$0.011_{44.5}$	$0.008_{35.2}$
svim structural variants	$0.027_{3.0}$	$0.000_{0.0}$	$0.000_{0.0}$	$0.118_{13.0}$	$0.036_{4.0}$	$1.000_{110.0}$
AM-score	8.11	5.87	6.65	6.36	5.70	9.95

Table S4. Metrics for IVT RNA002 data set.

Tool	Dorado	Tombo	f5c R.	f5c E.	Uncalled4	Dynamont
median delta (Δμ)	$0.247_{18.5}$	$0.773_{58.0}$	$0.873_{65.5}$	$0.947_{71.0}$	$1.000_{75.0}$	$0.953_{71.5}$
mad delta (Δσ)	$0.875_{7.0}$	$0.938_{7.5}$	$0.688_{5.5}$	$0.750_{6.0}$	$0.750_{6.0}$	$1.000_{8.0}$
homogeneity	$0.000_{17.0}$	$0.118_{15.0}$	$0.206_{13.5}$	$0.176_{14.0}$	$0.176_{14.0}$	$0.118_{15.0}$
segmented reads	$1.000_{10946.0}$	$0.666_{7294.0}$	$0.705_{7713.0}$	$0.629_{6883.0}$	$0.752_{8232.0}$	$1.000_{10946.0}$
truncated reads	$1.000_{0.0}$	$0.132_{7020.0}$	$0.046_{7713.0}$	$0.151_{6865.0}$	$0.000_{8087.0}$	$0.905_{771.0}$
min read length	$0.975_{5.0}$	$0.770_{46.0}$	$0.000_{200.0}$	$0.625_{75.0}$	$0.625_{75.0}$	$0.975_{5.0}$
n50 read length	$1.000_{988.0}$	$0.829_{819.0}$	$0.907_{896.0}$	$0.801_{791.0}$	$0.803_{793.0}$	$0.878_{867.0}$
max read length	$0.848_{4566.0}$	$0.895_{4819.0}$	$0.902_{4857.0}$	$0.864_{4653.0}$	$0.893_{4811.0}$	$1.000_{5387.0}$
flye total length	$0.998_{11983.0}$	$0.777_{9330.0}$	$0.970_{11644.0}$	$0.394_{4732.0}$	$0.966_{11589.0}$	$1.000_{12002.0}$
flye n50	$0.956_{2341.0}$	$0.939_{2299.0}$	$0.951_{2328.0}$	$1.000_{2449.0}$	$0.931_{2281.0}$	$0.959_{2349.0}$
flye mean coverage	$1.000_{5.8}$	$0.991_{5.8}$	$1.000_{5.8}$	$0.948_{5.5}$	$0.897_{5.2}$	$1.000_{5.8}$
svim structural variants	$0.000_{0.0}$	$0.000_{0.0}$	$1.000_{245.0}$	$0.188_{46.0}$	$0.004_{1.0}$	$0.135_{33.0}$
AM-score	8.90	7.83	8.25	7.47	7.80	9.92

Table S5. Metrics for IVT *H. sapiens* RNA002 data set.

Tool	Dorado	Tombo	f5c R.	f5c E.	Uncalled4	Dynamont
median delta ($\Delta\mu$)	0.260 19.5	0.740 55.5	0.893 67.0	0.940 70.5	1.000 75.0	0.953 71.5
mad delta ($\Delta\sigma$)	0.875 7.0	0.938 7.5	0.750 6.0	0.750 6.0	0.750 6.0	1.000 8.0
homogeneity	0.000 17.5	0.143 15.0	0.200 14.0	0.171 14.5	0.171 14.5	0.143 15.0
segmented reads	1.000 10946.0	0.666 7294.0	0.705 7713.0	0.629 6883.0	0.752 8232.0	1.000 10946.0
truncated reads	1.000 0.0	0.132 7020.0	0.046 7713.0	0.151 6865.0	0.000 8087.0	0.905 771.0
min read length	0.975 5.0	0.770 46.0	0.000 200.0	0.625 75.0	0.625 75.0	0.975 5.0
n50 read length	0.980 878.0	0.914 819.0	1.000 896.0	0.883 791.0	0.885 793.0	0.968 867.0
max read length	0.956 5148.0	0.895 4819.0	0.902 4857.0	0.864 4653.0	0.893 4811.0	1.000 5387.0
flye total length	0.998 11983.0	0.777 9330.0	0.970 11644.0	0.394 4732.0	0.966 11589.0	1.000 12002.0
flye n50	0.956 2341.0	0.939 2299.0	0.951 2328.0	1.000 2449.0	0.931 2281.0	0.959 2349.0
flye mean coverage	1.000 5.8	0.991 5.8	1.000 5.8	0.948 5.5	0.897 5.2	1.000 5.8
svim structural variants	0.000 0.0	0.000 0.0	1.000 245.0	0.188 46.0	0.004 1.0	0.135 33.0
AM-score	9.00	7.90	8.42	7.54	7.87	10.04

Table S6. Metrics for m$^1\Psi$ RNA002 data set.

Tool	Dorado	Tombo	f5c R.	f5c E.	Uncalled4	Dynamont
median delta ($\Delta\mu$)	0.268 20.0	0.644 48.0	0.940 70.0	1.000 74.5	0.993 74.0	0.966 72.0
mad delta ($\Delta\sigma$)	0.789 7.5	0.789 7.5	0.632 6.0	0.737 7.0	0.684 6.5	1.000 9.5
homogeneity	0.000 17.0	0.088 15.5	0.176 14.0	0.059 16.0	0.118 15.0	0.029 16.5
segmented reads	1.000 10151.0	0.082 836.0	0.875 8878.0	0.082 831.0	0.084 854.0	1.000 10151.0
truncated reads	1.000 0.0	0.906 834.0	0.000 8878.0	0.906 831.0	0.904 854.0	0.991 79.0
min read length	0.960 8.0	0.785 43.0	0.000 200.0	0.810 38.0	0.825 35.0	0.950 10.0
n50 read length	0.999 2111.0	0.522 1104.0	0.989 2090.0	0.440 931.0	0.520 1099.0	1.000 2114.0
max read length	1.000 10245.0	0.212 2173.0	0.983 10075.0	0.188 1929.0	0.211 2165.0	1.000 10250.0
flye total length	0.000 0.0	0.000 0.0	0.000 0.0	0.000 0.0	0.000 0.0	0.000 0.0
flye n50	0.000 0.0	0.000 0.0	0.000 0.0	0.000 0.0	0.000 0.0	0.000 0.0
flye mean coverage	0.000 0.0	0.000 0.0	0.000 0.0	0.000 0.0	0.000 0.0	0.000 0.0
svim structural variants	0.210 33.0	0.000 0.0	0.000 0.0	1.000 157.0	0.000 0.0	0.019 3.0
AM-score	6.23	4.03	4.59	5.22	4.34	6.96

Table S7. Metrics for *S. cerevisiae* RNA004 data set.

Tool	Dorado	f5c R.	f5c E.	Uncalled4	Dynamont
median delta ($\Delta\mu$)	0.250 13.5	0.935 50.5	0.944 51.0	0.935 50.5	1.000 54.0
mad delta ($\Delta\sigma$)	0.688 5.5	0.562 4.5	0.625 5.0	0.625 5.0	1.000 8.0
homogeneity	0.000 14.0	0.214 11.0	0.179 11.5	0.179 11.5	0.000 14.0
segmented reads	1.000 10289.0	0.309 3178.0	0.274 2815.0	0.784 8067.0	1.000 10288.0
truncated reads	1.000 0.0	0.606 3178.0	0.651 2813.0	0.000 8064.0	0.696 2448.0
min read length	0.975 5.0	0.000 200.0	0.700 60.0	0.670 66.0	0.985 3.0
n50 read length	0.890 1377.0	1.000 1548.0	0.262 405.0	0.077 119.0	0.846 1310.0
max read length	1.000 114273.0	0.050 5768.0	0.039 4470.0	0.040 4624.0	0.601 68641.0
flye total length	0.827 4177.0	1.000 5050.0	0.000 0.0	0.000 0.0	0.870 4393.0
flye n50	0.827 4177.0	1.000 5050.0	0.000 0.0	0.000 0.0	0.870 4393.0
flye mean coverage	0.973 852.0	0.825 723.0	0.000 0.0	0.000 0.0	1.000 876.0
svim structural variants	0.000 0.0	0.000 0.0	0.000 0.0	0.000 0.0	0.000 0.0
AM-score	8.43	6.50	3.67	3.31	8.87

Table S8. Metrics for CEVd RNA004 data set.

Tool	Dorado	f5c R.	f5c E.	Uncalled4	Dynamont
median delta ($\Delta\mu$)	$0.257_{13.5}$	$0.924_{48.5}$	$1.000_{52.5}$	$0.981_{51.5}$	$0.981_{51.5}$
mad delta ($\Delta\sigma$)	$0.846_{5.5}$	$0.692_{4.5}$	$0.769_{5.0}$	$0.846_{5.5}$	$1.000_{6.5}$
homogeneity	$0.000_{14.0}$	$0.214_{11.0}$	$0.143_{12.0}$	$0.107_{12.5}$	$0.071_{13.0}$
segmented reads	$1.000_{10058.0}$	$0.162_{1634.0}$	$0.151_{1519.0}$	$0.682_{6864.0}$	$1.000_{10058.0}$
truncated reads	$1.000_{0.0}$	$0.761_{1634.0}$	$0.778_{1517.0}$	$0.000_{6832.0}$	$0.302_{4768.0}$
min read length	$0.965_{7.0}$	$0.000_{200.0}$	$0.785_{43.0}$	$0.865_{27.0}$	$0.985_{3.0}$
n50 read length	$0.619_{190.0}$	$1.000_{307.0}$	$0.769_{236.0}$	$0.531_{163.0}$	$0.609_{187.0}$
max read length	$0.966_{12887.0}$	$0.147_{1963.0}$	$0.046_{620.0}$	$0.040_{537.0}$	$1.000_{13345.0}$
flye total length	$0.000_{0.0}$	$0.000_{0.0}$	$0.000_{0.0}$	$0.000_{0.0}$	$0.000_{0.0}$
flye n50	$0.000_{0.0}$	$0.000_{0.0}$	$0.000_{0.0}$	$0.000_{0.0}$	$0.000_{0.0}$
flye mean coverage	$0.000_{0.0}$	$0.000_{0.0}$	$0.000_{0.0}$	$0.000_{0.0}$	$0.000_{0.0}$
svim structural variants	$1.000_{143.0}$	$0.014_{2.0}$	$0.028_{4.0}$	$0.000_{0.0}$	$0.028_{4.0}$
AM-score	6.65	3.91	4.47	4.05	5.98

Table S9. Metrics for IVT RNA004 data set.

Tool	Dorado	f5c R.	f5c E.	Uncalled4	Dynamont
median delta ($\Delta\mu$)	$0.231_{12.0}$	$0.798_{41.5}$	$1.000_{52.0}$	$0.981_{51.0}$	$0.827_{43.0}$
mad delta ($\Delta\sigma$)	$0.833_{5.0}$	$0.750_{4.5}$	$0.833_{5.0}$	$0.917_{5.5}$	$1.000_{6.0}$
homogeneity	$0.000_{13.0}$	$0.154_{11.0}$	$0.077_{12.0}$	$0.038_{12.5}$	$0.000_{13.0}$
segmented reads	$1.000_{10136.0}$	$0.280_{2838.0}$	$0.269_{2730.0}$	$0.669_{6777.0}$	$1.000_{10136.0}$
truncated reads	$1.000_{0.0}$	$0.580_{2838.0}$	$0.596_{2729.0}$	$0.000_{6750.0}$	$0.365_{4284.0}$
min read length	$0.975_{5.0}$	$0.000_{200.0}$	$0.775_{45.0}$	$0.845_{31.0}$	$0.990_{2.0}$
n50 read length	$0.776_{257.0}$	$1.000_{331.0}$	$0.779_{258.0}$	$0.625_{207.0}$	$0.779_{258.0}$
max read length	$1.000_{27894.0}$	$0.977_{27265.0}$	$0.030_{843.0}$	$0.022_{611.0}$	$0.983_{27406.0}$
flye total length	$0.792_{604.0}$	$0.000_{0.0}$	$0.000_{0.0}$	$0.000_{0.0}$	$1.000_{763.0}$
flye n50	$0.973_{604.0}$	$0.000_{0.0}$	$0.000_{0.0}$	$0.000_{0.0}$	$1.000_{621.0}$
flye mean coverage	$0.151_{20.0}$	$0.000_{0.0}$	$0.000_{0.0}$	$0.000_{0.0}$	$1.000_{132.5}$
svim structural variants	$0.000_{0.0}$	$0.000_{0.0}$	$1.000_{208.0}$	$0.000_{0.0}$	$0.043_{9.0}$
AM-score	7.73	4.54	5.36	4.10	8.99

Table S10. Metrics for Ψ RNA004 data set.

Tool	Dorado	f5c R.	f5c E.	Uncalled4	Dynamont
median delta ($\Delta\mu$)	$0.173_{22.0}$	$1.000_{127.0}$	$0.423_{53.8}$	$0.429_{54.5}$	$0.935_{118.8}$
mad delta ($\Delta\sigma$)	$0.714_{10.0}$	$0.714_{10.0}$	$0.429_{6.0}$	$0.464_{6.5}$	$1.000_{14.0}$
homogeneity	$0.000_{25.0}$	$0.240_{19.0}$	$0.440_{14.0}$	$0.440_{14.0}$	$0.200_{20.0}$
segmented reads	$1.000_{10029.0}$	$0.004_{45.0}$	$0.001_{9.0}$	$0.634_{6358.0}$	$1.000_{10029.0}$
truncated reads	$1.000_{0.0}$	$0.993_{45.0}$	$0.999_{9.0}$	$0.000_{6357.0}$	$0.711_{1836.0}$
min read length	$0.975_{5.0}$	$0.000_{200.0}$	$0.920_{16.0}$	$0.870_{26.0}$	$0.990_{2.0}$
n50 read length	$0.053_{89.0}$	$1.000_{1669.0}$	$0.043_{72.0}$	$0.043_{72.0}$	$0.053_{88.0}$
max read length	$1.000_{10264.0}$	$0.999_{10254.0}$	$0.013_{129.0}$	$0.008_{84.0}$	$1.000_{10265.0}$
flye total length	$1.000_{622.0}$	$0.000_{0.0}$	$0.000_{0.0}$	$0.000_{0.0}$	$0.894_{556.0}$
flye n50	$1.000_{622.0}$	$0.000_{0.0}$	$0.000_{0.0}$	$0.000_{0.0}$	$0.894_{556.0}$
flye mean coverage	$1.000_{20.0}$	$0.000_{0.0}$	$0.000_{0.0}$	$0.000_{0.0}$	$1.000_{20.0}$
svim structural variants	$1.000_{15.0}$	$0.000_{0.0}$	$0.267_{4.0}$	$0.000_{0.0}$	$0.000_{0.0}$
AM-score	8.92	4.95	3.53	2.89	8.68

Table S11. Metrics for *H. sapiens* DNA data set.

Tool	Dorado	f5c R.	f5c E.	Uncalled4	Dynamont
median delta (Δμ)	0.267 $_{27.0}$	0.812 $_{82.0}$	0.817 $_{82.5}$	0.822 $_{83.0}$	1.000 $_{101.0}$
mad delta (Δσ)	0.812 $_{6.5}$	0.750 $_{6.0}$	0.750 $_{6.0}$	0.688 $_{5.5}$	1.000 $_{8.0}$
homogeneity	0.000 $_{12.0}$	0.333 $_{8.0}$	0.333 $_{8.0}$	0.333 $_{8.0}$	0.208 $_{9.5}$
segmented reads	1.000 $_{10067.0}$	0.955 $_{9617.0}$	0.941 $_{9477.0}$	0.982 $_{9887.0}$	0.993 $_{9996.0}$
truncated reads	1.000 $_{0.0}$	0.038 $_{9617.0}$	0.056 $_{9436.0}$	0.125 $_{8750.0}$	0.000 $_{9996.0}$
min read length	0.493 $_{102.0}$	0.000 $_{201.0}$	0.507 $_{99.0}$	0.493 $_{102.0}$	0.826 $_{35.0}$
n50 read length	1.000 $_{22168.0}$	0.987 $_{21876.0}$	0.917 $_{20320.0}$	0.949 $_{21039.0}$	0.943 $_{20907.0}$
max read length	1.000 $_{253314.0}$	0.375 $_{95050.0}$	0.366 $_{92838.0}$	0.242 $_{61347.0}$	0.369 $_{93590.0}$
flye total length	0.643 $_{165948.0}$	0.762 $_{196434.0}$	1.000 $_{257938.0}$	0.539 $_{138997.0}$	0.624 $_{161069.0}$
flye n50	0.318 $_{8147.0}$	0.476 $_{12177.0}$	1.000 $_{25582.0}$	0.613 $_{15687.0}$	0.403 $_{10302.0}$
flye mean coverage	0.566 $_{61.0}$	0.428 $_{46.2}$	1.000 $_{107.9}$	0.694 $_{74.9}$	0.409 $_{44.2}$
svim structural variants	1.000 $_{1112.0}$	0.000 $_{0.0}$	0.000 $_{0.0}$	0.000 $_{0.0}$	0.000 $_{0.0}$
AM-score	8.10	5.92	7.69	6.48	6.78

Table S12. Metrics for Zymo HMW DNA data set.

Tool	Dorado	f5c R.	f5c E.	Uncalled4	Dynamont
median delta (Δμ)	0.303 $_{23.0}$	0.987 $_{75.0}$	0.987 $_{75.0}$	1.000 $_{76.0}$	0.197 $_{15.0}$
mad delta (Δσ)	1.000 $_{5.5}$	0.909 $_{5.0}$	0.818 $_{4.5}$	0.818 $_{4.5}$	0.818 $_{4.5}$
homogeneity	0.562 $_{10.5}$	0.708 $_{7.0}$	0.708 $_{7.0}$	0.708 $_{7.0}$	0.000 $_{24.0}$
segmented reads	1.000 $_{10095.0}$	0.970 $_{9789.0}$	0.961 $_{9702.0}$	0.989 $_{9989.0}$	1.000 $_{10092.0}$
truncated reads	1.000 $_{0.0}$	0.030 $_{9789.0}$	0.039 $_{9699.0}$	0.116 $_{8917.0}$	0.000 $_{10092.0}$
min read length	0.951 $_{32.0}$	0.000 $_{647.0}$	0.304 $_{450.0}$	0.626 $_{242.0}$	0.998 $_{1.0}$
n50 read length	1.000 $_{13092.0}$	0.979 $_{12816.0}$	0.945 $_{12372.0}$	0.954 $_{12494.0}$	0.992 $_{12984.0}$
max read length	1.000 $_{104946.0}$	0.999 $_{104814.0}$	0.966 $_{101384.0}$	0.802 $_{84175.0}$	1.000 $_{104938.0}$
flye total length	0.985 $_{9829547.0}$	0.862 $_{8606597.0}$	0.899 $_{8970220.0}$	1.000 $_{9978966.0}$	0.926 $_{9239751.0}$
flye n50	1.000 $_{77313.0}$	0.991 $_{76623.0}$	0.740 $_{57190.0}$	0.939 $_{72620.0}$	0.991 $_{76618.0}$
flye mean coverage	0.936 $_{4.3}$	0.985 $_{4.6}$	0.906 $_{4.2}$	0.927 $_{4.3}$	1.000 $_{4.6}$
svim structural variants	1.000 $_{552.0}$	0.281 $_{155.0}$	0.000 $_{0.0}$	0.000 $_{0.0}$	0.087 $_{48.0}$
AM-score	10.74	8.70	8.27	8.88	8.01

Table S13. Metrics for *S. aureus* DNA data set.

Tool	Dorado	f5c R.	f5c E.	Uncalled4	Dynamont
median delta (Δμ)	0.263 $_{25.0}$	0.858 $_{81.5}$	0.858 $_{81.5}$	0.863 $_{82.0}$	1.000 $_{95.0}$
mad delta (Δσ)	0.923 $_{6.0}$	0.769 $_{5.0}$	0.769 $_{5.0}$	0.769 $_{5.0}$	1.000 $_{6.5}$
homogeneity	0.000 $_{11.0}$	0.364 $_{7.0}$	0.364 $_{7.0}$	0.364 $_{7.0}$	0.273 $_{8.0}$
segmented reads	1.000 $_{10044.0}$	0.985 $_{9892.0}$	0.951 $_{9556.0}$	0.966 $_{9699.0}$	1.000 $_{10044.0}$
truncated reads	1.000 $_{0.0}$	0.015 $_{9892.0}$	0.049 $_{9553.0}$	0.036 $_{9687.0}$	0.000 $_{10044.0}$
min read length	0.563 $_{90.0}$	0.000 $_{206.0}$	0.684 $_{65.0}$	0.665 $_{69.0}$	0.927 $_{15.0}$
n50 read length	0.998 $_{7717.0}$	0.990 $_{7659.0}$	0.737 $_{5704.0}$	0.912 $_{7057.0}$	1.000 $_{7735.0}$
max read length	1.000 $_{109657.0}$	1.000 $_{109644.0}$	0.565 $_{61954.0}$	0.861 $_{94377.0}$	1.000 $_{109649.0}$
flye total length	1.000 $_{3106880.0}$	0.971 $_{3017648.0}$	0.811 $_{2519367.0}$	0.847 $_{2630217.0}$	1.000 $_{3106909.0}$
flye n50	1.000 $_{2257641.0}$	0.961 $_{2170229.0}$	0.026 $_{59661.0}$	0.108 $_{242939.0}$	1.000 $_{2257665.0}$
flye mean coverage	0.787 $_{12.8}$	1.000 $_{16.2}$	0.735 $_{11.9}$	0.770 $_{12.5}$	0.787 $_{12.8}$
svim structural variants	1.000 $_{439.0}$	0.141 $_{62.0}$	0.000 $_{0.0}$	0.023 $_{10.0}$	0.000 $_{0.0}$
AM-score	9.53	8.05	6.55	7.18	8.99

Table S14. Metrics for *P. anserina* DNA data set.

Tool	Dorado	f5c R.	f5c E.	Uncalled4	Dynamont
median delta ($\Delta\mu$)	$0.280_{24.5}$	$0.851_{74.5}$	$0.857_{75.0}$	$0.869_{76.0}$	$1.000_{87.5}$
mad delta ($\Delta\sigma$)	$0.917_{5.5}$	$0.833_{5.0}$	$0.833_{5.0}$	$0.833_{5.0}$	$1.000_{6.0}$
homogeneity	$0.000_{10.5}$	$0.333_{7.0}$	$0.333_{7.0}$	$0.333_{7.0}$	$0.286_{7.5}$
segmented reads	$1.000_{10045.0}$	$0.965_{9693.0}$	$0.965_{9693.0}$	$0.998_{10028.0}$	$1.000_{10045.0}$
truncated reads	$1.000_{0.0}$	$0.035_{9693.0}$	$0.036_{9683.0}$	$0.003_{10011.0}$	$0.000_{10045.0}$
min read length	$0.463_{124.0}$	$0.000_{231.0}$	$0.632_{85.0}$	$0.597_{93.0}$	$0.922_{18.0}$
n50 read length	$1.000_{5560.0}$	$0.985_{5474.0}$	$0.924_{5137.0}$	$0.930_{5171.0}$	$0.999_{5553.0}$
max read length	$1.000_{22177.0}$	$0.970_{21520.0}$	$0.931_{20636.0}$	$0.912_{20228.0}$	$1.000_{22169.0}$
flye total length	$0.967_{2150191.0}$	$0.830_{1845803.0}$	$0.777_{1727726.0}$	$1.000_{2222797.0}$	$0.943_{2095390.0}$
flye n50	$0.995_{11150.0}$	$0.995_{11145.0}$	$0.931_{10428.0}$	$1.000_{11204.0}$	$0.977_{10951.0}$
flye mean coverage	$1.000_{19.5}$	$0.639_{12.5}$	$0.302_{5.9}$	$0.308_{6.0}$	$0.614_{12.0}$
svim structural variants	$1.000_{1697.0}$	$0.000_{0.0}$	$0.000_{0.0}$	$0.010_{17.0}$	$0.000_{0.0}$
AM-score	9.62	7.44	7.52	7.79	8.74

Table S15. Metrics for ^{5}mC DNA data set.

Tool	Dorado	f5c R.	f5c E.	Uncalled4	Dynamont
median delta ($\Delta\mu$)	$0.263_{32.5}$	$0.749_{92.5}$	$0.769_{95.0}$	$0.798_{98.5}$	$1.000_{123.5}$
mad delta ($\Delta\sigma$)	$0.824_{7.0}$	$0.706_{6.0}$	$0.706_{6.0}$	$0.647_{5.5}$	$1.000_{8.5}$
homogeneity	$0.000_{13.5}$	$0.333_{9.0}$	$0.333_{9.0}$	$0.407_{8.0}$	$0.259_{10.0}$
segmented reads	$1.000_{10003.0}$	$0.009_{95.0}$	$0.008_{80.0}$	$0.797_{7975.0}$	$1.000_{9998.0}$
truncated reads	$1.000_{0.0}$	$0.990_{95.0}$	$0.992_{80.0}$	$0.205_{7945.0}$	$0.000_{9998.0}$
min read length	$0.970_{6.0}$	$0.000_{200.0}$	$0.785_{43.0}$	$0.810_{38.0}$	$0.995_{1.0}$
n50 read length	$0.002_{113.0}$	$1.000_{54787.0}$	$0.003_{187.0}$	$0.002_{100.0}$	$0.002_{103.0}$
max read length	$1.000_{162495.0}$	$0.410_{66569.0}$	$0.002_{268.0}$	$0.001_{130.0}$	$0.337_{54791.0}$
flye total length	$0.000_{0.0}$	$0.000_{0.0}$	$0.000_{0.0}$	$0.000_{0.0}$	$0.000_{0.0}$
flye n50	$0.000_{0.0}$	$0.000_{0.0}$	$0.000_{0.0}$	$0.000_{0.0}$	$0.000_{0.0}$
flye mean coverage	$0.000_{0.0}$	$0.000_{0.0}$	$0.000_{0.0}$	$0.000_{0.0}$	$0.000_{0.0}$
svim structural variants	$0.000_{0.0}$	$0.000_{0.0}$	$0.000_{0.0}$	$0.000_{0.0}$	$0.000_{0.0}$
AM-score	5.06	4.20	3.60	3.67	4.59

References

[1] Richard Durbin et al. *Biological Sequence Analysis: Probabilistic Models of Proteins and Nucleic Acids*. Cambridge University Press, Apr. 1998, pp. 54–66. ISBN: 9780511790492.
DOI: 10.1017/cbo9780511790492.
URL: http://dx.doi.org/10.1017/CBO9780511790492.

[2] Mark Gales and Steve Young. "The Application of Hidden Markov Models in Speech Recognition". In: *Foundations and Trends® in Signal Processing* 1.3 (2007), pp. 195–304. ISSN: 1932-8354.
DOI: 10.1561/2000000004.
URL: http://dx.doi.org/10.1561/2000000004.

[3] Bing-Hwang Juang, S. Levinson, and M. Sondhi. "Maximum likelihood estimation for multivariate mixture observations of markov chains (Corresp.)" In: *IEEE Transactions on Information Theory* 32.2 (1986), pp. 307–309.
DOI: 10.1109/TIT.1986.1057145.

[4] Ronald K. Pearson et al. "Generalized Hampel Filters". In: *EURASIP Journal on Advances in Signal Processing* 2016.1 (Aug. 2016). ISSN: 1687-6180.
DOI: 10.1186/s13634-016-0383-6.
URL: http://dx.doi.org/10.1186/s13634-016-0383-6.

[5] L.R. Rabiner. "A tutorial on hidden Markov models and selected applications in speech recognition". In: *Proceedings of the IEEE* 77.2 (1989), pp. 257–286. ISSN: 0018-9219.
DOI: 10.1109/5.18626.
URL: http://dx.doi.org/10.1109/5.18626.

[6] Hiruna Samarakoon et al. "Flexible and efficient handling of nanopore sequencing signal data with slow5tools". In: *Genome Biology* 24.1 (Apr. 2023). ISSN: 1474-760X.
DOI: 10.1186/s13059-023-02910-3.
URL: http://dx.doi.org/10.1186/s13059-023-02910-3.
