# Supplementary material for: Dynamont: A comprehensive cross-species comparison of ONT segmentation tools
Source: Gigascience. 2026 Jan 19;15:giag005. doi: 10.1093/gigascience/giag005 (PMC13014473; doi:10.1093/gigascience/giag005)
Supplement: giag005_GIGA-D-25-00327_Original_Submission [file giag005_giga-d-25-00327_original_submission.pdf]

GigaScience

Dynamont: A Comprehensive Cross-Species Comparison of ONT Segmentation Tools

--Manuscript Draft--

**Manuscript Number:**	GIGA-D-25-00327
**Full Title:**	Dynamont: A Comprehensive Cross-Species Comparison of ONT Segmentation Tools
**Article Type:**	Research
**Funding Information:**	DFG EXC 2051 (Project-ID 390713860) — Dr.rer.nat. Kai Papenfort<br>SFB1076 (3.A06) — Dr.rer.nat. Manja Marz<br>BMBF (01GR2305B.TP7) — Dr.rer.nat. Manja Marz<br>TMWWDG (FKZ5575/10-9) — Mr Jannes Spangenberg<br>European Research Council (CoG-101088027) — Not applicable
**Abstract:**	Abstract<br>Background: Oxford Nanopore Technologies (Oxford Nanopore Technologies (ONT)) sequencing enables direct, long-read<br>sequencing of DNA and RNA, preserving nucleotide modifications. During basecalling, deep neural networks translate raw<br>nanopore signals into nucleotide sequences, internally segmenting the signal to align it with the corresponding bases. This is a<br>challenging task due to uneven motor protein rotation, signal variability, low-quality reads, and the presence of nucleotide<br>modifications. However, the signal to nucleotide assignment is critical for novel downstream signal analysis. Existing tools, such<br>as Tombo Resquiggle, f5c Eventalign, f5c Resquiggle, and Uncalled4, operate after basecalling and rely on event-based<br>segmentation and mapping approaches, that often fail to align low-quality or modified reads and lack confidence estimates for<br>segmentation accuracy.<br>Results: Here, we present a large-scale comparative study in which 5 segmentation tools, including our novel tool Dynamont, are<br>applied to 12 ONT-sequenced data sets spanning different kingdoms of life. Overall, we segmented 120.000 reads and evaluated the<br>tools performance on a combination of 12 signal and downstream assembly metrics. Our study is accompanied by a comprehensive<br>and extensible Supplement that summarizes all data sets, execution instructions, and evaluation results. We score the<br>segmentation results using an aggregated metric score, created from all our analysed metrics.<br>Conclusions: No tool delivered the best results for all data sets. We recommend a careful choice and normalization of evaluation<br>metrics to select the best segmentation tool as a critical step in the process of ONT signal segmentation. Across nearly all RNA data<br>sets, Dynamont outperforms other segmentation tools in terms of aggregated metric scores. For DNA data sets, however, the<br>performance is more variable, with mixed results observed across tools.<br>Key words: ONT; direct RNA sequencing; ONT signal; segmentation; resquiggling
**Corresponding Author:**	Jannes Spangenberg<br>Friedrich Schiller University Jena: Friedrich-Schiller-Universitat Jena<br>Jena, Thuringia GERMANY
**Corresponding Author Secondary Information:**	
**Corresponding Author's Institution:**	Friedrich Schiller University Jena: Friedrich-Schiller-Universitat Jena
**Corresponding Author's Secondary**	

Powered by Editorial Manager® and ProduXion Manager® from Aries Systems Corporation

**Institution:**	
**First Author:**	Jannes Spangenberg
**First Author Secondary Information:**	
**Order of Authors:**	Jannes Spangenberg
	Christian Höner zu Siederdissen
	Winfried Goettsch
	Lennart Köhler
	Liz Maria Luke
	Kai Papenfort
	Manja Marz
**Order of Authors Secondary Information:**	
**Additional Information:**	
**Question**	**Response**
Are you submitting this manuscript to a special series or article collection?	No
**Experimental design and statistics**<br><br>Full details of the experimental design and statistical methods used should be given in the Methods section, as detailed in our Minimum Standards Reporting Checklist. Information essential to interpreting the data presented should be made available in the figure legends.<br><br>Have you included all the information requested in your manuscript?	Yes
**Resources**<br><br>A description of all resources used, including antibodies, cell lines, animals and software tools, with enough information to allow them to be uniquely identified, should be included in the Methods section. Authors are strongly encouraged to cite Research Resource Identifiers (RRIDs) for antibodies, model organisms and tools, where possible.<br><br>Have you included the information requested as detailed in our Minimum Standards Reporting Checklist?	Yes

**Availability of data and materials**<br><br>All datasets and code on which the conclusions of the paper rely must be either included in your submission or deposited in publicly available repositories (where available and ethically appropriate), referencing such data using a unique identifier in the references and in the “Availability of Data and Materials” section of your manuscript.<br><br>Have you have met the above requirement as detailed in our Minimum Standards Reporting Checklist?	Yes
GigaScience has policies and guidelines in place for the use of generative AI-writing tools such as ChatGPT. If you have used such writing tools to assist with writing the manuscript this must be declared and cited in the text. Authors should not list AI-writing tools and other AI-assisted technologies as an author or co-author and should acknowledge that they are fully responsible for text generated or refined by AI-writing tools.<p><br><br>A summary of use (particularly in the introduction or among methods) needs to be included at the end of the paper, and the outputs should also be included as a supplementary file hosted in GigaDB or other open repositories. Please read our guidelines for more information. <p><br><br>By submitting to GigaScience, you are aware of the journal's AI-writing tools policy, and if you have declared use of such tools below, you have acknowledged this where appropriate in your manuscript and have made a summary of use and outputs available. <p><br><b>AI-assisted writing tools have been used in the preparation of this manuscript?	Yes

Manuscript

Click here to access/download;Manuscript;dynamont_manuscript.pdf

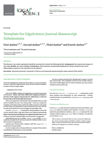

GigaScience, 2025, 1–21

doi: xx.xxxx/xxxx
Manuscript in Preparation
Paper

PAPER

Dynamont: A Comprehensive Cross-Species Comparison of ONT Segmentation Tools

Jannes Spangenberg[1], Christian Höner zu Siederdissen[1], Winfried Goettsch[1], Lennart Köhler[1], Liz Maria Luke[4], Kai Papenfort[4,5], and Manja Marz[1,2,3]

[1]*RNA Bioinformatics and High-Throughput Analysis, Friedrich Schiller University Jena, Leutragraben 1, 07743 Jena, Germany and* [2]*European Virus Bioinformatics Center 2, Leutragraben 1, 07743 Jena, Germany and* [3]*FLI Leibniz Institute for Age Research, Beutenbergstraße 11, 07745 Jena, Germany and* [4]*Friedrich Schiller University Jena, Institute of Microbiology, Jena, Germany and* [5]*Microverse Cluster, Friedrich Schiller University Jena, Jena, Germany*

Abstract

Background: Oxford Nanopore Technologies (Oxford Nanopore Technologies (ONT)) sequencing enables direct, long-read sequencing of DNA and RNA, preserving nucleotide modifications. During basecalling, deep neural networks translate raw nanopore signals into nucleotide sequences, internally segmenting the signal to align it with the corresponding bases. This is a challenging task due to uneven motor protein rotation, signal variability, low-quality reads, and the presence of nucleotide modifications. However, the signal to nucleotide assignment is critical for novel downstream signal analysis. Existing tools, such as `Tombo Resquiggle`, `f5c Eventalign`, `f5c Resquiggle`, and `Uncalled4`, operate after basecalling and rely on event-based segmentation and mapping approaches, that often fail to align low-quality or modified reads and lack confidence estimates for segmentation accuracy.

Results: Here, we present a large-scale comparative study in which 5 segmentation tools, including our novel tool `Dynamont`, are applied to 12 ONT-sequenced data sets spanning different kingdoms of life. Overall, we segmented 120.000 reads and evaluated the tools performance on a combination of 12 signal and downstream assembly metrics. Our study is accompanied by a comprehensive and extensible Supplement that summarizes all data sets, execution instructions, and evaluation results. We score the segmentation results using an aggregated metric score, created from all our analysed metrics.

Conclusions: No tool delivered the best results for all data sets. We recommend a careful choice and normalization of evaluation metrics to select the best segmentation tool as a critical step in the process of ONT signal segmentation. Across nearly all RNA data sets, `Dynamont` outperforms other segmentation tools in terms of aggregated metric scores. For DNA data sets, however, the performance is more variable, with mixed results observed across tools.
Key words: ONT; direct RNA sequencing; ONT signal; segmentation; resquiggling

Background

ONT sequencing has gained widespread adoption due to several advantages: it enables cost-effective and portable sequencing, produces ultra long reads, enables direct RNA sequencing, retains native nucleotide modifications, and allows for live basecalling during experiments. Oxford Nanopore sequencing captures changes in electrical current over time as nucleotide sequences pass through a biological nanopore embedded in a membrane, Fig. 1. A sensor continuously measures these current fluctuations, which are characteristic of the nucleotide context within the pore. Depending on the sequencing settings, the data is sampled at 4-5 kHz. In the case of DNA sequencing, 400 bases per second pass through the pore (for RNA 130 bases per second). The raw data, also called ONT signal, is stored in ONTs `pod5` file format. A typical analysis pipeline begins with basecalling the raw `pod5` data using basecallers like `Dorado`. This is followed by various downstream tools for quality filtering, correction, and the detection of nucleotide modifications [1, 2, 3].

Despite its strengths, ONT sequencing is challenged by relatively high error rates [4]. These errors can arise from multiple sources:

Compiled on: August 19, 2025.
Draft manuscript prepared by the author.

Key Points

- This is the first point
- This is the second point
- One last point.

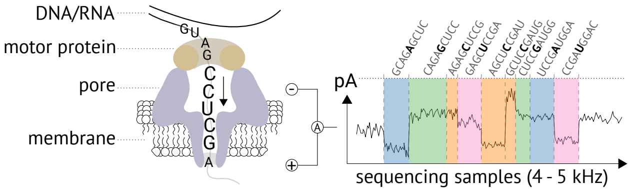

Figure 1. Nucleotide sequences pass the ONT nanopore directed by the motor protein upon an electrical field applied to the membrane. Nine nucleotides (or five for the old flow cell) are present in the pore at a time, while the ion flow is measured by a sensor in pico ampere (pA), leading to a characteristic sequencing signal. The time a 9-mer is present in the nanopre is unregular and the signal of different nucleotides can be similar.

a) modified nucleotides that deviate from canonical signal models. Currently, around 170 different RNA modifications are known in the modomics database [5]. ONT provides basecalling models for only four of those: N^6-methyladenosine (m6A), pseudouridine (psU), inosine (ino), 5-methylcytidine (m5C). b) Another error source is the timely uneven translocation of molecules through the nanopore, due to the motor protein dynamics. This causes nucleotide signals to differ in length, Fig. 1. In the worst case a nucleotide sequence can get stuck in the pore, stopping the sequencing of that read. c) Also, homopolymer regions are a fundamental problem in ONT sequencing [6]. In the new R10 and RP4 pores, 9 nucleotides are measured at once. A homopolymer stretch above 9 nucleotides is hard to predict, as it will produce a homogeneous sequencing signal. d) Finally, and of major importance for this work, are inaccuracies in the segmentation of the raw signal. `Dorado` can provide a low resolution signal segmentation when using the `-emit-moves` parameter.

Segmentation of the raw ONT signal is particularly critical when analysing the raw signal directly, e.g., the signals of nucleotide modifications, for which no prediction tool exists yet. Improper segmentation can propagate errors into downstream analyses. However, current segmentation tools are still underdeveloped, and their performance has not been systematically benchmarked across diverse data sets and conditions.

In this work, we benchmark five segmentation tools: `Tombo Resquiggle` (by ONT), `f5c Eventalign` [7], `f5c Resquiggle` [7], `Uncalled4` [8], and our proposed method `Dynamont`. While they all aim to align raw ONT signals to nucleotide sequences, they vary substantially in algorithm and requirements. Except for `Dynamont`, all tools rely on signal pre-processing via event detection, typically using a sliding window t-test originally implemented in `Scrappie`, an early ONT basecaller.

`Tombo Resquiggle` requires prior mapping of reads to a reference sequence. It applies `Scrappie`'s event detection as a preprocessing step, followed by a forward-only hidden Markov model (HMM) pass. Segmentation is performed using k-mer-based signal distribution models that associate raw nanopore signal segments with expected current levels. `f5c Eventalign` is an optimized reimplementation of `nanopolish eventalign`. It also requires read mapping to a reference sequence and uses `Scrappie`'s event detection for signal preprocessing. Like `Tombo Resquiggle`, it applies an HMM forward pass and segments the nanopore signal using k-mer-based signal models. `f5c Resquiggle` is closely related to `f5c Eventalign` but operates without requiring a mapping to a reference genome. Instead, it uses the basecalled reads directly for segmentation. It applies `Scrappie`'s event detection and an HMM forward pass to align the detected events to the read sequence. `Uncalled4` also requires read-to-reference mapping and implements `Scrappie`'s event detection. However, instead of using an HMM, it employs a dynamic time warping (DTW) algorithm to align the signal to the reference sequence, using an adaptive banded dynamic programming approach. `Dynamont` does not require any mapping or event detection. It directly uses the basecalled reads to align to the raw nanopore signal by performing both the forward and backward passes of an HMM. This full forward-backward algorithm enables the calculation of posterior probabilities for each segment, allowing for more refined segmentation and confidence estimates, Fig. 2.

Tool comparison

These methodological differences lead to significant divergence in a) where in the raw read signal the segmentation is assigned to, Fig. 2; b) number of reads segmented or truncated (due to filtered reads - unmapable or low quality) Tab. 2; c) what data is required as input, Fig 3; d) and especially, handling data from different organisms and sources, Fig. 7. Full technical details for each tool are provided in the Methods.

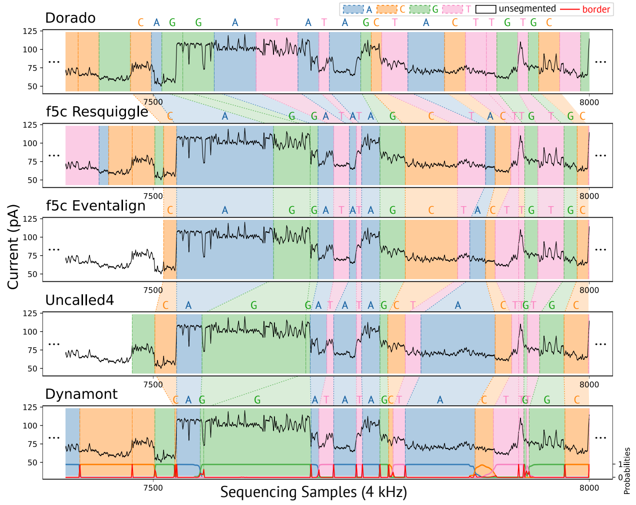

Figure 2. Immense differences of various tool segmentations at the example of an RNA004 *H. sapiens* read fragment. The central base of the aligned k=9-mer is colored. For segmentation improvement, the tool `Dorado` is input for all other tools. `f5c Eventalign` and `Uncalled4` do not segment the read completely and neglect read information. `Tombo Resquiggle` is not displayed in the figure, as it is not able to segment RNA004 reads. `Dynamont` is the only tool which returns additional segmentation probabilities, i.e. the border probability (red) or nucleotide probabilities.

Kovaka *et. al* [8] introduced a metric to measure the segmentation similarity between tools, the Jaccard similarity/distance. Unfortunately, this metric can only be used when a common reference sequence onto which the reads are mapped is available. For the

mentioned tools, which perform reference mapping-independent, a comparison was so far not possible.

Here, we compare in a large-scale comparative study `Dorado` and the five segmentation tools `Uncalled4`, `f5c Eventalign`, `f5c Resquiggle`, `Tombo Resquiggle`, and our own tool `Dynamont`. We applied them to 12 ONT-seqeunced DNA and RNA data sets spanning RNA002, RNA004, and DNA (R10.4.1 5 kHz) sequencing protocols from different types of flow cells. We evaluated the tools by a combination of 13 metrics. Furthermore, we will evaluate the usability of the tools, as well as the memory and runtime usage.

Materials: Data Description

We included 12 data sets, across 7 different sources, Tab. 1. The data sets cover mammals (*H. sapiens*, fungi *S. cerevisiae* and *P. anserina*), bacteria (*E. coli* and *S. aureus*), a virus (SARS-CoV-2), a plant viroid (citrus exocortis viroid (CEVd)), a synthetic metagenome data set (Zymo hmw, containing DNA of fungi and bacteria), and synthetic data (IVT, *in vitro* transcribed). We basecalled all data sets using the `Dorado`[1] basecaller (v0.9.1). For each data set, we extracted 10,000 randomly selected reads. Each tool was given the same 10,000 randomly selected reads per data set. After basecalling, the number of reads can differ slightly from 10,000, as `Dorado` can split one read signal into two basecalled reads, Fig. S2. These data subsets are available via Zenodo.

RNA002 Data Sets

The following four data sets were directly RNA sequenced using ONTs R9.4.1 MinION FLO-MIN106 flow cells with the RNA002 protocol. We basecalled them using the `rna002_70bps_hac@v3` model, see Supplements.

The H. sapiens data set. was downloaded from the ENA using the project ID PRJEB40872 with accession ID ERR4706156 (wild type replicate 1) [9]. This dataset contains human transcriptomic ribonucleic acid (RNA) (total RNA) reads of the HEK293T cell line. As a reference sequence, we used the complementary deoxyribonucleic acid (DNA) (cDNA) GRCh38 reference by the ensembl (release 113) for all required mapping processes.

The E. coli K12 data set. This strain was grown aerobically in LB medium at 37 °C to OD600 of 1.0. Cells were harvested by addition of 0.2 volumes of stop mix (95 % ethanol, 5 % (v/v) phenol) and snap frozen in liquid nitrogen. Total RNA was isolated using SV total RNA purification kit (Promega - AM1907), digested with TURBO DNase (Thermo Fisher Scientific), and rRNA removed by Ribo-Zero rRNA Removal Kit (Illumina - MRZGN126) according to the manufacturers instructions. The amount of RNA was measured by Qubit RNA-HS (High Sensitivity)-Assay-Kit (ThermoFisher Scientific - Q32852) according to the manufacturer's instructions. Finally a poly A-tail was added to the RNAs by incubation of 400 ng RNA with 300 nM ATP and 2.5U *E. coli* Poly(A) Polymerase (NEB - M0276) for 30 min at 37 °C. The Poly-A tailed RNA products were again purified using the RNAClean XP beads 1:1 volume RNA reaction mix to bead volume (Beckman Coulter - A66514) following the manufacturer's instructions. The cleaned RNA was measured by Qubit RNA-HS (High Sensitivity)-Assay-Kit. RNA sequencing was performed following the protocol provided by Oxford Nanopore Technologies (Oxford, UK), using R9.4.1 chemistry flowcells (FLO-MIN106) and direct-RNA chemistry sequencing kit (SQK-RNA002). For library preparation we used 50ng of poly-A tailed RNA, prepared as described above, using the provided polyT (RTA) adapter. For all the mapping processes we used the *E. coli* K12 cDNA reference from ensembl release 60 GCA_000005845 ASM584v2.

The SARS-CoV-2 data set. This virus is the alpha variant and is available from the SRA under the project PRJNA907180 using the run accession SRR22476725. The data set contains RNA reads from vero cells of nasopharyngeal swabs[10]. For reference, the cDNA reference by ensembl (ASM985889v3).

The synthetic in vitro transcription (IVT) data set. The *in vitro* transcribed data contains unmodified RNA, created by synthetic double stranded DNA template (1297 bp). DNA was ordered (gBlocks GeneFragments, Integrated DNA Technologies) containing the sequences of different 5-mers (sequence is found in Zenodo, see Sec. Data-Availability). 200 ng of double stranded DNA template was used in 20 µl IVT reactions for 1 h using the the HighScribe T7 RNA synthesis Kit (NEB–E2040S), following the manufacturer's instructions. After IVT the DNA templates were digested by addition of 20 µl RNase-free water including 2 U of DNase I (NEC–M0303S) for 10 min at 37 °C. DNase digestion was stopped by addition of 5 mM EDTA (final concentration) and heat inactivation for 10 min at 75 °C. The RNA products were purified using the RNAClean XP beads 1:1 volume IVT/DNase reaction mix to bead volume (Beckman Coulter–A66514) following the manufacturer's instructions. The cleaned RNA was measured by Qubit RNA-HS (High Sensitivity)-Assay-Kit (ThermoFisher Scientific - Q32852) according to the manufacturer's instructions. Finally a poly A-tail was added to the cleaned RNA by incubation of 3 µg RNA with 1 mM ATP and 5 U *E. coli* Poly(A) Polymerase (NEB–M0276) for 30 min at 37 °C. The Poly-A tailed RNA products were again purified using the RNAClean XP beads 1:1 volume RNA reaction mix to bead volume (Beckman Coulter–A66514) following the manufacturer's instructions. The cleaned RNA was measured by Qubit RNA-HS (High Sensitivity)-Assay-Kit. RNA sequencing was performed following the instruction provided by Oxford Nanopore Technologies (Oxford, UK), using R9.4 chemistry flowcells (FLO-MIN106) and direct-RNA chemistry sequencing kit (SQK-RNA002). For library preparation we used 1 µg of poly-A tailed IVT RNA templates, prepared as described above, using the provided polyT (RTA) adapter.

RNA004 Data Sets

All data sets were directly RNA sequenced with ONTs MinION FLO-MIN004RA or FLO-PRO004RA flow cell using the RNA004 protocol. `Dorado` with the `rna004_130bps_supv5.1.0` model was used to basecall the data, see Supplements.

The H. sapiens data set. This universal human reference RNA benchmark data set is taken from the Garvan Institute Long Read Sequencing Benchmark Data. This data set contains *in vivo* direct RNA sequencing (DRS) reads, sequenced on a PromethION (FLO-PRO004RA) and is available via the amazon web service. For this data set the same reference was used as for RNA002.

The S. cerevisiae data set. The fungi originates from the BY4741 parental strain[11]. It contains *in vivo* RNA reads, sequenced on a FLO-MIN004RA using the RNA004 protocol and is available via the SRA under the project PRJNA1150648 using the run accession SRR30335016. The *S. cerevisiae* R64-1-1 cDNA reference from the ensembl (release 113) database was used.

The Citrus Exocortis Viroid (CEVd) data set. This data contains reads from small circular non-coding RNAs, which act as infectious pathogens in higher plants[12]. It was sequenced on the FLO-MIN004RA flowcell using the RNA004 protocol and is available via Zenodo (PV259). Here we used the CEVd reference sequence

1 https://github.com/nanoporetech/dorado

Table 1. Data set overview of read length statistics for 10,000 randomly selected reads from each of the 12 data sets. The R9.4.1 flow cells operate on k = 5-mers, the RP4 and R10.4.1 ones on k = 9-mers. IVT – *in vitro* transcription (synthetic); CEVd – citrus exocortis viroid; Zymo high molecular weight (HMW) – synthetic metagenome data set. The read length and quality is taken from the the basecalled reads after basecalling with `Dorado`.

Pore/ Sequencing kit	Data Set	Readlength Min	Readlength Median	Readlength N50	Readlength Max	Readquality Min	Readquality Median	Readquality Max
RNA R9.4.1/ RNA002	*H. sapiens*	5	734	1 122	21 695	1.0	16.9	31.0
	E. coli	5	88	398	31 807	1.0	8.6	38.9
	SARS-CoV-2	5	1 327	1 957	11 296	1.0	16.2	29.7
	IVT	5	596	989	4 566	1.0	14.8	29.8
RNA RP4/ RNA004	*H. sapiens*	5	816	1 550	111 593	2.0	20.2	37.9
	S. cerevisiae	5	158	1 381	114 662	2.0	16.0	29.6
	CEVd	7	136	190	13 344	2.0	13.9	27.6
	IVT	6	185	256	27 405	2.1	14.4	27.4
DNA R10.4.1/ kit V14	*H. sapiens*	102	2 671	22 168	253 314	2.0	23.4	44.6
	Zymo hmw	32	3 058	13 092	104 946	2.3	22.9	41.9
	S. aureus	90	2 164	7 717	109 657	5.8	24.8	44.4
	P. anserina	124	4 163	5 560	22 177	6.6	21.2	38.5

found in the ENA under the accession AJ490825.1. As the CEVd is a circular RNA, it introduces mapping complications if a read spans the end and start of the reference sequence. To counter this, the reference sequence was once concatenated with itself.

The synthetic IVT data set. This was synthetized CEVd RNA, free of any modifications[12]. It was also sequenced on the FLO-MIN004RA flowcell using the RNA004 protocol and is available via Zenodo (PV260). The same reference was used as for the CEVd data set.

DNA R10.4.1 Data Sets

We used the `dna_r10.4.1_e8.2_400bps_sup@v5.0.0` model to basecall the following data sets.

The H. sapiens data set. The DNA R10.4.1 5 kHz data set is obtained from the universal human reference benchmark data set Garvan Institute Long Read Sequencing Benchmark Data with the ID: NA24385 (HG002). The data set contains human DNA sequenced with the SQK-LSK114 kit on a PromethION flowcell with a coverage of ~40 %. As a reference, the GRCh38 toplevel dna ensembl (release 112) was used.

The Zymo high molecular weight (hmw) data set. This data set is taken from the ENA database with the project ID PRJEB64570[2], containing DNA sequenced on a FLO-MIN114 flowcell using the SQK-LSK114 sequencing kit and a sampling rate of 5 kHz. This data set contains DNA from a mix of organisms, representing a synthetic metagenomic data set: Pseudomonas aeruginosa (14 %), Escherichia coli (14 %), Salmonella enterica (14 %), Enterococcus faecalis (14 %), Staphylococcus aureus (14 %), Listeria monocytogenes (14 %), Bacillus subtilis (14 %), and Saccharomyces cerevisiae (2 %). We took the reference sequences from Sereika et. al [13].

The S. aureus data set. This bacteria data set is taken from the BioProject PRJNA1091452 with the run ID SRR31990262, which was published in Dabernig-Heinz *et al.* [14]. It is a Gram-positive spherically shaped bacterium sequenced on a R10.4.1 flow cell with the v14 sequencing kit (SQK-LSK114). The reference sequence is taken from the assembly ASM2249454v1.

The P. anserina data set. This ascomycete fungus genomic data from the CaDa-strain is taken from the BioProject PRJNA1216259 under the run ID SRR32173960 from Ament-Velásquez *et. al* [15]. The fungus was sequenced using a MIN-FLO114 flowcell with the SQK-NBD114.24 sequencing kit. We used the assembly provided in the study as a reference sequence.

Methods: Segmentation Tools in Detail

`Dorado`

`Dorado` is ONT's state-of-the-art basecaller and was used to generate the input for all of the compared segmentation tools. Standard basecaller models are trained on canonical bases A, C, G, and T/U. There are special models that were additionally trained on specific base modifications, e.g. for the DNA modifications N4-methylcytosine (4mC), 5-methylcytosine (5mC), 5-hydroxymethylcytosine (5hmC), and N^6-methyladenine (6mA) of 17 known modifications [16]. For the 170 RNA modifications, ONT provides models for m6A, psU, m5C, and ino. During basecalling, `Dorado` provides an optional segmentation (`-emit-moves` parameter) with a low resolution. This resolution results from downsampling the input signal, within the neural network, Fig. S1. The model's focus is to basecall the data, not to assign signals to nucleotides, as is the case for segmentation tools, see Fig. 3.

2 https://github.com/Kirk3gaard/2023-basecalling-benchmarks

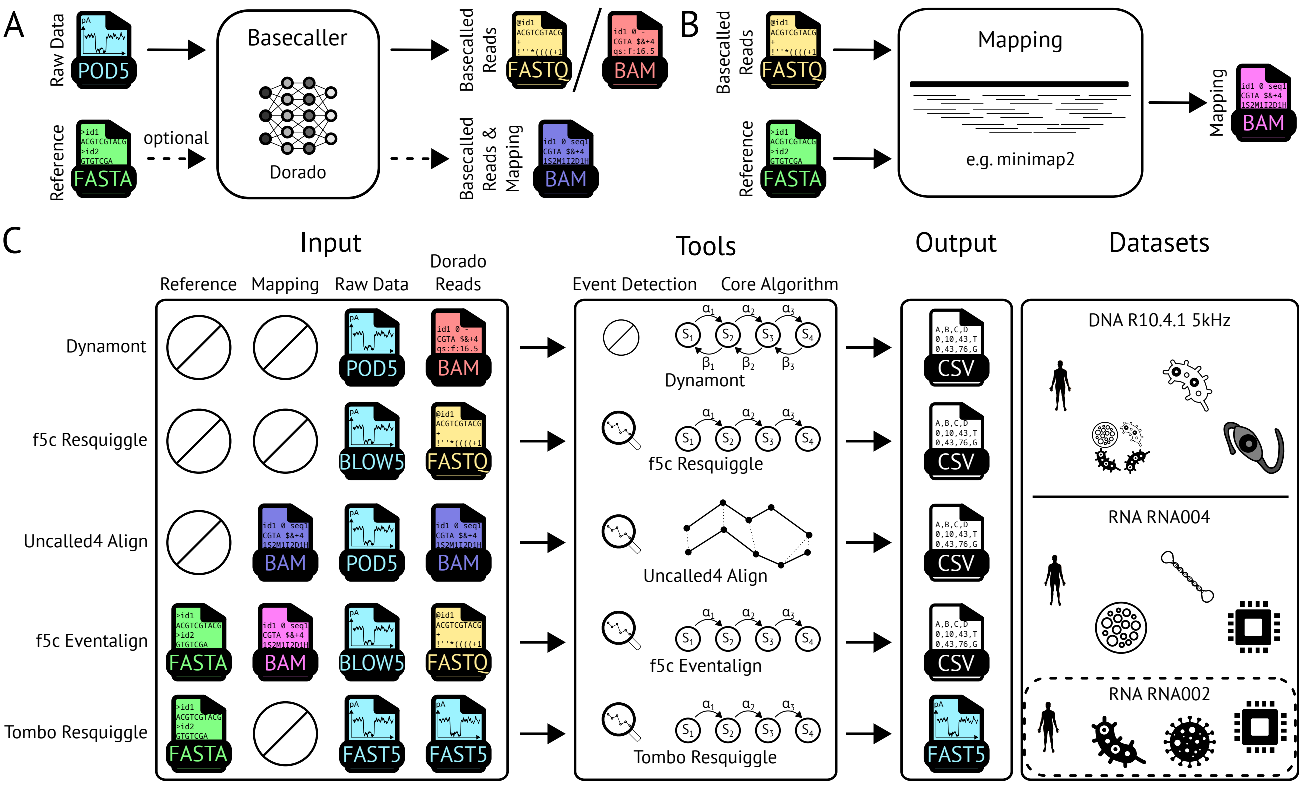

Figure 3. Overview of segmentation tools, their input, basic methods and applications. **A**: `Dorado` is input for improvement in all compared segmentation tools. `Dorado` translates ONT raw data (`*.pod5`, turquoise) into the basecalled reads. Depending on parameters and the input, reads can be stored in `*.fastq` (yellow), basecalled `*.bam` (red), or basecalled and mapped `*.bam` (blue), when dorado is provided with a reference sequence (green). Internally, `Dorado` utilizes `minimap2` to directly map the basecalled reads. **B**: After basecalling, reads (`*fastq`, yellow) can be optionally mapped to a reference sequence (*.fasta, green) using e.g. `minimap2` resulting in yet another bam file (`*.bam`, purple). **C**: Segmentation tools require different input files and formats. All of them need the raw data and the basecalled reads (again in different file formats). The output is provided in individual formats, often in tabular `*.csv` format. Except `Tombo Resquiggle`, every tool can process data sets of different chemistries and nanopores. `Tombo Resquiggle` is deprecated and cannot work with newer RP4 or R10 data. We executed the tools on 12 data sets from different origins and three different chemistries/pore types. All tools except `Dynamont` use ONTs event detection algorithm for pre-segmentation. `Dynamont` utilizes the full Forward-Backward algorithm, while other tools only implement the forward pass of HMM or DTW.

`Tombo Resquiggle` Only Works on R9 Data

`Tombo Resquiggle`[3] (v1.5.1) is a segmentation tool developed by ONT. `Tombo Resquiggle` is deprecated and does not support the signal file formats `multi fast5` and `pod5`, nor does it support sequencing protocols for the new RNA or DNA chemistries (RP4 or R10.4.1 pores). It utilizes a HMM forward pass to segment the signal and takes multiple inputs: the signal in the old `single fast5` format, the basecalled reads that needs to be added to the `single fast5` files, and a reference sequence in `fasta` format. `Tombo Resquiggle` preprocesses the data first in three steps: *(i) Mapping reads to a reference sequence:* The segmentation process begins by mapping reads to the reference sequence, to correct for sequencing errors. Unmapped nucleotides are not segmented by `Tombo Resquiggle`. *(ii) Normalizing the signal:* Read signals are normalized using the signal median shift and median absolute deviation (MAD) scaling to standardize signal levels across reads; and (iii) *Performing an event detection on the signal* The event detection is taken from `Scrappie`[4], an older ONT basecaller. It pre-segments the signal in homogeneous regions using a window-based t-test. The events reduce the runtime complexity from segmenting the whole signal to pre-segmented events. They are assigned with nucleotides using dynamic programming (DP) with an adaptive banding strategy, which further reduces complexity.

For the assignment, `Tombo Resquiggle` uses a pore model, which holds mean and standard deviation parameters for expected signal values per k-mer and pore used for sequencing. Using the pre-segmented events and expected signal values, `Tombo Resquiggle` calculates and sums up z-scores for mapped nucleotides. Afterwards the optimal segmentation is taken by following the path, that maximizes the sum of z-scores. `Tombo Resquiggle` does not provide any probability or confidence value for its segmentation.

`f5c Eventalign and f5c Resquiggle`

The `f5c` (v1.5) [7] package is an optimized reimplementation of `nanopolish`[5] [17] It also uses a forward pass of a HMM to segment the ONT signal. `F5c` has two modes, `f5c Eventalign` which is similar to `Tombo Resquiggle` and requires a mapping of the reads to a reference sequence. The other mode `f5c Resquiggle` segments the signal using the basecalled reads directly, without any mapping to a reference sequence. Otherwise, both modes work the same way using k-mer based pore models, and are similar to `Tombo Resquiggle`. They also pre-segment the ONT signal using `Scrappies` event detection algorithm, with individual parameters, to reduce runtime complextiy.

Additionally, `f5c` uses a different signal normalization. It is performed using a method-of-moments approach to adjust the pore model's mean and variance to fit the observed pA signal. Segmentation is performed on the events and signal using DP

3 https://nanoporetech.github.io/tombo/Resquiggle.html
4 https://github.com/nanoporetech/scrappie
5 https://github.com/jts/nanopolish

with an adaptive banding strategy. Within the HMM forward pass, emission probabilities are calculated using the Gaussian probability density function (PDF), while transition probabilities are estimated based on the ratio of detected events to reference k-mers.

`F5c` does not report any probability or confidence value for its segmentation. Alarmingly, low-quality and unmapped reads are neglected: they are excluded from further analysis and are not included in the output segmentation. Additionally, some reads are not fully segmented and can be truncated Fig. 2.

`Uncalled4` Utilizes Dynamic Time Warping

`Uncalled4` (v4.1) [8] segments the ONT signal using DTW and the basecallers optional low resolution segmentation. It has two modes, similar to `f5c Eventalign` and `f5c Resquiggle`, one that requires a mapping of reads to a reference sequence, and one without. We were not able to use the segmentation mode without mapping[6], which is why, in this work, we focus on the mode that requires mapping.

`Uncalled4` uses the mapping for error correction. Afterwards, it employs `Scrappies` event detection algorithm with custom parameters. `Uncalled4` performs a reference-guided signal normalization using a method-of-moments approach. This technique scales the events current level to match the expected mean and standard deviation of the corresponding reference k-mer, as determined by the `Dorado` segmentation. It then uses DTW through a DP approach that minimizes the absolute distance between the observed event current and expected current. `Uncalled4` uses an adaptive banding strategy derived from the basecaller segmentation, which reduces runtime complexity. The authors state, that by using the basecaller segmentation, they can be even more restricted with their band width than existing methods, making the algorithm more efficient. Finally, a linear regression step refines the segmentation, further improving the accuracy in modification detection and reference mapping. `Uncalled4` does not report any probability or confidence value for its segmentation.

`Dynamont`: Our HMM-based Segmentation Tool

Definitions used in Dynamont:

- **ONT signal:** $T = (t_0, \dots, t_{\mathcal{T}-1})$, time series of length $\mathcal{T}$.
- **Read:** $N = (n_0, \dots, n_{\mathcal{N}-1})$, nucleotide sequence of length $\mathcal{N}$ with $n_j \in \Sigma$.
- **DNA alphabet:** $\Sigma_{DNA} = \{A, C, G, T\}$.
- **RNA alphabet:** $\Sigma_{RNA} = \{A, C, G, U\}$.
- **k-mer set:** $C = \Sigma^k$, with cardinality $|C| = |\Sigma|^k$.
- **k-mer:** A sequence of length k, k-mer $\in C$.
- **Alignment:** t_i is aligned with $k_j = (n_j, \dots, n_{j+k-1})$, for $0 \leq i < \mathcal{T}$ and $0 \leq j < \mathcal{N} - k$.
- **Typical k values:** $k = 5$ for R9 pore, $k = 9$ for R10 or RP4.

Figure 4. Definitions relevant to the Dynamont segmentation algorithm.

`Dynamont` (v0.7.1) is a novel segmentation tool that leverages a HMM to probabilistically align basecalled nucleotide sequences N to nanopore sequencing signals T. Unlike existing approaches that rely on event detection and forward-only algorithms, `Dynamont` implements the full Baum-Welch algorithm [18, 19], performing both forward and backward passes to obtain the maximum a posteriori (Maximum A-Posteriori (MAP)) alignment between N and T. This enables more flexible and robust segmentation, especially beneficial for low-quality reads, short sequences, or regions affected by nucleotide modifications.

In the ONT context, the sequencing signal T represents a time series of current measurements generated as nucleotides translocate through a biological nanopore, Fig. 4. These observations are modeled as emissions from hidden states corresponding to overlapping k-mers in the nucleotide sequence. Transition probabilities govern state changes between k-mers, while emission probabilities are derived from k-mer-specific Gaussian distributions provided by ONT's pore model.

A distinguishing feature of `Dynamont` is that it does not use predefined event detection to segment the signal. While this increases computational complexity, it allows for more accurate determination of segment boundaries. Additionally, `Dynamont` calculates posterior probabilities for each aligned k-mer and signal segment, offering quantitative confidence scores, Fig. 2. These scores reflect the model's certainty in assigning a nucleotide to a segment and can be aggregated or visualized to provide further insights—capabilities not available in competing tools.

`Dynamont` is modular and includes several functionalities: a basic segmentation mode (used in this study), a resquiggle mode that jointly performs segmentation and error correction, Sec. S2.5, and a training module that enables users to retrain k-mer emission and transition parameters on custom data using the Baum-Welch algorithm, Sec. S2.6. This flexibility makes `Dynamont` highly adaptable to different data sets and sequencing chemistries, providing a robust foundation for downstream analyses such as RNA modification detection, error source identification, and signal interpretation.

Emission Probability using the Gaussian PDF

ONT signals are influenced by the nucleotide context within the pore and noise in measurement, leading to approximately Gaussian-shaped distributions. To efficiently model this variability, the Gaussian PDF ϕ, defined in Eq. 1, is used to describe the likelihood of observing a given signal t for a specific k-mer.

$$\phi(t, \mu_k, \sigma_k) = \frac{1}{\sqrt{2\pi\sigma_k^2}} \exp\left(-\frac{(t-\mu_k)^2}{2\sigma_k^2}\right) \tag{1}$$

For each k-mer in the nucleotide sequence N, the mean current μ_k and standard deviation σ_k are provided by ONT as part of publicly available k-mer models[7]. These parameters define the expected signal characteristics for the different sequencing chemistries. The Gaussian PDF assigns a probability to each pair of signal t and k-mer, allowing HMMs to evaluate how well a particular k-mer explains the observed signal. In `Dynamont`, ϕ is used to calculate emission probabilities, ensuring a probabilistic relationship between the observed signal T and the underlying sequence N.

`Dynamont` Forward-Backward Algorithm

`Dynamont`'s HMM consists of the states A (align) and E (extend), Fig. 5. State A moves (M) in both dimensions N and T, marking the start of a new segment, while E moves in T and halts (H) in N, extending a segment, Eq. 2- 3.

Between the states, the transitions $a1$, $e1$, and $e2$ exist. $a1$ starts a new segment. $e1$ is the first extension of a segment and $e2$ further extends the segment. This model architecture limits the minimum segment size to two data points of t. State emissions are calculated as shown before with Eq. 1 for both states.

6 raised as issue 04/2025, https://github.com/skovaka/uncalled4/issues/39

7 https://github.com/nanoporetech/kmer_models

The forward algorithm α of `Dynamont` iterates N and T from t_0 and k_0 to $t_{\mathcal{T}-1}$ and $k_{\mathcal{N}-k}$. For each iteration, α calculates a score using the paths that could be taken to end up in the current state, Eq. 2 [18, 19]. Within the dynamic programming implementation, a matrix exists for each state. The position i, j in each matrix stores the alignment score for the alignment of $(n_0, \ldots, n_{j+k-1})$ and $(t_0, \ldots, t_i)$. The emission to match k and t is denoted by $\begin{bmatrix} k_j \\ t_i \end{bmatrix}$. The initial position is $M_{0,0}$, denoted by $\begin{bmatrix} \epsilon_{j=0} \\ \epsilon_{i=0} \end{bmatrix}$. The final alignment score Z can be found in $E_{\mathcal{N}-k,\mathcal{T}-1}$.

$$
\begin{aligned}
A: \begin{pmatrix} M_j \\ M_i \end{pmatrix} &\rightarrow a1 \begin{pmatrix} M_{j-1} \\ H_{i-1} \end{pmatrix} \begin{bmatrix} k_j \\ t_i \end{bmatrix} \Bigg| \begin{bmatrix} \epsilon_{j=0} \\ \epsilon_{i=0} \end{bmatrix} \\
E: \begin{pmatrix} H_j \\ M_i \end{pmatrix} &\rightarrow e1 \begin{pmatrix} M_j \\ M_{i-1} \end{pmatrix} \begin{bmatrix} k_j \\ t_i \end{bmatrix} \Bigg| \; e2 \begin{pmatrix} H_j \\ M_{i-1} \end{pmatrix} \begin{bmatrix} k_j \\ t_i \end{bmatrix}
\end{aligned} \tag{2}
$$

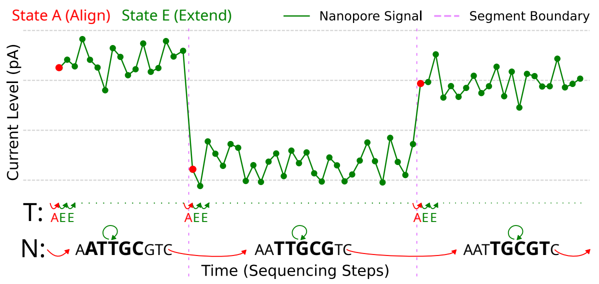

Figure 5. The forward pass functionality of `Dynamont`: The iteration starts at t_0 of signal T and k_0 of nucleotide sequence N. It ends at $t_{\mathcal{T}}$ and $k_{\mathcal{N}-k}$.

The backward algorithm β of `Dynamont` is derived from the forward algorithm. It is the second pass over the data, iterating from $t_{\mathcal{T}-1}$ and $k_{\mathcal{N}-k}$ toward t_0 and k_0 [18, 19]. The backward algorithm calculates a score using the paths that could be taken next starting from the current state. The initial position is denoted by $\begin{bmatrix} \delta^*_{j=\mathcal{N}-k} \\ \delta^*_{i=\mathcal{T}-1} \end{bmatrix}$ in position $E_{\mathcal{N}-k,\mathcal{T}-1}$. The final alignment score Z^* can be found in $M_{0,0}$.

$$
\begin{aligned}
A^*: \begin{pmatrix} M_j \\ M_i \end{pmatrix} &\rightarrow e1 \begin{pmatrix} H_j \\ M_{i+1} \end{pmatrix} \begin{bmatrix} k_j \\ t_{i+1} \end{bmatrix} \\
E^*: \begin{pmatrix} H_j \\ M_i \end{pmatrix} &\rightarrow a1 \begin{pmatrix} M_{i+1} \\ M_{i+1} \end{pmatrix} \begin{bmatrix} k_{j+1} \\ t_{i+1} \end{bmatrix} \Bigg| \; e2 \begin{pmatrix} H_j \\ M_{i+1} \end{pmatrix} \begin{bmatrix} k_j \\ t_{i+1} \end{bmatrix} \Bigg| \begin{bmatrix} \delta^*_{j=\mathcal{N}-k} \\ \delta^*_{i=\mathcal{T}-1} \end{bmatrix}
\end{aligned} \tag{3}
$$

To reduce the number of calculations, the forward and backward algorithm of `Dynamont` can be calculated only for a band, Fig. S4. Using this band, N is not fully iterated, which reduces the runtime complexity and memory usage.

Alignment Extraction using MAP path

Posterior probabilities P_Q are computed for each state Q of the forward algorithm and its corresponding state Q^* of the backward algorithm. To normalize these probabilities, they are standardized by the final alignment score Z, as defined in Eq. 4:

$$
P_{Q_{i,j}} = \frac{Q_{i,j} Q^*_{i,j}}{Z} \tag{4}
$$

The MAP path is then extracted using $P_{Q_{i,j}}$ with the Viterbi algorithm [19]. This path represents the most probable alignment of the sequence between N and T, providing the optimal alignment solution, Fig. S3.

Metrics

To evaluate the segmentation tools, we introduce metrics that will measure: a) how well a segment is placed; b) how many reads are (fully) processed; and c) how well subsequent tools can work with the output of the segmentation tools. We calculate the scores for only those reads, that were segmented by all tools for each data set respectively.

To answer how well segments are placed, we calculated the median delta, MAD delta, and segment homogeneity, Fig. 6. Our assumption is that nucleotides within the nanopore produce characteristic signals with respect to intensity and variation. A well placed segment should, therefore, separate regions within the signal, that differ in the signal level and variation. In other words, segments separate heterogeneous regions of the signal, while containing homogeneous regions. As a segment is flanked by two borders, for each border we looked six signal data points left and right of it, collected the median and MAD of these windows, and calculated the absolute difference between the windows, respectively. Both values are expected to be maximized by the tools. As the signal within a segment should be produced by one k-mer, it should be homogeneous in intensity and variation. To measure the homogeneity, we look at segments that span at least ten signal data points and calculate the standard deviation of the inner 80 %, leaving out data points close to the segment borders. A high homogeneity is equal to a low signal variation and vice-versa. The segment homogeneity metric is expected to be minimal. For each of these three metrics, many values are calculated per tool and data set. The median value is reported as a score for each tool and data set, Tab. 2.

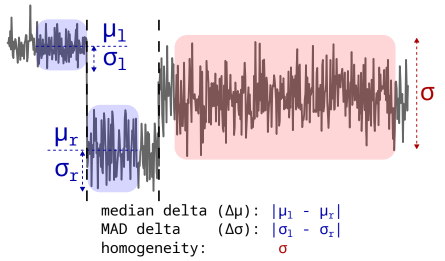

Figure 6. Placements of segments: The median and MAD differences of the windows left (width: 6 samples) and right (width: 6 samples) to a placed segment are calculated (blue). A higher difference is better, as nucleotide-specific signal segments should—at least in most cases—differ in signal level and variation from one another. A window size of 6 was chosen because it matches the smallest segment size used by `Dorado`, `f5c Eventalign`, and `f5c Resquiggle`. Additionally the homogeneity is calculated in a window within a segment (red). Since a segment should be internally homogeneous, a lower value is preferable. The homogeneity window covers the inner 80 % of each segment, chosen to exclude signals transitioning from one segment to the next when calculating the standard deviation.

To measure, how many reads are (fully) processed by each tool, we compared the numbers and lengths of input reads against the output. We decided to measure the number of segmented reads and truncated reads, as well as the minimal, N50, and maximal length of the resulting reads. For the metrics minimal read length and truncated reads, lower values are better, as they indicate fewer filtered short reads and incomplete reads respectively. This favors tools, that retain a large number of reads without excessive filtering and truncation. For all other metrics, the greater the value the better.

Finally, we wanted to evaluate, how well downstream tools could work with the results of the tools. We converted the output of the tools to `fasta` files and executed `flye` [20], an assembly tool, and `svim` [21], a tool to detect structural variants. For `flye`, we looked at

the total length, N50, and mean coverage of the resulting contigs and used them as metrics. For `svim`, we looked at the number of structural variants `svim` could find and used it as a metric. These metrics evaluate the possibility of additional data processing after segmentation.

Converting the metrics to scores. To enable fair comparisons across different metrics and data sets, each metric is converted into a normalized score ranging from 0 to 1. For every tool and data set, we determine the metric's maximum values and scale the interval from 0 to the maximum value linearly such that 0 corresponds to a score of 0 and the maximum value to a score of 1. For metrics where lower values indicate better performance—such as minimal read length or number of truncated reads—the scores are inverted so that lower values yield higher scores (i.e., a raw value of 0 maps to a score of 1.0 and the maximum raw value maps to 0.0). This transformation is applied consistently across all tools and data sets, allowing for a unified and intuitive performance comparison, see Tab. 2.

The aggregated metric score (AM-score). To obtain an overall performance measure, we compute an aggregated metric score for each tool and data set. This score is calculated as the sum of all individual normalized metric scores for the respective tool on the given data set. By aggregating the metric scores in this way, we capture a tool's overall performance across multiple evaluation criteria in a single value, facilitating direct comparisons between tools. A higher aggregated score indicates better performance across the evaluated metrics for the respective tool and data set, see Fig. 7.

Computational resources

The calculations were executed on a single machine with a 12-core AMD Ryzen 9 3900X CPU, the 6.1.0-30-amd64 x86_64 Kernel; 4 dual in-line memory modules of Kingston 64 GB 2666 MHz; and a PRIME B550-PLUS Mainboard. Each tools was executed using 20 threads.

Usability

We installed all tools without root privileges, using either `Conda` or pre-compiled binaries, on a Debian GNU/Linux 12.11 (64-bit) system. The ease of installation and execution varies significantly depending on the user's system setup, experience, and familiarity with the programming languages the tool relies on. Despite these factors, publicly available software should strive to be as user-friendly as possible. To support this, we documented our experience installing and running each tool, summarized in Tab. 3.

Analyses

We analyzed 12 data sets using 5 segmentation tools and the basecaller `Dorado`, evaluating performance across 12 metrics. The data sets cover a broad range of sequencing contexts, including: 4 DNA samples sequenced on the R10.4.1 pore using the DNA 5 kHz protocol; 4 RNA samples sequenced on the RP4 pore using the RNA004 protocol; and 4 RNA samples sequenced on the R9.4.1 pore using the RNA002 protocol. Our comparison provides detailed insights into how each segmentation tool performs across these diverse data sets and metrics. The data sets were selected to represent not only different biological origins – from various kingdoms of life – but also different sequencing strategies used in ONT workflows. Our metrics assess three key aspects of tool performance: the quality of signal segmentation, the efficiency of read processing, and the compatibility with downstream analysis workflows. For each combination of data set and metric, we normalized the raw results from all tools to a 0.0–1.0 range. This approach, similar to a z-score transformation followed by min-max normalization, ensures fair comparisons across data sets, tools, and metrics (Tab. 2). Additional detailed tables for all data sets are provided in the Supplementary Material, Tab. S1-S11. Finally, for each tool and data set, we computed an AM-score by summing the individual normalized scores, corresponding to the last row in Tab. 2. This allows a concise, high-level comparison of overall performance across all metrics (Fig. 7).

Segmentation tool performance is diverse regarding different data sets and quality metrics

All evaluated tools are summarized in Fig. 3 and Tab. 3. While tuning each tool for optimal performance on every data set would be ideal, it falls beyond the scope of this evaluation. To ensure a fair comparison, we used each tool's default settings and adjusted only those parameters that minimize read filtering, where possible. The exact execution commands are provided in Sec. S2.1. In practical applications, we recommend testing multiple parameter configurations, tailored to specific data sets and goals. In case of tools that require mapping, we also recommend trying different reference sequences and mapping parameters for optimal processing. For cases where a tool could not run on a particular data set or in a specific mode, we made an effort to debug the source code and, contacted the authors for support. Based on these experiences, we have evaluated the ease of installation and execution for each tool in Tab. 3.

`Dorado`

`Dorado` can generate a low-resolution segmentation using its "move table" when the `-emit-moves` parameter is enabled. However, the resulting segmentation is less precise compared to dedicated segmentation tools, as shown in Fig. 2.

For the *H. sapiens* RNA004 data set, `Dorado` performs worst in segmentation accuracy, with a median delta score of just 0.24—approximately four times lower than the other tools—and a homogeneity score of 0.0, as reported in Tab. 2. While its raw homogeneity value is similar to that of other tools, its contrast score significantly lags behind. `Dorado`'s MAD delta score reaches 0.75, comparable to all tools except `Dynamont`. Because `Dorado`'s basecalled reads serve as input for all other segmentation tools, it naturally achieves the highest scores for metrics related to read length and count. This dependency also explains `Dorado`'s strong performance in metrics evaluating downstream tool compatibility.

`Dorado`'s strong performance in both read-level and downstream tool metrics contributes to its consistently high AM-scores across all data sets, Fig. 7. It achieves the highest average AM-score overall, with a value of 9.05. However, since `Dorado`'s basecalled reads serve as the input for all segmentation tools, its performance effectively represents an upper bound for a lot of metrics in subsequent analyses. For this reason, we display `Dorado` separately in Fig. 7 and exclude it from detailed comparisons with the segmentation tools. Despite its strengths, Tab. 2 shows that `Dorado` underperforms in key segmentation metrics, highlighting its limited ability to achieve high accuracy in signal segmentation.

`Tombo Resquiggle`

`Tombo Resquiggle` also uses a forward-pass-only HMM for segmentation, preceded by an event detection step that pre-processes the raw signal. However, since `Tombo Resquiggle` is officially deprecated and no longer maintained, it is excluded from the analysis of the RNA004 *H. sapiens* data set presented here. Despite

Table 2. Examplarily comparison of segmentation tools on the data set *H. sapiens* RNA004. The metric score each ranges from 0.0 (worst) to 1.0 (best). Absolute values are written subscripted. `Tombo Resquiggle` could not process RNA004 reads. All segmentation tools use the output of `Dorado` as input, which often prevents them from surpassing `Dorado` in read statistics or downstream metrics. As seen in all data sets, dedicated segmentation tools greatly improve the segmentation metrics (median delta, MAD delta, and homogeneity). However, a major drawback for these tools are the metrics regarding read statistics. `Dynamont` has the smallest drawback, and shows an exception for minimum read length. Notice, that the other tools neglect short reads, which can be disadvantageous, e.g. when working with small RNAs. The results of the other data sets can be found in Tab. S1-S11.

Tool	Dorado	f5c R.	f5c E.	Uncalled4	Dynamont
median delta	0.240 $_{15.5}$	0.915 $_{59.0}$	0.938 $_{60.5}$	0.946 $_{61.0}$	1.000 $_{64.5}$
MAD delta	0.750 $_{6.0}$	0.688 $_{5.5}$	0.688 $_{5.5}$	0.750 $_{6.0}$	1.000 $_{8.0}$
homogeneity	0.000 $_{16.0}$	0.219 $_{12.5}$	0.219 $_{12.5}$	0.188 $_{13.0}$	0.094 $_{14.5}$
segmented reads	1.000 $_{10\,088.0}$	0.916 $_{9\,245.0}$	0.881 $_{8\,884.0}$	0.925 $_{9\,329.0}$	1.000 $_{10\,088.0}$
truncated reads	1.000 $_{0.0}$	0.000 $_{9\,245.0}$	0.041 $_{8\,865.0}$	0.026 $_{9\,005.0}$	0.662 $_{3\,124.0}$
min length	0.975 $_{5.0}$	0.000 $_{201.0}$	0.627 $_{75.0}$	0.667 $_{67.0}$	0.970 $_{6.0}$
N50 length	1.000 $_{1\,550.0}$	0.957 $_{1\,483.0}$	0.897 $_{1\,391.0}$	0.932 $_{1\,445.0}$	0.997 $_{1\,546.0}$
max length	1.000 $_{111\,593.0}$	0.374 $_{41\,777.0}$	0.086 $_{9\,609.0}$	0.090 $_{10\,027.0}$	1.000 $_{111\,594.0}$
flye total length	1.000 $_{100\,285.0}$	0.939 $_{94\,190.0}$	0.789 $_{79\,174.0}$	0.983 $_{98\,534.0}$	0.931 $_{93\,379.0}$
flye N50	0.974 $_{2\,786.0}$	0.955 $_{2\,732.0}$	0.986 $_{2\,819.0}$	1.000 $_{2\,860.0}$	0.978 $_{2\,797.0}$
flye mean coverage	1.000 $_{7.6}$	0.980 $_{7.5}$	0.565 $_{4.3}$	0.549 $_{4.2}$	0.993 $_{7.6}$
`svim` structural variants	0.584 $_{143.0}$	0.849 $_{208.0}$	1.000 $_{245.0}$	0.159 $_{39.0}$	0.449 $_{110.0}$
aggregated metric score (AM-score)	9.52	7.79	7.72	7.21	10.07

this, we chose to include `Tombo Resquiggle` in our broader study for completeness and historical comparison. In the RNA002 data sets, Tab. S1-S4, `Tombo Resquiggle` consistently underperforms relative to the other segmentation tools. It records the lowest AM-score in three out of four data sets and ranks second to last in the *E. coli* data set. These results reflect both its aging architecture and its limitations in handling more recent data and chemistries. `Tombo Resquiggle` is installable via `Conda`, but requires multiple data conversions, as it requires single `fast5` files as input. The output of `Tombo Resquiggle` is also stored in the `fast5` format, which is not human readable, as e.g. tabular `csv` files.

`f5c Resquiggle`

`f5c Resquiggle` is a segmentation tool that leverages event detection and a forward-only HMM to align and segment the ONT signal directly using basecalled reads (in `fastq` format). `f5c Resquiggle` significantly improves segmentation performance on the RNA004 *H. sapiens* data set compared to `Dorado`, achieving a median delta score of 0.915 and a MAD delta score of 0.688, as shown in Tab. 2. While slightly behind the top-performing segmentation tools in these metrics, it performs well in homogeneity, tying with `f5c Eventalign` at a leading score of 0.219. `f5c Resquiggle` filters approximately 10 % of the reads and exhibits the highest rate of truncation among all tools. Many short reads are excluded—while the data set includes reads as short as 5 nt, the shortest read segmented by `f5c Resquiggle` is 201 nt. Similarly, the longest read in the data set (111,593 nt) is only segmented up to 41,777 nt by `f5c Resquiggle`. As a result, the N50 of segmented reads is reduced by about 5 %. Despite the filtering and truncation, `f5c Resquiggle` performs very well in downstream tool compatibility, achieving scores ranging from 0.85 to 0.98, indicating robust integration in typical ONT analysis workflows.

`f5c Resquiggle` consistently ranks among the top segmentation tools across all data sets. In terms of the AM-score, it secures second place among segmentation tools with an average score of 6.96. Its best performance is observed in the RNA002 IVT data set. Additionally, it ranks second in multiple data sets, including RNA002 *E. coli* and SARS-CoV-2, RNA004 *H. sapiens*, *S. cerevisiae*, and IVT, as well as the DNA Zymo HMW and *S. aureus* data sets, Fig. 7.

`f5c Eventalign`

`f5c Eventalign` closely resembles `f5c Resquiggle` in its segmentation approach, relying on event detection and a forward-only HMM. However, unlike `f5c Resquiggle`, which segments the signal directly using basecalled reads, `f5c Eventalign` requires reads to be mapped to a reference genome prior to segmentation. This additional mapping step slightly improves segmentation in some metrics, such as achieving a higher median delta score of 0.938 on the RNA004 *H. sapiens* data set. The MAD delta and homogeneity scores remain unchanged at 0.688 and 0.219, respectively—identical to those of `f5c Resquiggle`. Notably, like other tools, `f5c Eventalign` outperforms `Dorado` in signal segmentation, achieving a nearly four times higher median delta score. In terms of read-level performance, `f5c Eventalign` filters out more reads than `f5c Resquiggle` due to its dependence on mapping. While it truncates a significant number of reads, it retains more short reads than `f5c Resquiggle`, with the shortest segmented read being 75 nt long. However, a major drawback is its handling of long reads. The longest segmented read in this data set is only 9,609 nt – just 8.6 % of the true longest read (111,593 nt). This limitation stems from the initial mapping step, as demonstrated by `f5c Resquiggle`'s ability to segment reads over 41,000 nt in length. `f5c Eventalign` shows mixed performance in downstream tool compatibility. For example, `flye` assemblies generated from its outputs are shorter in total length and coverage, though the N50 is improved. Structural variant detection using `svim` also identifies more variants, likely due to base modifications introduced during the mapping step, which alters the nucleotide sequences.

On average, `f5c Eventalign` ranks third among segmentation tools, with an AM-score of 6.36 across all data sets (Fig. 7). It achieves the best overall score on the DNA *H. sapiens* data set, outperforming the second-best tool by 12 %, and secures second place on the RNA002 *H. sapiens* data set.

`Uncalled4`

`Uncalled4` utilizes event detection for preprocessing but differs from other tools by applying a forward-pass DTW algorithm instead of an HMM for segmentation. Like `f5c Eventalign`, it requires a prior mapping step before segmentation can begin. In the RNA004 *H. sapiens* data set, `Uncalled4` achieves higher segmentation accuracy than both `f5c Eventalign` and `f5c Resquiggle`. It

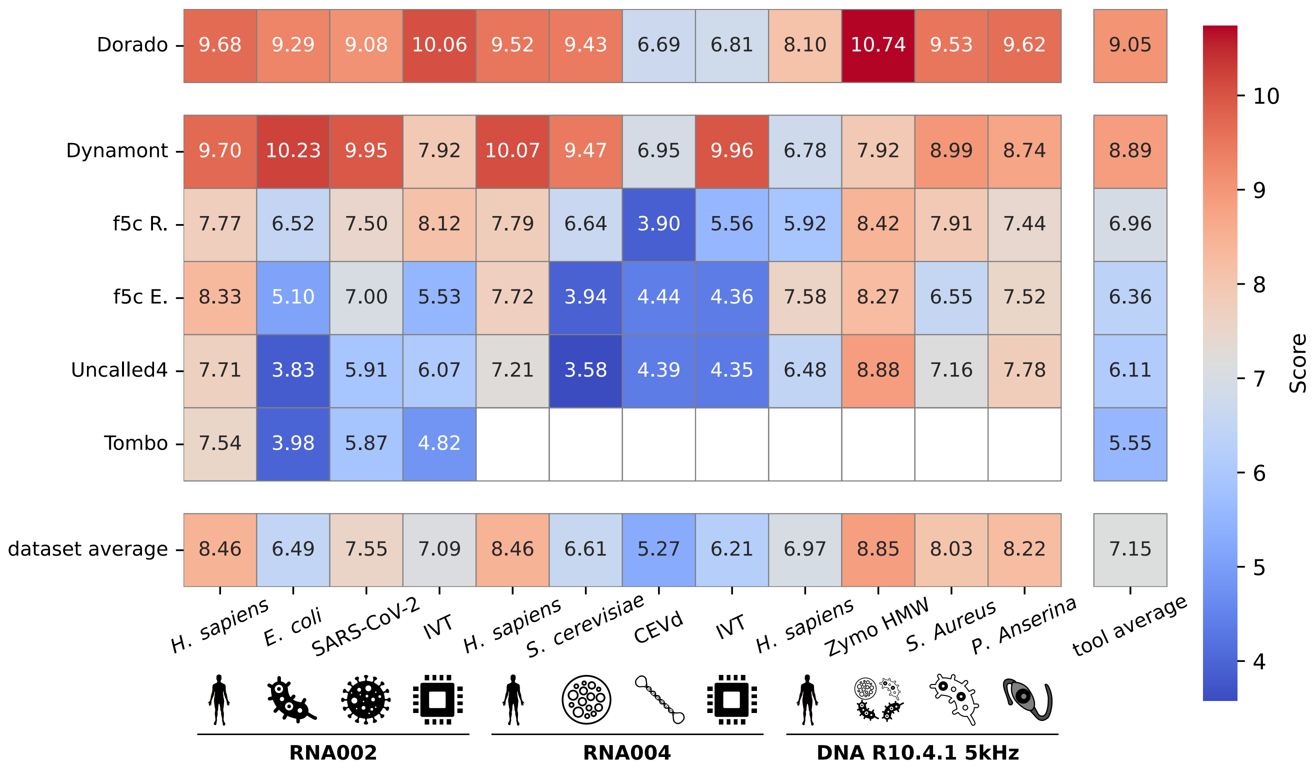

Figure 7. The aggregated metric score (AM-score) for each tool and data set. Tools are sorted for their mean value across all data sets. The AM-score is the sum of all metric scores, as shown in Tab. 2. It describes the overall segmentation performance per tool and data set. The best average AM-score (last column) across all segmentation tools (except `Dorado`, which we do not declare a segmentation tool) is achieved by `Dynamont`.

reaches a median delta score of 0.946 and a MAD delta of 0.75. The homogeneity score is slightly lower at 0.188, though still within a competitive range. From a read-level perspective, `Uncalled4` segments 9,329 reads—more than `f5c Resquiggle` —indicating strong performance despite the mapping prerequisite. The shortest successfully segmented read is 67 nt long. Truncation rates and N50 values are comparable to those of `f5c Eventalign` and `f5c Resquiggle`. However, the longest segmented read (10,027 nt) is 91 % shorter than the data set's longest read, likely due to limitations imposed by the mapping step shared with `f5c Eventalign`. In downstream analysis, `Uncalled4` delivers competitive results. Its assembly from `flye` has the second-longest total length after `Dorado`, and it achieves the highest N50 of 2,860. The mean coverage, however, remains on the lower end, similar to `f5c Eventalign`. Notably, `Uncalled4` receives the lowest score (0.159) in the `svim` structural variant detection metric, suggesting limitations in preserving variant-relevant features.

Across all data sets, `Uncalled4` attains an average AM-score of 6.11, placing it fourth among the segmentation tools evaluated (Fig. 7). It performs particularly well in the DNA Zymo HMW data set, where it achieves the highest overall score, and ranks second in the *P. anserina* data set.

`Dynamont`

`Dynamont` does not rely on event detection or pre-mapping. Instead, it directly segments the ONT signal using a forward and backward pass of a custom HMM, aligning the signal to the basecalled reads. `Dynamont` achieves the highest segmentation quality among all tools, with a median delta approximately 6 % higher and a MAD delta around 33 % higher than the next best method. Its homogeneity score is slightly lower than the top-performing tools but still outperforms `Dorado` and remains competitive overall. `Dynamont` exhibits excellent performance in preserving read integrity. It segments all 10,088 reads without discarding or truncating a significant portion, resulting in roughly 66 % fewer truncations than other tools. It is able to process both very short reads (as short as 6 nt) and very long reads (up to ~111,500 nt). As a result, its N50 read length (1,550 nt) closely matches the input read length distribution provided by `Dorado`. Although slight differences in read structure lead to a marginally shorter total assembly length with `flye` (93,379 nt), `Dynamont` produces a slightly higher assembly N50 (2,797 nt) and near-identical coverage (score: 0.993). In structural variant detection with `svim`, it shows slightly reduced performance (score: 0.449). Still, `Dynamont` reaches the highest AM-score on the RNA004 *H. sapiens* data set with a score of 10.07, surpassing even `Dorado`.

Across all data sets, `Dynamont` achieves the highest average AM-score among the segmentation tools, with a score of 8.89, approximately 28 % higher than `f5c Resquiggle`. It performs best in the RNA002 *H. sapiens*, *E. coli*, and SARS-CoV-2 data sets; RNA004 *H. sapiens*, *S. cerevisiae*, CEVD, and IVT; as well as the DNA *S. aureus* and *P. anserina* data sets. In 7 out of these 9 RNA data sets, `Dynamont` outperforms `Dorado` itself.

Tool Usability and Runtime Performance

We evaluated each tool not only by its analytical performance but also in terms of usability, runtime, and memory efficiency. These practical aspects are especially important when deploying signal segmentation pipelines on diverse systems or large data sets. Our experiences are summarized in Tab 3. These results do apply for the presented data sets with a read count of 10,000 reads per data set and their specific read statistics, Tab. 1.

Installation and Setup.

All tools (`Tombo Resquiggle`, `f5c Resquiggle`, `f5c Eventalign`, `Uncalled4`, and `Dynamont`) were easy to install via `Conda` or as

Table 3. Overview of segmentation tool performance. For runtime and memory we report the minimum, maximum, and median statistics of the tools across the 12 data sets. `Dorado` utilizes the GPU for the calculations, which is why we reported the virtual RAM (VRAM) in brackets additionally to the RAM usage. To ensure comparability, both `f5c Eventalign` and `f5c Resquiggle` were executed on the CPU, though GPU execution is possible and results in faster runtimes. Runtime and memory measurements were performed on a system with: CPU: 12-core AMD Ryzen 9 3900X; Kernel: 6.1.0-30-amd64 x86_64; RAM: Kingston 64 GB 2666 MHz; Mainboard: PRIME B550-PLUS, GPU: NVIDIA GeForce RTX 2080 Ti. We assessed the usability of each tool by expressing our experience through emojis. All tools were easily installable. For `f5c Resquiggle` and `f5c Eventalign` the input data format needed to be converted, from the state-of-the-art `pod5` to the older `slow5` format. `Tombo Resquiggle` requires the input data to be converted to an even older `single fast5` format. Additionally, the output format is stored in the same (user-unfriendly) format.

Segmentation	Version	Usability	Runtime			RAM usage in GB (VRAM usage)			Source	Year
Tool			Min	Max	Median	Min	Max	Median		
`Dorado`	0.9.6	😄	31s	11m 11s	2m 36s	1.8 (6.8)	3.2 (10.7)	2.5 (10.5)	-	2025
`Tombo Resquiggle`	1.5.1	🙁	6m 4s	10m 45s	8m 55s	2.1 (0.0)	68.3 (0.0)	2.4 (0.0)	-	2017
`f5c Resquiggle`	1.5.0	🙂	4s	59s	12s	0.1 (0.0)	2.2 (0.0)	0.5 (0.0)	[7]	2020
`f5c Eventalign`	1.5.0	🙂	6s	7m 43s	48s	0.1 (0.0)	4.8 (0.0)	0.8 (0.0)	[7]	2020
`Uncalled4`	4.1.0	😄	15s	2m 23s	29s	3.5 (0.0)	15.9 (0.0)	8.0 (0.0)	[8]	2024
`Dynamont`	0.7.1	😄	8m 1s	6h 59m 16s	45m 52s	13.9 (0.0)	62.2 (0.0)	43.7 (0.0)	-	2025

precompiled binaries, with minimal setup overhead. `Dorado`, in particular, was straightforward to deploy, offering official binaries and comprehensive documentation on `GitHub`. However, several tools lacked compatibility with ONT's current `pod5` file format. In particular, `Tombo Resquiggle`, `f5c Resquiggle` and `f5c Eventalign` required conversion to other file formats, adding a preprocessing step. By contrast, `Uncalled4` and `Dynamont` support `pod5` directly, reducing preprocessing complexity and improving user experience. Execution commands are generally well documented. Especially tools requiring input format conversion, command-line execution was more complex but manageable with guidance provided in Sec. S2.1.

Runtime and Memory Efficiency.

Runtime and memory usage varied significantly across tools. `f5c Resquiggle` was the fastest, completing all data sets in under one minute and requiring at most 2.2 GB of RAM. `f5c Eventalign` had a longer runtime—up to 7 minutes and 43 seconds—and slightly higher memory usage. `Uncalled4` performed moderately in runtime but showed the highest peak memory usage at 15.9 GB. `Dorado`, while primarily a basecaller, exhibited longer runtimes (up to 11 minutes on a GPU-enabled system) due to the overhead of neural network inference. Nevertheless, it remained within reasonable limits for most workloads, utilizing 1.8 to 3.2 GB of RAM. `Dynamont`, implementing a full forward–backward HMM alignment without pre-filtering or event detection, was the most computationally intensive. It required up to 7 hours of runtime and higher memory consumption, trading efficiency for improved segmentation accuracy and flexibility. These trade-offs should be carefully weighed when choosing a segmentation tool depending on project size, available computational resources, and desired output fidelity.

For all evaluated algorithms, runtime and memory usage scale with both the number and length of input reads. Longer reads require more computation during segmentation, leading to a runtime that increases linearly with read length. Similarly, a larger number of reads proportionally extends total execution time. Memory usage also grows linearly, as all tools implement dynamic programming (DP) approaches—typically using banded matrices—where one dimension remains fixed relative to the input data. Another influence for the memory can be the number of processes used in parallel, e.g. `Dynamont` calculates multiple reads in parallel when using more than one process.

Discussion

The heatmap in Fig. 7 provides an overview of the aggregated performance of five segmentation tools and `Dorado` across twelve diverse data sets covering RNA002, RNA004, and DNA R10.4.1 5 kHz chemistries. Each cell in the matrix reflects the average normalized score across a range of metrics designed to capture segmentation quality, read retention, and downstream usability.

`Dorado` as a Baseline: High Compatibility but Limited Segmentation Precision

`Dorado`, while not a segmentation tool in the strictest sense, consistently achieves very high AM-scores across nearly all data sets, with an overall average score of 9.05. This is largely due to its role as the basecaller whose output serves as the input for all other tools. As such, it retains all reads without truncation and shows strong compatibility with downstream tools. However, as mentioned earlier, its segmentation metrics – particularly median delta and homogeneity – are considerably lower across all data sets. This underscores that `Dorado`, in the strictest sense, is not a segmentation tool. Its primary objective is to translate the ONT signal into nucleotide sequences, without explicitly modeling the assignment of individual nucleotides to signal segments, see Fig. 2. This is evident in the segmentation metrics and in Fig. 2, Tab. 2, and Tab. S1-S11, making it a less suitable candidate for direct signal interpretation."

`Tombo Resquiggle`: Limited Support for Modern Chemistries

`Tombo Resquiggle`, the oldest tool included, underperforms with an average score of 5.55. It ranks last in two of the four RNA002 data sets and, due to its deprecation, lacks support for modern chemistries. Its reliance on outdated formats and limited flexibility in signal handling significantly reduce its usability and performance.

`Tombo Resquiggle`'s median delta scores are higher than `Dorado` 's but lower than those of other segmentation tools across all RNA002 data sets. Its MAD delta consistently ranks high, Tab. S1-S4, achieving the best score in the SARS-CoV-2 data set, while its homogeneity score rarely surpasses that of other tools. The number of segmented and truncated reads is comparable to other segmentation tools, and it achieves the second shortest minimum read length. Overall, `Tombo Resquiggle` performs best in the MAD delta metric for the SARS-CoV-2 data set. `Tombo Resquiggle`'s comparatively low segmentation performance likely stems from its early development as one of the first segmentation tools released

in 2017 (Tab. 3). Subsequent tools have benefited from additional years of development, allowing for refinement of models, tuning of parameters, and building upon the foundational work introduced by `Tombo Resquiggle` to achieve improved segmentation of the ONT signal.

`f5c Resquiggle`: Efficient and Homogeneous Segmentation but Unsuitable for Short Reads

`f5c Resquiggle` ranks second among the segmentation tools, with an average score of 6.96, Fig.7, showing strong performance in read retention and moderate segmentation quality. It benefits from fast runtime and low memory usage, making it practical for large data sets. However, it relies on `Scrappie`'s event detection, which may limit its segmentation flexibility. `f5c Resquiggle` consistently achieves the highest homogeneity scores across all evaluated data sets and appears particularly effective at segmenting homogeneous regions of the signal.

Surprisingly, `f5c Resquiggle` appears to impose a lower bound on the minimum read length it segments. Across all evaluated data sets, the shortest segmented read consistently measures either 200 nt, resulting in a uniform score of 0.0 for this metric. We could not identify a command line parameter to modify this cutoff. This limitation has important implications: users working with short reads (<200 nt) should avoid using `f5c Resquiggle`, as it will systematically exclude these sequences from segmentation. Interestingly, `f5c Resquiggle` also shows a tendency to retain longer reads in DNA data sets more effectively than in RNA data sets, which could be caused by generally higher read qualities in ONT DNA sequencing.

`f5c Resquiggle` segments a smaller fraction of reads in RNA data sets compared to DNA data sets. While `f5c Resquiggle` consistently segments over 95 % of reads in all DNA data sets – with the remaining reads not segmented and effectively neglected—the segmentation rate for RNA varies widely, from as low as 16 % in the CEVd data set to 91 % in the RNA004 *H. sapiens* data set. This discrepancy is likely driven by differences in read quality and length, Tab. 1, which are influenced by the sequencing protocol, pore type, sample origin, and the presence of diverse RNA modifications in *in vivo* samples.

`f5c Eventalign`: High Precision Median Delta Segmentation with Mapping-Induced Read Loss

`f5c Eventalign`, a close relative of `f5c Resquiggle` that incorporates mapping, achieves a slightly lower overall score (6.36) and performs particularly poorly on data sets with high signal variation or mapping difficulty, such as SARS-CoV-2 and CEVd. Its reliance on reference-based alignment causes excessive filtering of long reads, impacting its read metrics and downstream compatibility. `f5c Eventalign` matches or surpasses `f5c Resquiggle`'s median delta score in 10 out of 12 data sets, being the overall best tool in that metric for the RNA004 CEVd and IVT data set. The MAD delta is only worse in the DNA Zymo HMW data set, otherwise either matching or surpassing `f5c Resquiggle`'s. Due to additional mapping, the number of segmented reads drops slightly compared to `f5c Resquiggle`. Although being related to `f5c Resquiggle`, `f5c Eventalign` is able to segment reads shorter than 200 nt, the shortest being 43 nt in the RNA004 CEVd data set. `f5c Eventalign` is not as good as `f5c Resquiggle` in segmenting the signal into homogeneous regions, as seen in the homogeneity metric, Tab. 2 and Tab. S1-S11. Compared to `f5c Resquiggle`, the maximum read length segmented by `f5c Eventalign` is even shorter, most probably caused by the mapping prior to segmentation, which can introduce additional read filtering and truncation.

`Uncalled4`: DTW-Based Segmentation with High Homogeneity in DNA but Read Length Limitations

`Uncalled4`, with an average score of 6.11, shows moderate performance. It outperforms `f5c Eventalign` in many RNA data sets and even leads in the DNA Zymo HMW data set. However, it too suffers from high memory usage and substantial read filtering due to its dependence on mapping. Interestingly, its segmentation quality (in terms of delta scores) rivals that of `f5c` tools, despite using a different core algorithm (DTW). `Uncalled4` has the highest median delta score for all RNA002 data sets. Within the four DNA data sets, the tool matches the highest homogeneity score of `f5c Resquiggle`. Overall, `Uncalled4` has comparable segmentation metric scores across all data sets. `Uncalled4` is in 9 out of 12 data sets able to segment more reads compared to `f5c Eventalign` and `f5c Resquiggle`. But, it reported max read length is among the shortest across the tools, scoring lowest in 6 data sets, and generally not being able to improve a lot compared to `f5c Eventalign` and `f5c Resquiggle`. This is possibly caused by the mapping and the similar event detection prior to segmentation.

Dynamont: Robust Full HMM Segmentation Outperforming Existing Tools Across Most Data Sets

`Dynamont` clearly stands out among the true segmentation tools. It achieves the highest overall average score (8.89) among the segmentation tools. It demonstrates its versatility by outperforming all tools in 7 out of 9 RNA data sets, often even surpassing Dorado, which sets a benchmark in terms of read and compatibility metrics. This confirms `Dynamont` 's robustness across organism/origins, signal characteristics, and sequencing kits. It consistently ranks first or second across 7 out of 8 RNA (RNA002 and RNA004) data sets, Tab. 2 and Tab S1-S7. Although `Dynamont` does not reach the highest AM-score in the DNA *H. sapiens* data set, the median delta is around 25 % and the MAD delta is 33 % higher compared to all other segmentation tools, Tab. S8. This improvement likely stems from skipping the event-detection and at the same time utilizing not only the forward pass, but also the backward pass of the HMM.

By avoiding enforced read filtering, `Dynamont` is able to segment nearly all reads in all data sets. It attempts to segment even very short or low-quality reads that other tools would typically filter. Through its use of a full forward-backward HMM pass, `Dynamont` not only takes on the challenge of segmenting these difficult reads, but also provides model-derived confidence probabilities for each segment. This enables users to make informed, data-driven decisions about whether to retain or exclude individual segments based on confidence scores. Allowing this level of control is particularly valuable when analyzing reads containing modifications, which often lead to basecalling errors and reduced quality.

`Dynamont`'s segmentation metrics on the Zymo HMW data set are notably lower compared to all other tools, a result that contrasts sharply with its otherwise consistently strong performance across data sets. Despite extensive analysis, we were unable to identify a definitive cause for this decline. One potential explanation could be the metagenomic nature of the Zymo data set, which may pose specific challenges. However, given that this conclusion is drawn from a single synthetic metagenomic data set, it remains speculative and warrants further investigation using a broader range of metagenomic samples. Its superior segmentation performance is attributed to its full forward–backward HMM implementation, which avoids signal pre-segmentation and mapping, allowing it to retain more reads, including very short, as well as very long ones, and to truncate less reads. Additionally, its ability to compute posterior probabilities enhances interpretability for

the user, Fig. 2, especially for difficult-to-segment signals like modified nucleotides or general basecalling errors.

Data Set Dependent Tool Performance: Short and Low Quality Reads

Tool performance varies across data sets. RNA004 data sets, especially CEVd and IVT, generally yield lower average scores, indicating that these samples present more challenging conditions for segmentation, likely due to shorter reads and lower quality, Tab. 1. The CEVd and IVT reference sequence is very short, which introduces additional difficulties in the `flye` assembly, Tab. S6 and Tab. S7. Segmentation tools, that require mapping prior to segmentation, show higher number of truncated and lower number of segmented reads, which also leads to difficulties in the `flye` assembly or the detection of structural variants with `svim`. In contrast, data sets such as DNA Zymo HMW and *P. anserina* consistently yield higher scores across tools, benefiting from higher read quality and length of the DNA sequencing protocol. In general, a trend can be suspected when comparing the scores of Fig. 7 with the read statistics of Tab. 1, the higher the quality and the longer the reads, the better the AM-score. Not only the AM-score is higher, also the raw segmentation metrics values are higher in the DNA results for all tools compared to RNA. DNA data sets with longer and better quality reads seem to be easier to be segmented than RNA reads.

Potential Implications

Here, we summarize some key findings for our comparative study: (a) No tools was best in all data sets. Depending on the users needs different tools are favorable above others. (b) Users working with short or low-quality RNA reads should avoid tools like `f5c Resquiggle`, which systematically exclude reads shorter than 200 nt. In such cases, `Dynamont` offers the best alternative, as it retains nearly all reads with minimal truncation and provides high segmentation accuracy. (c) Projects requiring accurate segmentation in homogeneous signal regions may benefit from using `f5c Resquiggle` due to its superior homogeneity scores, especially in DNA data sets. If accurate segmentation for heterogeneous regions is more favorable, than `Dynamont` with its consistently high median delta and MAD delta scores is recommendable. (d) For workflows emphasizing downstream applications (e.g., assembly, variant detection), `Dorado` offers strong baseline compatibility, but its low segmentation accuracy limits utility in modification detection or fine-grained signal analysis. (e) Tools like `f5c Eventalign` and `Uncalled4` 4, while promising, may underperform in data sets requiring robust long-read retention due to mapping-related filtering. (f) Memory and runtime considerations become critical for large-scale studies. `Dynamont`, while delivering the most accurate results, demands substantially more memory and computational time, which may limit its usability in high-throughput pipelines unless paired with adequate hardware. (g) `Dynamont` introduces a new benchmark in ONT signal segmentation but highlights the classic trade-off between performance and efficiency.

Limitations and Future Work

Despite the breadth and depth of our benchmarking study, several limitations must be acknowledged, and they offer opportunities for future work.

In this study, we evaluated each segmentation tool using its default configuration or minimally adapted parameters to ensure fairness and reproducibility. However, tool performance may vary significantly under different parameter settings, particularly when fine-tuning segmentation thresholds or adjusting for read quality. We encourage future studies to investigate the effect of broader parameter sweeps and optimization techniques, ideally tailored to specific data sets or organisms. Similarly, the requirement for read-to-reference mapping prior to segmentation may also influence performance and should be systematically evaluated. While our metrics aim to quantify segmentation performance, access to a ground truth signal segmentation would provide a more definitive and meaningful evaluation.

Data Set and Chemistry Scope

While our evaluation spans twelve data sets across three ONT chemistries (RNA002, RNA004, and DNA R10.4.1), it still represents only a subset of the growing diversity of sequencing protocols and pore types. Future work could extend this comparison by including additional pore chemistries (e.g., R10.4.2 or future releases), newer RNA sequencing kits, and broader organismal diversity, including metagenomic and single-cell data sets.

Tool and Metric Expansion

As new tools emerge, our comparison inevitably becomes temporally constrained. We provide a detailed Electronic Supplement that can be dynamically extended with new segmentation tools, metrics, and data sets in future iterations of this work. This modular approach will support continuous benchmarking and allow researchers to adapt the framework to their specific needs.

Integration into Downstream Workflows

Ultimately, segmentation tools serve as preprocessing stages for downstream analyses such as RNA modification detection, transcript isoform mapping, or structural variant calling. Future work should evaluate how segmentation quality influences these downstream tasks in a quantitative and biologically meaningful way. Integrating segmentation performance directly into end-to-end pipelines will help determine the practical utility of each method in real-world research applications.

Towards Ensemble Segmentation

Our results reveal complementary strengths among the segmentation tools. For example, while `f5c Resquiggle` achieves the best homogeneity, `Dynamont` shows superior overall robustness and delta metrics. This suggests a potential benefit in combining tool outputs into an ensemble segmentation approach—similar to ensemble methods in genome assembly or variant calling. Future research could explore consensus segmentation pipelines that select the best segments from each tool or fuse segmentations probabilistically based on alignment confidence and signal characteristics.

Standardization and Ground Truth

A key limitation in ONT signal segmentation remains the absence of a validated ground truth. This restricts evaluations to proxy metrics like segmentation similarity, read retention, and downstream compatibility. The development of simulated or experimentally validated benchmark data sets with known signal-to-sequence mappings would greatly enhance tool assessment. We advocate for a community-driven effort to define such standards.

Conclusion

In conclusion, while this study establishes a foundational benchmark for ONT segmentation tools, it also highlights the dynamic nature of this field. Continued benchmarking, tool refinement, and method integration are essential to ensure robust and accurate interpretation of nanopore sequencing signals.

`Dynamont` offers a robust approach for nanopore signal segmentation and alignment, particularly excelling in challenging conditions such as short or low-quality reads. Although `Dynamont` incurs a longer runtime and higher memory usage compared to existing tools, these costs are offset by its ability to segment significantly more reads and provide additional confidence probabilities for each segment. This is an innovation that cannot be found in other methods. These confidence scores, which quantify the reliability of each aligned k-mer and signal segment, are invaluable for downstream analyzes, including modification studies, error rate assessment, and source attribution of basecalling errors.

`Dynamont` achieves a higher or comparable segmentation scores in independent data sets across DNA and RNA chemistries, while maintaining similar segmentation homogeneity. Furthermore, `Dynamont` incorporates a more complex algorithm for error correction (see Sec. S2.5) and offers functionality for training custom k-mer models for specific data sets (see Sec. S2.6), providing flexibility for customized analyses.

Future development includes the reduction of runtime and memory complexity, which could be achieved by incorporating a pre-segmentation step to reduce runtime and memory complexity. This enhancement could diverge from the current `Scrappie`-based event detection method used by other tools, further optimizing `Dynamont` for large-scale and real-time applications. Overall, the unique features of `Dynamont`, its comprehensive segmentation coverage, confidence scoring, and error correction capabilities, position it as a powerful tool for advanced nanopore signal analysis.

Code Availability

`Dynamont`'s code is available via GitHub, Zenodo, `Conda`, and Pypi

Data Availability

The `pod5` files containing the 10,000 randomly selected reads for each data set and our reference sequences are available via Zenodo record 15011476.

Acknowledgements

We acknowledge funding by the DFG EXC 2051 – Project-ID 390713860 (KP), SFB1076/3.A06 (MM, JS); BMBF 01GR2305B.TP7 (MM); TMWWDG FKZ5575/10-9 (JS); and the European Research Council (CoG-101088027).

Competing Interest

The authors declare that they have no competing interests.

Use of AI-writing tools

The authors used ChatGPT to assist in improving the clarity, readability, and flow of the manuscript text. The AI tool was employed solely for language refinement and did not influence the empirical data, analyses, or conclusions of the study.

References

1. Jain M, Olsen HE, Paten B, Akeson M. The Oxford Nanopore MinION: delivery of nanopore sequencing to the genomics community. Genome Biology 2016 Nov;17(1). http://dx.doi.org/10.1186/s13059-016-1103-0.
2. Wang Y, Zhao Y, Bollas A, Wang Y, Au KF. Nanopore sequencing technology, bioinformatics and applications. Nature Biotechnology 2021 Nov;39(11):1348–1365. http://dx.doi.org/10.1038/s41587-021-01108-x.
3. Garalde DR, Snell EA, Jachimowicz D, Sipos B, Lloyd JH, Bruce M, et al. Highly parallel direct RNA sequencing on an array of nanopores. Nature Methods 2018 Jan;15(3):201–206. http://dx.doi.org/10.1038/nmeth.4577.
4. Liu-Wei W, van der Toorn W, Bohn P, Hölzer M, Smyth RP, von Kleist M. Sequencing accuracy and systematic errors of nanopore direct RNA sequencing. BMC Genomics 2024 May;25(1). http://dx.doi.org/10.1186/s12864-024-10440-w.
5. Cappannini A, Ray A, Purta E, Mukherjee S, Boccaletto P, Moafinejad SN, et al. MODOMICS: a database of RNA modifications and related information. 2023 update. Nucleic Acids Research 2023 Nov;52(D1):D239–D244. http://dx.doi.org/10.1093/nar/gkad1083.
6. Delahaye C, Nicolas J. Sequencing DNA with nanopores: Troubles and biases. PLOS ONE 2021 Oct;16(10):e0257521. http://dx.doi.org/10.1371/journal.pone.0257521.
7. Gamaarachchi H, Lam CW, Jayatilaka G, Samarakoon H, Simpson JT, Smith MA, et al. GPU accelerated adaptive banded event alignment for rapid comparative nanopore signal analysis. BMC bioinformatics 2020;21(1):1–13.
8. Kovaka S, Hook PW, Jenike KM, Shivakumar V, Morina LB, Razaghi R, et al. Uncalled4 improves nanopore DNA and RNA modification detection via fast and accurate signal alignment. Nature Methods 2025 Mar;22(4):681–691. http://dx.doi.org/10.1038/s41592-025-02631-4.
9. Pratanwanich PN, Yao F, Chen Y, Koh CWQ, Wan YK, Hendra C, et al. Identification of differential RNA modifications from nanopore direct RNA sequencing with xPore. Nature Biotechnology 2021 Jul;39(11):1394–1402. http://dx.doi.org/10.1038/s41587-021-00949-w.
10. Spangenberg J, zu Siederdissen CH, Žarković M, Triebel S, Rose R, Christophersen CM, et al. Magnipore: Prediction of differential single nucleotide changes in the Oxford Nanopore Technologies sequencing signal of SARS-CoV-2 samples 2023 Mar;http://dx.doi.org/10.1101/2023.03.17.533105.
11. Watson KJ, Bromley RE, Dunning Hotopp JC. Duplexed direct RNA sequencing protocol using polyadenylation and polyuridylation. Microbiology Resource Announcements 2025 Jan;http://dx.doi.org/10.1128/mra.01041-24.
12. Vopalensky P, Škríba A, Chiumenti M, Ďuričeková L, Šimonová A, Lukšan O, et al. Exploring RNA modifications in infectious non-coding circular RNAs. RNA Biology 2025 Feb;22(1):1–9. http://dx.doi.org/10.1080/15476286.2025.2459039.
13. Sereika M, Kirkegaard RH, Karst SM, Michaelsen TY, Sørensen EA, Wollenberg RD, et al. Oxford Nanopore R10.4 long-read sequencing enables the generation of near-finished bacterial genomes from pure cultures and metagenomes without short-read or reference polishing. Nature Methods 2022 Jul;19(7):823–826. http://dx.doi.org/10.1038/s41592-022-01539-7.
14. Dabernig-Heinz J, Lohde M, Hölzer M, Cabal A, Conzemius R, Brandt C, et al. A multicenter study on accuracy and reproducibility of nanopore sequencing-based genotyping of bacterial pathogens. Journal of Clinical Microbiology 2024 Sep;62(9). http://dx.doi.org/10.1128/jcm.00628-24.
15. Ament-Velásquez SL, Furneaux B, Dheur S, Granger-Farbos A, Stelkens R, Johannesson H, et al., Reconstructing NOD-like receptor alleles with high internal conservation in Podospora anserina using long-read sequencing. Dryad; 2025. https://datadryad.org/dataset/doi:10.5061/dryad.h18931zww.
16. Raiber EA, Hardisty R, van Delft P, Balasubramanian S. Mapping and elucidating the function of modified bases in DNA. Nature Reviews Chemistry 2017;1(9):0069.
17. Loman NJ, Quick J, Simpson JT. A complete bacterial genome assembled de novo using only nanopore sequencing data. Nature Methods 2015 Jun;12(8):733–735. https://doi.org/10.1038/nmeth.3444.
18. Rabiner LR. A tutorial on hidden Markov models and selected applications in speech recognition. Proceedings of the IEEE 1989;77(2):257–286. http://dx.doi.org/10.1109/5.18626.
19. Durbin R, Eddy SR, Krogh A, Mitchison G. Biological Sequence Analysis: Probabilistic Models of Proteins and Nucleic Acids. Cambridge University Press; 1998. http://dx.doi.org/10.1017/CBO9780511790492.
20. Kolmogorov M, Bickhart DM, Behsaz B, Gurevich A, Rayko M, Shin SB, et al. metaFlye: scalable long-read metagenome assembly using repeat graphs. Nature Methods 2020 Oct;17(11):1103–1110. http://dx.doi.org/10.1038/s41592-020-00971-x.
21. Heller D, Vingron M. SVIM: structural variant identification using mapped long reads. Bioinformatics 2019 Jan;35(17):2907–2915. http://dx.doi.org/10.1093/bioinformatics/btz041.
22. Samarakoon H, Ferguson JM, Jenner SP, Amos TG, Parameswaran S, Gamaarachchi H, et al. Flexible and efficient handling of nanopore sequencing signal data with slow5tools. Genome Biology 2023 Apr;24(1). http://dx.doi.org/10.1186/s13059-023-02910-3.
23. Pearson RK, Neuvo Y, Astola J, Gabbouj M. Generalized Hampel Filters. EURASIP Journal on Advances in Signal Processing 2016 Aug;2016(1). http://dx.doi.org/10.1186/s13634-016-0383-6.
24. Gales M, Young S. The Application of Hidden Markov Models in Speech Recognition. Foundations and Trends® in Signal Processing 2007;1(3):195–304. http://dx.doi.org/10.1561/2000000004.
25. Juang BH, Levinson S, Sondhi M. Maximum likelihood estimation for multivariate mixture observations of markov chains (Corresp.). IEEE Transactions on Information Theory 1986;32(2):307–309.

S2 Supplemental Material

S2.1 Commands

S2.1.1 Benchmark Preparation Commands

```
# Extract 10.000 Random Reads
pod5 view <dataset.pod5> --ids --no-header -o all_ids.txt
sort --random-sort all_ids.txt | head --lines 10000 > 10k_ids.txt
pod5 filter <dataset.pod5> -o <dataset_r10k.pod5> --ids 10k_ids.txt
# Convert To Other Data Formats
blue-crab p2s <dataset_r10k.pod5> -o <dataset_r10k.blow5>
pod5 convert to_fast5 <dataset_r10k.pod5> -o fast5/
multi_to_single_fast5 -i fast5/ -s single_fast5/
# Basecalling
## explicitely using rna002_70bps_hac\@v3 for RNA002 data
dorado basecaller sup -x cuda:0 <dataset_r10k.pod5> > <dataset_r10k.bam>
samtools bam2fq <dataset_r10k.bam> > <dataset_r10k.fastq>
dorado summary <dataset_r10k.bam> > sequencing_summary.txt
# converting sequencing summary to tombo format (single fast5)
awk -F'\t' 'NR == 1 {print; next} {$1 = $2 ".fast5"; print}' OFS='\t' sequencing_summary.txt > tombo_sequencing_summary.txt
# Mapping
## preset = splice if RNA and h_sapiens, s_cerevisiae, e_coli, sarscov2
## preset = lr:hq for DNA R10.4.1
## preset = map-ont else
minimap2 <ref.fa> <dataset_r10k.fastq> -x <preset> -a | samtools view -hbF4 | samtools sort > <dataset_r10k_mapping.bam>
samtools index <dataset_r10k_mapping.bam>
```

S2.1.2 Dynamont Segmentation Commands

```
# model can be added explicitely, otherwise default pore model is chosen
python segment.py --raw <path/to/pod5/dataset_r10k/> --basecalls <dataset_r10k.bam> --mode basic -o <dynamont.csv> --pore <pore>
```

S2.1.3 Dorado Segmentation Commands

```
# Basecalling with emit moves
## explicitely using rna002_70bps_hac\@v3 for RNA002 data
dorado basecaller sup -x cuda:0 --emit-moves <dataset_r10k.pod5> > <dataset_r10k_moves.bam>
# Extracting moves as segmentation borders
python extractDoradoMoves.py <dataset_r10k_moves.bam> -o <dataset_r10k_moves.tsv>
```

S2.1.4 f5c Segmentation Commands

```
f5c index --slow5 <dataset_r10k.blow5> <dataset_r10k.fastq>
# Eventalign
## added --rna in case of RNA
f5c Eventalign -b <dataset_r10k_mapping.bam> -g <ref.fa> -r <dataset_r10k.fastq> --slow5 <dataset_r10k.blow5> --signal-index --collapse-events --pore <pore> --min-mapq 0 --summary <dataset_r10k_event.sum> > <dataset_r10k_event.tsv>
# Resquiggle
## added --rna in case of RNA
f5c Resquiggle --pore <pore> <dataset_r10k.fastq> <dataset_r10k.blow5> > <dataset_r10k_resqu.tsv>
```

S2.1.5 Tombo Segmentation Commands

```
tombo preprocess annotate_raw_with_fastqs --fast5-basedir single_fast5/ --fastq-filenames <dataset_r10k.fastq> --sequencing-summary-filenames sequencing_summary.txt
# only executed on RNA002
tombo Resquiggle --q-score 0 --rna single_fast5/ <ref.fa>
```

S2.1.6 Uncalled4 Segmentation Commands

```
# preset = splice if RNA and h_sapiens, s_cerevisiae, e_coli, sarscov2
# preset = lr:hq for DNA R10.4.1
# preset = map-ont else
dorado basecaller sup -x cuda:0 --reference <ref.fa> --mm2-opts "-x <preset> --secondary=no" --emit-moves <dataset_r10k.pod5> > <mapped_basecalls.bam>
samtools view -hbF 2304 <mapped_basecalls.bam> > <primary_mapped_basecalls.bam>
uncalled4 align --ref <ref.fa> --reads <dataset_r10k.pod5> --bam-in <primary_mapped_basecalls.bam> --tsv-out <uncalled4_segmentation.tsv> --tsv-cols aln.read_id,dtw --min-aln-length 1
```

S2.2 Normalizing the ONT Signal

All signals are provided in `fast5` or `pod5` (from ONT), or in `slow5` [22] format. Within these formats, they are stored as integer values T_{DACs} to reduce memory space. They can be converted to pA T_{pA} using Equ. 5. The required parameters are also for each read. Each signal is normalized, Equ. 6 and Fig. S2, using the parameters provided by `Dorado`, the state-of-the-art basecaller from ONT. They are found in the `bam` output after basecalling. The shift parameter is stored in the `sm` tag (scaling midpoint) and the scale parameter in the `sd` tag (scaling dispersion). In case of the RNA002 chemistry, `sm` and `sd` are used to normalize the pA signal. For the new RNA004 chemistry, ONT changed the `sm` and `sd` parameter to normalize the raw integer values directly, skipping the conversion to the pA signal.

$$T_{\text{pA}} = \frac{(T_{\text{DACs}} + \text{offset}) * \text{range}}{\text{digitization}} \tag{5}$$

$$T_{\text{norm}} = \frac{T_{\text{pA}} - \text{shift}}{\text{scale}} \tag{6}$$

S2.3 Filtering Outliers

The signal T can contain errors, Fig. S2. They manifest as sudden peaks or drops within the signal. Which is why we apply a Hampel filter [23] to each signal. It removes outliers in time series data, by iterating with a sliding window approach. Within the window, each data point is compared to the window median. When a data point deviates from the median by more than a defined threshold, typically expressed as a multiple of the MAD, it is identified as an outlier. Each outlier is replaced by the window median. The Hampel filter is insensitive to extreme values, making it effective in handling non-Gaussian noise and preserving the integrity of the underlying signal. We use a window size of 6, and a deviation threshold of 5.0.

S2.4 Trimming Adapter

The `bam` file from `Dorado` contains additional tags[8]. The `ts` tag holds the number of trimmed signal data points (t) from the start of the signal, e.g. sequencing adapters, Fig. S2. Tag `ns` is the number of signal data points in the signal prior to trimming. During library preparation, multiple reads can get ligated, Fig. S2. Their concatenated signal will be split by `Dorado` into two reads. Split reads get assigned with a new read ID, different from the one of the signal. They have the additional `pi` tag, containing the signal ID and the `sp` tag marking the split position in the signal T. The normalized and trimmed signal T_{trimmed}, Equ. 7, and read N is passed to `Dynamont`.

$$T_{\text{trimmed}} = (t_{sp+ts}, \ldots, t_{sp+ns-1}) \tag{7}$$

For the sake of simplicity, the trimmed signal T_{trimmed} will be named T with the length $\mathcal{T}$ in the following text.

S2.5 Dynamont with Error Correction

`Dynamont` with error correction aligns N to T while doing the correction on an additional dimension C. It consists of 5 states: A (align), E (extend), I (insertion), S (sequence), and P (polish). A and E are similar to before. A moves in all dimensions, aligning t_i, k_j, and c_h. c_h is a k-mer of C with the index h and k_j is the k-mer of N at position j. c_h and k_j may differ. A starts a new segment for k_j and c_h at the same "time" t_i. E moves in T, while halting in N and C, extending the current segment. I handles the insertion of nucleotides in N, by moving only in N, while halting in T and C. P does the opposite, by moving in T and C it can handle deletions and additional polishings. S moves in N and T, while halting in C. While A allows a simultaneous segment start in all dimensions, P and S allow for different segment starts and ends of the N to T alignment versus the T to C alignment.

The forward algorithm α iterates N and T similar to `NT HMM` but the iteration of C is different. To move from one c to the next, $p(h)$ is introduced. This function returns the indices of preceding k-mers of

8 https://github.com/nanoporetech/bonito/blob/master/documentation/SAM.md

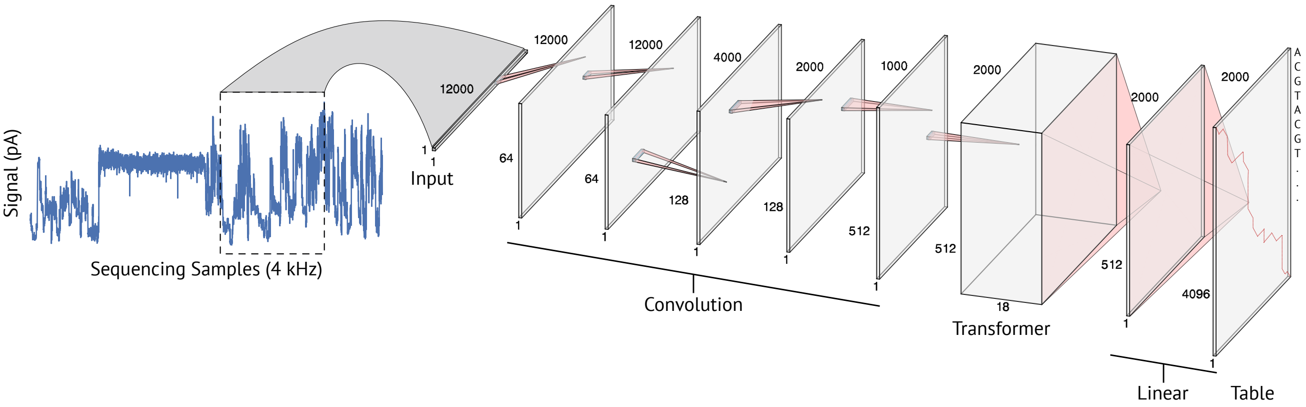

Figure S1. The `Dorado` RNA004 'super accuracy' basecalling model consists of five convolution layers, eighteen transformer layers, and two linear layers. The model outputs a table in the end for each input signal window of 12 000 samples. This table downsamples the 12 000 input signals to 2 000 and holds the models prediction for the nucleotide sequence (rows: 1024 times 4 bases). It can be decoded using algorithms like viterbi or beam search, which will yield the nucleotide sequence for that window. Overlapping nucleotide sequences from overlapping windows are merged later on.

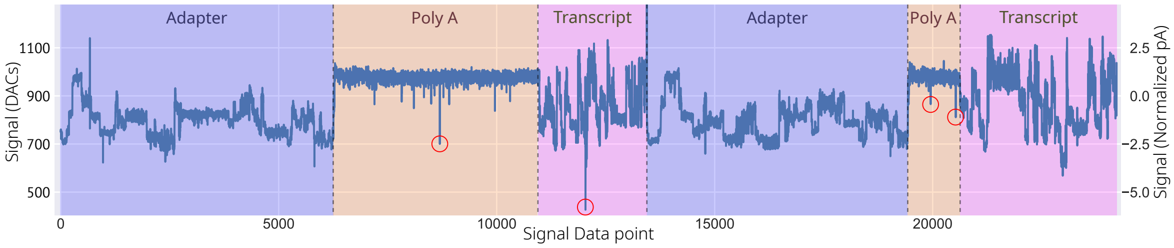

Figure S2. Preprocessing Overview of the Signal. The unnormalized time series (left y-axis) is normalized (right y-axis) to standardize the data. Detected outliers (red circles) mark points that deviate significantly from the expected signal pattern. These outliers can introduce very low probabilities in the HMM Forward-Backward algorithm. The time series presented here originates from a RNA read. It is sequenced from 3' to 5' end, divided into "Adapter," "Poly A," and "Transcript". A DNA signal would be sequenced from 5' to 3' end, contain a different adapter signal, no "Poly A" region, and a part that looks similar to the "Transcript" Within a single signal these segments can appear multiple times, caused by ligated reads or reads, that are sequenced nearly instantaneous after each other. `Dorado` will split these reads during basecalling.

c_h in C. For example, if c_h = AGGTC then $c_{p(h)}$ are AAGGT, CAGGT, GAGGT, and GAGGT, if Σ ={A, C, G, T}. All rules using $p(h)$ are called $|\Sigma|$ times.

$$
\begin{aligned}
A:&\begin{pmatrix}M_j\\M_i\\M_h\end{pmatrix}\rightarrow a1\begin{pmatrix}H_{j-1}\\M_{i-1}\\H_{p(h)}\end{pmatrix}\begin{bmatrix}k_j\\t_i\\c_h\end{bmatrix}\Bigg|\; a2\begin{pmatrix}M_{j-1}\\H_{i-1}\\H_{p(h)}\end{pmatrix}\begin{bmatrix}k_j\\t_i\\c_h\end{bmatrix}\Bigg|\Bigg|\begin{bmatrix}\epsilon_{j=0}\\\epsilon_{i=0}\\c_h\in C\end{bmatrix}\\
I:&\begin{pmatrix}M_j\\H_i\\H_h\end{pmatrix}\rightarrow i1\begin{pmatrix}H_{j-1}\\M_i\\H_h\end{pmatrix}\begin{bmatrix}k_j\\t_i\\c_h\end{bmatrix}\Bigg|\; i2\begin{pmatrix}M_{j-1}\\H_i\\H_h\end{pmatrix}\begin{bmatrix}k_j\\t_i\\c_h\end{bmatrix}\\
P:&\begin{pmatrix}H_j\\M_i\\M_h\end{pmatrix}\rightarrow p1\begin{pmatrix}M_j\\M_{i-1}\\H_{p(h)}\end{pmatrix}\begin{bmatrix}k_j\\t_i\\c_h\end{bmatrix}\Bigg|\; p2\begin{pmatrix}H_j\\M_{i-1}\\H_{p(h)}\end{pmatrix}\begin{bmatrix}k_j\\t_i\\c_h\end{bmatrix}\Bigg|\; p3\begin{pmatrix}M_j\\H_{i-1}\\H_{p(h)}\end{pmatrix}\begin{bmatrix}k_j\\t_i\\c_h\end{bmatrix}\\
S:&\begin{pmatrix}M_j\\M_i\\H_h\end{pmatrix}\rightarrow s1\begin{pmatrix}H_{j-1}\\M_{i-1}\\M_h\end{pmatrix}\begin{bmatrix}k_j\\t_i\\c_h\end{bmatrix}\Bigg|\; s2\begin{pmatrix}H_{j-1}\\M_{i-1}\\H_h\end{pmatrix}\begin{bmatrix}k_j\\t_i\\c_h\end{bmatrix}\Bigg|\; s3\begin{pmatrix}M_{j-1}\\H_{i-1}\\H_h\end{pmatrix}\begin{bmatrix}k_j\\t_i\\c_h\end{bmatrix}\\
E:&\begin{pmatrix}H_j\\M_i\\H_h\end{pmatrix}\rightarrow e1\begin{pmatrix}M_j\\M_{i-1}\\M_h\end{pmatrix}\begin{bmatrix}k_j\\t_i\\c_h\end{bmatrix}\Bigg|\; e2\begin{pmatrix}H_j\\M_{i-1}\\M_h\end{pmatrix}\begin{bmatrix}k_j\\t_i\\c_h\end{bmatrix}\Bigg|\; e3\begin{pmatrix}M_j\\M_{i-1}\\H_h\end{pmatrix}\begin{bmatrix}k_j\\t_i\\c_h\end{bmatrix}\Bigg|\; e4\begin{pmatrix}H_j\\M_{i-1}\\H_h\end{pmatrix}\begin{bmatrix}k_j\\t_i\\c_h\end{bmatrix}
\end{aligned}
\tag{8}
$$

As the alignment is not limited in C, the final alignment score Z is not found at a specific index, but as a sum over the last column:

$$Z = \sum_{c_h \in C} E_{\mathcal{N}-k,\mathcal{T}-1,h}$$

The backward algorithm β iterates, backwards over the indices. Here $s(h)$ is the inverse function of $p(h)$. $s(h)$ returns the indices in C of the succeeding k-mers of c_h, e.g. if c_h = AGGTC, the succeeding k-mers are GGTCA, GGTCC, GGTCG, and GGTCT.

$$
\begin{aligned}
A^*:&\begin{pmatrix}M_j\\M_i\\M_h\end{pmatrix}\rightarrow e1\begin{pmatrix}H_j\\M_{i+1}\\H_h\end{pmatrix}\begin{bmatrix}k_j\\t_{i+1}\\c_h\end{bmatrix}\\
I^*:&\begin{pmatrix}M_j\\H_i\\H_h\end{pmatrix}\rightarrow a2\begin{pmatrix}M_{j+1}\\M_{i+1}\\M_{s(h)}\end{pmatrix}\begin{bmatrix}k_{j+1}\\t_{i+1}\\c_{s(h)}\end{bmatrix}\Bigg|\; i2\begin{pmatrix}M_{j+1}\\H_i\\H_h\end{pmatrix}\begin{bmatrix}k_{j+1}\\t_i\\c_h\end{bmatrix}\Bigg|\\
&\qquad p3\begin{pmatrix}H_j\\M_{i+1}\\M_{s(h)}\end{pmatrix}\begin{bmatrix}k_j\\t_{i+1}\\c_{s(h)}\end{bmatrix}\Bigg|\; s3\begin{pmatrix}M_{j+1}\\M_{i+1}\\H_h\end{pmatrix}\begin{bmatrix}k_{j+1}\\t_{i+1}\\c_h\end{bmatrix}\\
P^*:&\begin{pmatrix}H_j\\M_i\\M_h\end{pmatrix}\rightarrow s1\begin{pmatrix}M_{j+1}\\M_{i+1}\\H_h\end{pmatrix}\begin{bmatrix}k_{j+1}\\t_{i+1}\\c_h\end{bmatrix}\Bigg|\; e2\begin{pmatrix}H_j\\M_{i+1}\\H_h\end{pmatrix}\begin{bmatrix}k_j\\t_{i+1}\\c_h\end{bmatrix}\\
S^*:&\begin{pmatrix}M_j\\M_i\\H_h\end{pmatrix}\rightarrow p1\begin{pmatrix}H_j\\M_{i+1}\\M_{s(h)}\end{pmatrix}\begin{bmatrix}k_j\\t_{i+1}\\c_{s(h)}\end{bmatrix}\Bigg|\; e3\begin{pmatrix}H_j\\M_{i+1}\\H_h\end{pmatrix}\begin{bmatrix}k_j\\t_{i+1}\\c_h\end{bmatrix}\\
E^*:&\begin{pmatrix}H_j\\M_i\\H_h\end{pmatrix}\rightarrow a1\begin{pmatrix}M_{j+1}\\M_{i+1}\\M_{s(h)}\end{pmatrix}\begin{bmatrix}k_{j+1}\\t_{i+1}\\c_{s(h)}\end{bmatrix}\Bigg|\; i1\begin{pmatrix}M_{j+1}\\H_i\\H_h\end{pmatrix}\begin{bmatrix}k_{j+1}\\t_i\\c_h\end{bmatrix}\Bigg|\; p2\begin{pmatrix}H_j\\M_{i+1}\\M_{s(h)}\end{pmatrix}\begin{bmatrix}k_j\\t_{i+1}\\c_{s(h)}\end{bmatrix}\Bigg|\\
&\qquad s2\begin{pmatrix}M_{j+1}\\M_{i+1}\\H_h\end{pmatrix}\begin{bmatrix}k_{j+1}\\t_{i+1}\\c_h\end{bmatrix}\Bigg|\; e4\begin{pmatrix}H_j\\M_{i+1}\\H_h\end{pmatrix}\begin{bmatrix}k_j\\t_{i+1}\\c_h\end{bmatrix}\Bigg|\Bigg|\begin{bmatrix}\delta^*_{j=\mathcal{N}-k}\\\delta^*_{i=\mathcal{T}-1}\\c_h\in C\end{bmatrix}
\end{aligned}
\tag{9}
$$

The alignment score Z^* is calculated by:

$$Z^* = \sum_{c_h \in C} A_{0,0,h}$$

Analogous to `Dynamont`s basic algorithm, the runtime and memory complexity for the error correction is $O(\mathcal{B}\mathcal{T}|C|)$.

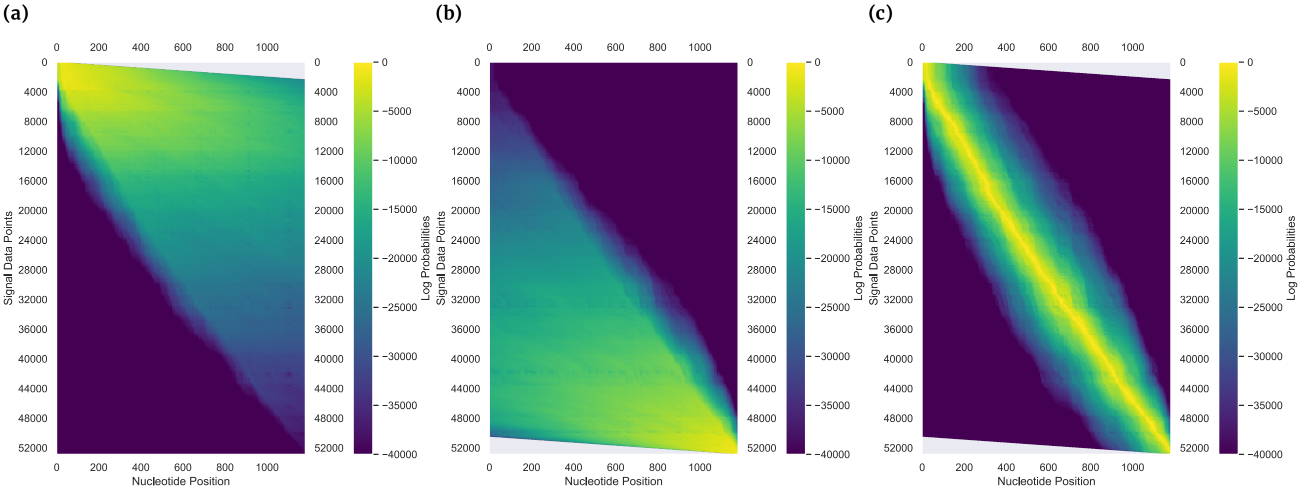

Figure S3. Logarithmic probabilities of full ($N \times T$) `Dynamont` matrices, calculated during a) forward pass; b) backward pass; and c) the combined posterior probability. The matrices show the probability flux of the nucleotide sequence (N) to ONT signal (T) alignment, using the HMM implemented in `Dynamont`. The highest probability can be found around the diagonal, which can be used for further optimizations.

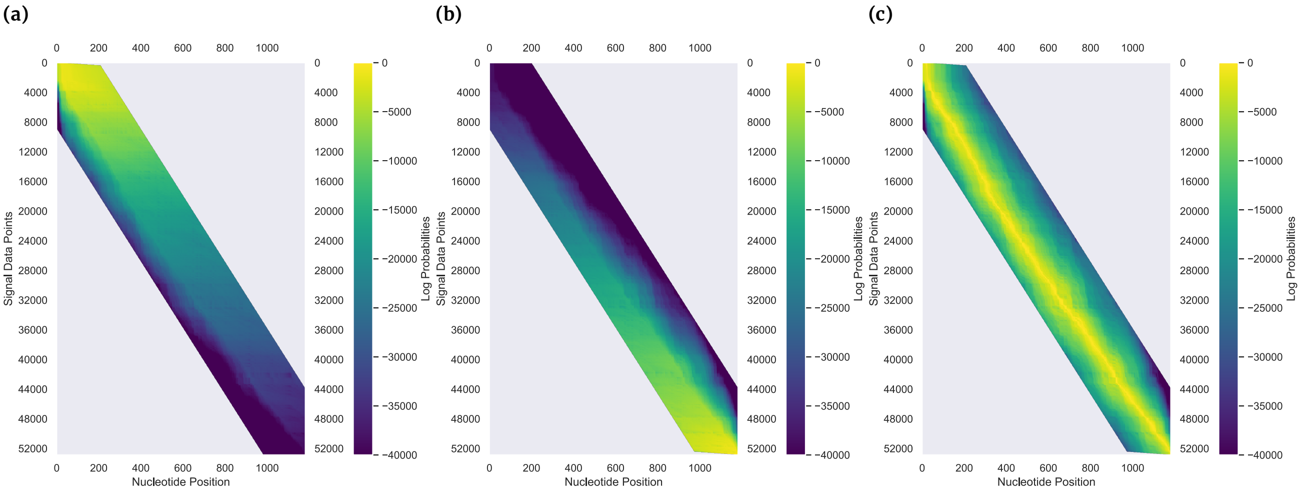

Figure S4. Logarithmic probabilities of banded DP ($N \times T$) `Dynamont` matrices, calculated during a) forward pass; b) backward pass; and c) the combined posterior probability. The matrices show the probability flux of the nucleotide sequence (N) to ONT signal (T) alignment, using the HMM implemented in `Dynamont`. The highest probability can be found around the diagonal, which can be used for further optimizations.

S2.6 Training Parameters with the Baum-Welch Algorithm

Using the Forward-Backward algorithm, we can utilize the Baum-Welch algorithm [18, 19] to train our parameters. It is a special case of the Expectation Maximization Algorithm to find unknown transition and emission parameters of an HMM.

S2.6.1 *Training transition parameters*

The transition parameters are trained by calculating how many times they were used given a training pair of T and N. Given the states q and r, a_{qr} is the transition probability from q to r. The new transition $\hat{a}_{qr}$ is calculated with Equ. 10.

$$A_{qr} = \frac{\sum_x \alpha_q(x) a_{qr} \phi(o_{x+1}) \beta_r(x+1)}{Z}$$
$$\hat{a}_{qr} = \frac{A_{qr}}{\sum_{r'} A_{qr'}} \tag{10}$$

$\sum_x$ iterates the observations o, in our case k_j and t_i. $\alpha_q(x)$ is the score in state q in α at x. $\beta_r(x+1)$ is the score of state r in β when taking a_{qr} to $x+1$. a_{qr} is the transition probability for the path from $\alpha_q(x)$ to $\beta_r(x+1)$. $\phi(o_{x+1})$ is the emission for observing o_{x+1}. Each transition probability needs to be normalized afterwards to ensure that all outgoing transitions sum up to 1.

S2.6.2 *Training emission parameters*

New model parameters $\hat{\mu}_k$ and $\hat{\sigma}_k$ for the Gaussian distributions are trained with the Baum-Welch algorithm [24, 25]. First, the expectation step calculates weights for all observations, Equ. 11. For each i and j, the sum of scores of all matrices Q in α get multiplied with the sum of scores of all matrices Q^* in β, which is standardized by Z. γ is a matrix that stores these weights for all i and j.

$$\gamma_{i,j} = \frac{\sum_Q Q_{i,j} \sum_{Q^*} Q^*_{i,j}}{Z} \tag{11}$$

Within the maximization step, Equ. 12, the weights in γ are used to calculate a weighted sum of the observations t_i, which will be standardized with the sum of weights over i of γ, to get $\hat{\mu}_j$. As a k-mer can appear multiple times in N, $\hat{\mu}_k$ is calculated by taking the average $\hat{\mu}_j$ for all $k_j = k$ in N. The same happens for $\hat{\sigma}_k$.

$$\hat{\mu}_j = \frac{\sum_{i=1}^{\mathcal{T}-1} \gamma_{i,j} t_i}{\sum_{i=1}^{\mathcal{T}-1} \gamma_{i,j}} \qquad \hat{\sigma}_j^2 = \frac{\sum_{i=1}^{\mathcal{T}-1} \gamma_{i,j} (t_i - \hat{\mu}_j)^2}{\sum_{i=1}^{\mathcal{T}-1} \gamma_{i,j}}$$
$$\hat{\mu}_k = \frac{\sum_{\forall j: k_j = k} \hat{\mu}_j}{\text{counts}_k(N)} \qquad \hat{\sigma}_k = \sqrt{\frac{\sum_{\forall j: k_j = k} \hat{\sigma}_j^2}{\text{counts}_k(N)}} \tag{12}$$

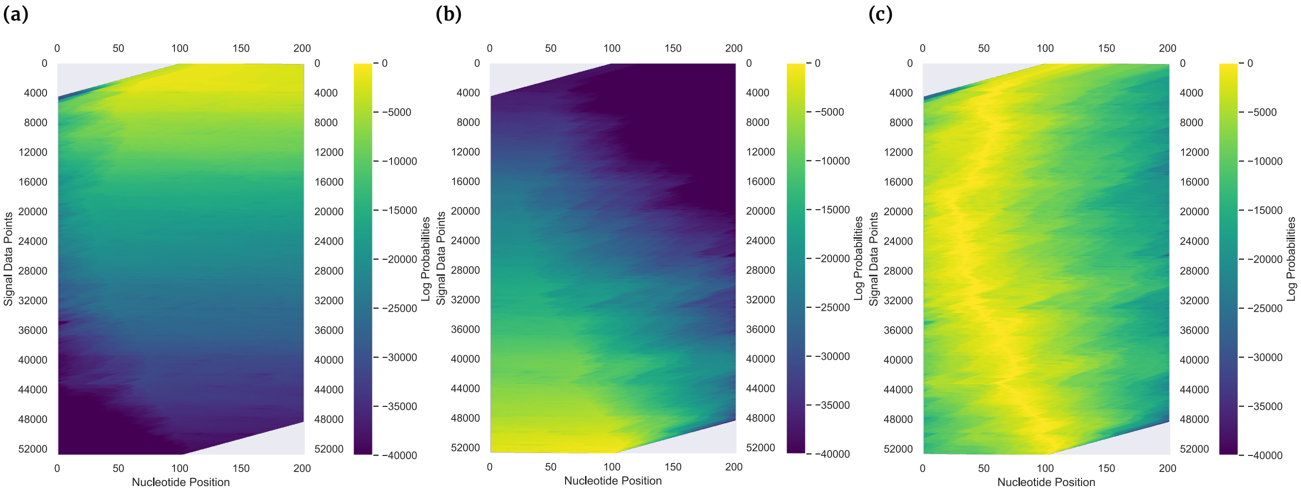

Figure S5. Logarithmic probabilities of reduced banded DP ($N \times T$) `Dynamont` matrices, calculated during a) forward pass; b) backward pass; and c) the combined posterior probability. The matrices show the probability flux of the nucleotide sequence (N) to ONT signal (T) alignment, using the HMM implemented in `Dynamont`. The highest probability can be found around the diagonal, which can be used for further optimizations.

Table S1. Metrics for *H. sapiens* RNA002 dataset.

Tool	Dorado	Tombo	f5c R.	f5c E.	Uncalled4	Dynamont
median delta ($\Delta\mu$)	0.240 18.5	0.740 57.0	0.916 70.5	0.961 74.0	1.000 77.0	0.942 72.5
mad delta ($\Delta\sigma$)	0.875 7.0	0.938 7.5	0.750 6.0	0.750 6.0	0.750 6.0	1.000 8.0
homogeneity	0.000 17.0	0.118 15.0	0.176 14.0	0.176 14.0	0.176 14.0	0.118 15.0
segmented reads	1.000 10873.0	0.796 8656.0	0.805 8750.0	0.770 8370.0	0.896 9738.0	1.000 10873.0
truncated reads	1.000 0.0	0.288 7742.0	0.195 8750.0	0.234 8329.0	0.150 9241.0	0.000 10873.0
min read length	0.975 5.0	0.770 46.0	0.000 200.0	0.660 68.0	0.625 75.0	0.995 1.0
n50 read length	0.991 1122.0	0.966 1093.0	1.000 1132.0	0.938 1062.0	0.946 1071.0	0.979 1108.0
max read length	1.000 21695.0	0.333 7216.0	0.335 7273.0	0.320 6934.0	0.332 7208.0	1.000 21691.0
flye total length	0.916 68389.0	0.841 62783.0	0.773 57706.0	0.651 48630.0	0.972 72551.0	1.000 74650.0
flye n50	0.854 2454.0	0.923 2653.0	0.873 2511.0	1.000 2875.0	0.873 2510.0	0.862 2477.0
flye mean coverage	1.000 6.5	0.831 5.4	0.947 6.2	0.882 5.8	0.878 5.7	0.961 6.3
svim structural variants	0.828 130.0	0.000 0.0	1.000 157.0	0.987 155.0	0.108 17.0	0.847 133.0
aggregated metric score	9.68	7.54	7.77	8.33	7.71	9.70

Table S2. Metrics for *E. coli* RNA002 dataset.

Tool	Dorado	Tombo	f5c R.	f5c E.	Uncalled4	Dynamont
median delta ($\Delta\mu$)	0.248 19.5	0.783 61.5	0.866 68.0	0.936 73.5	1.000 78.5	0.955 75.0
mad delta ($\Delta\sigma$)	0.882 7.5	0.941 8.0	0.706 6.0	0.765 6.5	0.765 6.5	1.000 8.5
homogeneity	0.000 18.0	0.139 15.5	0.222 14.0	0.167 15.0	0.167 15.0	0.167 15.0
segmented reads	1.000 10717.0	0.170 1821.0	0.242 2593.0	0.145 1559.0	0.183 1959.0	1.000 10717.0
truncated reads	1.000 0.0	0.324 1754.0	0.000 2593.0	0.399 1558.0	0.253 1938.0	0.953 121.0
min read length	0.975 5.0	0.780 44.0	0.000 200.0	0.700 60.0	0.640 72.0	0.950 10.0
n50 read length	0.645 398.0	0.765 472.0	1.000 617.0	0.900 555.0	0.754 465.0	0.624 385.0
max read length	1.000 31807.0	0.075 2372.0	0.099 3163.0	0.084 2675.0	0.074 2364.0	1.000 31812.0
flye total length	0.918 5093.0	0.000 0.0	1.000 5550.0	0.000 0.0	0.000 0.0	0.919 5098.0
flye n50	0.912 2648.0	0.000 0.0	1.000 2903.0	0.000 0.0	0.000 0.0	0.914 2653.0
flye mean coverage	1.000 17.0	0.000 0.0	0.647 11.0	0.000 0.0	0.000 0.0	0.971 16.5
svim structural variants	0.710 44.0	0.000 0.0	0.742 46.0	1.000 62.0	0.000 0.0	0.774 48.0
aggregated metric score	9.29	3.98	6.52	5.10	3.83	10.23

Table S3. Metrics for SARS-CoV-2 RNA002 dataset.

Tool	Dorado	Tombo	f5c R.	f5c E.	Uncalled4	Dynamont
median delta ($\Delta\mu$)	0.248 $_{18.5}$	0.718 $_{53.5}$	0.906 $_{67.5}$	0.899 $_{67.0}$	1.000 $_{74.5}$	0.960 $_{71.5}$
mad delta ($\Delta\sigma$)	0.933 $_{7.0}$	1.000 $_{7.5}$	0.733 $_{5.5}$	0.800 $_{6.0}$	0.800 $_{6.0}$	1.000 $_{7.5}$
homogeneity	0.000 $_{16.5}$	0.152 $_{14.0}$	0.212 $_{13.0}$	0.182 $_{13.5}$	0.152 $_{14.0}$	0.152 $_{14.0}$
segmented reads	1.000 $_{10569.0}$	0.548 $_{5788.0}$	0.845 $_{8935.0}$	0.541 $_{5718.0}$	0.599 $_{6333.0}$	1.000 $_{10569.0}$
truncated reads	1.000 $_{0.0}$	0.353 $_{5777.0}$	0.000 $_{8935.0}$	0.360 $_{5718.0}$	0.293 $_{6315.0}$	0.898 $_{910.0}$
min read length	0.975 $_{5.0}$	0.721 $_{56.0}$	0.000 $_{201.0}$	0.607 $_{79.0}$	0.592 $_{82.0}$	0.950 $_{10.0}$
n50 read length	0.991 $_{1957.0}$	0.632 $_{1248.0}$	1.000 $_{1974.0}$	0.880 $_{1737.0}$	0.630 $_{1243.0}$	0.978 $_{1930.0}$
max read length	1.000 $_{11296.0}$	0.525 $_{5935.0}$	0.995 $_{11246.0}$	0.802 $_{9062.0}$	0.524 $_{5927.0}$	1.000 $_{11301.0}$
flye total length	0.932 $_{35529.0}$	0.312 $_{11879.0}$	0.952 $_{36284.0}$	0.032 $_{1232.0}$	0.256 $_{9774.0}$	1.000 $_{38108.0}$
flye n50	0.999 $_{8919.0}$	0.903 $_{8062.0}$	0.997 $_{8905.0}$	0.138 $_{1232.0}$	0.810 $_{7233.0}$	1.000 $_{8929.0}$
flye mean coverage	0.006 $_{23.2}$	0.008 $_{33.5}$	0.005 $_{21.9}$	1.000 $_{4171.0}$	0.011 $_{44.5}$	0.008 $_{35.2}$
svim structural variants	1.000 $_{33.0}$	0.000 $_{0.0}$	0.848 $_{28.0}$	0.758 $_{25.0}$	0.242 $_{8.0}$	1.000 $_{33.0}$
aggregated metric score	9.08	5.87	7.50	7.00	5.91	9.95

Table S4. Metrics for IVT RNA002 dataset.

Tool	Dorado	Tombo	f5c R.	f5c E.	Uncalled4	Dynamont
median delta ($\Delta\mu$)	0.247 $_{18.5}$	0.773 $_{58.0}$	0.873 $_{65.5}$	0.947 $_{71.0}$	1.000 $_{75.0}$	0.953 $_{71.5}$
mad delta ($\Delta\sigma$)	0.875 $_{7.0}$	0.938 $_{7.5}$	0.688 $_{5.5}$	0.750 $_{6.0}$	0.750 $_{6.0}$	1.000 $_{8.0}$
homogeneity	0.000 $_{17.0}$	0.118 $_{15.0}$	0.206 $_{13.5}$	0.176 $_{14.0}$	0.176 $_{14.0}$	0.118 $_{15.0}$
segmented reads	1.000 $_{10704.0}$	0.815 $_{8729.0}$	0.757 $_{8103.0}$	0.735 $_{7866.0}$	0.891 $_{9538.0}$	1.000 $_{10704.0}$
truncated reads	1.000 $_{0.0}$	0.183 $_{7106.0}$	0.068 $_{8103.0}$	0.099 $_{7832.0}$	0.000 $_{8696.0}$	0.944 $_{486.0}$
min read length	0.975 $_{5.0}$	0.720 $_{56.0}$	0.000 $_{200.0}$	0.700 $_{60.0}$	0.800 $_{40.0}$	0.950 $_{10.0}$
n50 read length	0.966 $_{989.0}$	0.991 $_{1015.0}$	1.000 $_{1024.0}$	0.961 $_{984.0}$	0.965 $_{988.0}$	0.953 $_{976.0}$
max read length	0.999 $_{4566.0}$	0.283 $_{1293.0}$	0.757 $_{3461.0}$	0.697 $_{3188.0}$	0.718 $_{3280.0}$	1.000 $_{4571.0}$
flye total length	1.000 $_{1421.0}$	0.000 $_{0.0}$	0.930 $_{1321.0}$	0.000 $_{0.0}$	0.000 $_{0.0}$	0.000 $_{0.0}$
flye n50	1.000 $_{1421.0}$	0.000 $_{0.0}$	0.930 $_{1321.0}$	0.000 $_{0.0}$	0.000 $_{0.0}$	0.000 $_{0.0}$
flye mean coverage	1.000 $_{4639.0}$	0.000 $_{0.0}$	0.916 $_{4249.0}$	0.000 $_{0.0}$	0.000 $_{0.0}$	0.000 $_{0.0}$
svim structural variants	1.000 $_{13.0}$	0.000 $_{0.0}$	1.000 $_{13.0}$	0.462 $_{6.0}$	0.769 $_{10.0}$	1.000 $_{13.0}$
aggregated metric score	10.06	4.82	8.12	5.53	6.07	7.92

Table S5. Metrics for *S. cerevisiae* RNA004 dataset.

Tool	Dorado	f5c R.	f5c E.	Uncalled4	Dynamont
median delta ($\Delta\mu$)	0.250 $_{13.5}$	0.935 $_{50.5}$	0.944 $_{51.0}$	0.935 $_{50.5}$	1.000 $_{54.0}$
mad delta ($\Delta\sigma$)	0.688 $_{5.5}$	0.562 $_{4.5}$	0.625 $_{5.0}$	0.625 $_{5.0}$	1.000 $_{8.0}$
homogeneity	0.000 $_{14.0}$	0.214 $_{11.0}$	0.179 $_{11.5}$	0.179 $_{11.5}$	0.000 $_{14.0}$
segmented reads	1.000 $_{10289.0}$	0.309 $_{3178.0}$	0.274 $_{2815.0}$	0.784 $_{8067.0}$	1.000 $_{10288.0}$
truncated reads	1.000 $_{0.0}$	0.606 $_{3178.0}$	0.651 $_{2813.0}$	0.000 $_{8064.0}$	0.696 $_{2448.0}$
min read length	0.975 $_{5.0}$	0.000 $_{200.0}$	0.700 $_{60.0}$	0.670 $_{66.0}$	0.985 $_{3.0}$
n50 read length	0.892 $_{1381.0}$	1.000 $_{1548.0}$	0.262 $_{405.0}$	0.077 $_{119.0}$	0.846 $_{1310.0}$
max read length	1.000 $_{114662.0}$	0.050 $_{5768.0}$	0.039 $_{4470.0}$	0.040 $_{4624.0}$	0.599 $_{68641.0}$
flye total length	0.827 $_{4177.0}$	1.000 $_{5050.0}$	0.000 $_{0.0}$	0.000 $_{0.0}$	0.870 $_{4393.0}$
flye n50	0.827 $_{4177.0}$	1.000 $_{5050.0}$	0.000 $_{0.0}$	0.000 $_{0.0}$	0.870 $_{4393.0}$
flye mean coverage	0.973 $_{852.0}$	0.825 $_{723.0}$	0.000 $_{0.0}$	0.000 $_{0.0}$	1.000 $_{876.0}$
svim structural variants	1.000 $_{15.0}$	0.133 $_{2.0}$	0.267 $_{4.0}$	0.267 $_{4.0}$	0.600 $_{9.0}$
aggregated metric score	9.43	6.64	3.94	3.58	9.47

Table S6. Metrics for CEVd RNA004 dataset.

Tool	Dorado	f5c R.	f5c E.	Uncalled4	Dynamont
median delta ($\Delta\mu$)	0.257 $_{13.5}$	0.924 $_{48.5}$	1.000 $_{52.5}$	0.981 $_{51.5}$	0.981 $_{51.5}$
mad delta ($\Delta\sigma$)	0.846 $_{5.5}$	0.692 $_{4.5}$	0.769 $_{5.0}$	0.846 $_{5.5}$	1.000 $_{6.5}$
homogeneity	0.000 $_{14.0}$	0.214 $_{11.0}$	0.143 $_{12.0}$	0.107 $_{12.5}$	0.071 $_{13.0}$
segmented reads	1.000 $_{10058.0}$	0.162 $_{1634.0}$	0.151 $_{1519.0}$	0.682 $_{6864.0}$	1.000 $_{10058.0}$
truncated reads	1.000 $_{0.0}$	0.761 $_{1634.0}$	0.778 $_{1517.0}$	0.000 $_{6832.0}$	0.302 $_{4768.0}$
min read length	0.965 $_{7.0}$	0.000 $_{200.0}$	0.785 $_{43.0}$	0.865 $_{27.0}$	0.985 $_{3.0}$
n50 read length	0.619 $_{190.0}$	1.000 $_{307.0}$	0.769 $_{236.0}$	0.531 $_{163.0}$	0.609 $_{187.0}$
max read length	1.000 $_{13344.0}$	0.147 $_{1963.0}$	0.046 $_{620.0}$	0.040 $_{537.0}$	1.000 $_{13345.0}$
flye total length	0.000 $_{0.0}$	0.000 $_{0.0}$	0.000 $_{0.0}$	0.000 $_{0.0}$	0.000 $_{0.0}$
flye n50	0.000 $_{0.0}$	0.000 $_{0.0}$	0.000 $_{0.0}$	0.000 $_{0.0}$	0.000 $_{0.0}$
flye mean coverage	0.000 $_{0.0}$	0.000 $_{0.0}$	0.000 $_{0.0}$	0.000 $_{0.0}$	0.000 $_{0.0}$
svim structural variants	1.000 $_{3.0}$	0.000 $_{0.0}$	0.000 $_{0.0}$	0.333 $_{1.0}$	1.000 $_{3.0}$
aggregated metric score	6.69	3.90	4.44	4.39	6.95

Table S7. Metrics for IVT RNA004 dataset.

Tool	Dorado	f5c R.	f5c E.	Uncalled4	Dynamont
median delta ($\Delta\mu$)	0.231 $_{12.0}$	0.798 $_{41.5}$	1.000 $_{52.0}$	0.981 $_{51.0}$	0.827 $_{43.0}$
mad delta ($\Delta\sigma$)	0.833 $_{5.0}$	0.750 $_{4.5}$	0.833 $_{5.0}$	0.917 $_{5.5}$	1.000 $_{6.0}$
homogeneity	0.000 $_{13.0}$	0.154 $_{11.0}$	0.077 $_{12.0}$	0.038 $_{12.5}$	0.000 $_{13.0}$
segmented reads	1.000 $_{10136.0}$	0.280 $_{2838.0}$	0.269 $_{2730.0}$	0.669 $_{6777.0}$	1.000 $_{10136.0}$
truncated reads	1.000 $_{0.0}$	0.580 $_{2838.0}$	0.596 $_{2729.0}$	0.000 $_{6750.0}$	0.365 $_{4284.0}$
min read length	0.970 $_{6.0}$	0.000 $_{200.0}$	0.775 $_{45.0}$	0.845 $_{31.0}$	0.990 $_{2.0}$
n50 read length	0.773 $_{256.0}$	1.000 $_{331.0}$	0.779 $_{258.0}$	0.625 $_{207.0}$	0.779 $_{258.0}$
max read length	1.000 $_{27405.0}$	0.995 $_{27265.0}$	0.031 $_{843.0}$	0.022 $_{611.0}$	1.000 $_{27406.0}$
flye total length	0.000 $_{0.0}$	0.000 $_{0.0}$	0.000 $_{0.0}$	0.000 $_{0.0}$	1.000 $_{763.0}$
flye n50	0.000 $_{0.0}$	0.000 $_{0.0}$	0.000 $_{0.0}$	0.000 $_{0.0}$	1.000 $_{621.0}$
flye mean coverage	0.000 $_{0.0}$	0.000 $_{0.0}$	0.000 $_{0.0}$	0.000 $_{0.0}$	1.000 $_{132.5}$
svim structural variants	1.000 $_{4.0}$	1.000 $_{4.0}$	0.000 $_{0.0}$	0.250 $_{1.0}$	1.000 $_{4.0}$
aggregated metric score	6.81	5.56	4.36	4.35	9.96

Table S8. Metrics for *H. sapiens* DNA dataset.

Tool	Dorado	f5c R.	f5c E.	Uncalled4	Dynamont
median delta ($\Delta\mu$)	0.269 $_{27.0}$	0.811 $_{81.5}$	0.796 $_{80.0}$	0.821 $_{82.5}$	1.000 $_{100.5}$
mad delta ($\Delta\sigma$)	0.812 $_{6.5}$	0.750 $_{6.0}$	0.750 $_{6.0}$	0.688 $_{5.5}$	1.000 $_{8.0}$
homogeneity	0.000 $_{12.0}$	0.333 $_{8.0}$	0.250 $_{9.0}$	0.333 $_{8.0}$	0.208 $_{9.5}$
segmented reads	1.000 $_{10067.0}$	0.955 $_{9617.0}$	0.941 $_{9477.0}$	0.982 $_{9887.0}$	0.993 $_{9996.0}$
truncated reads	1.000 $_{0.0}$	0.038 $_{9617.0}$	0.056 $_{9436.0}$	0.125 $_{8750.0}$	0.000 $_{9996.0}$
min read length	0.493 $_{102.0}$	0.000 $_{201.0}$	0.507 $_{99.0}$	0.493 $_{102.0}$	0.826 $_{35.0}$
n50 read length	1.000 $_{22168.0}$	0.987 $_{21876.0}$	0.917 $_{20320.0}$	0.949 $_{21039.0}$	0.943 $_{20907.0}$
max read length	1.000 $_{253314.0}$	0.375 $_{95050.0}$	0.366 $_{92838.0}$	0.242 $_{61347.0}$	0.369 $_{93590.0}$
flye total length	0.643 $_{165948.0}$	0.762 $_{196434.0}$	1.000 $_{257938.0}$	0.539 $_{138997.0}$	0.624 $_{161069.0}$
flye n50	0.318 $_{8147.0}$	0.476 $_{12177.0}$	1.000 $_{25582.0}$	0.613 $_{15687.0}$	0.403 $_{10302.0}$
flye mean coverage	0.566 $_{61.0}$	0.428 $_{46.2}$	1.000 $_{107.9}$	0.694 $_{74.9}$	0.409 $_{44.2}$
svim structural variants	1.000 $_{1112.0}$	0.000 $_{0.0}$	0.000 $_{0.0}$	0.000 $_{0.0}$	0.000 $_{0.0}$
aggregated metric score	8.10	5.92	7.58	6.48	6.78

Table S9. Metrics for Zymo HMW DNA dataset.

Tool	Dorado	f5c R.	f5c E.	Uncalled4	Dynamont
median delta (Δμ)	0.303 $_{23.0}$	0.987 $_{75.0}$	0.987 $_{75.0}$	1.000 $_{76.0}$	0.197 $_{15.0}$
mad delta (Δσ)	1.000 $_{5.5}$	0.909 $_{5.0}$	0.818 $_{4.5}$	0.818 $_{4.5}$	0.818 $_{4.5}$
homogeneity	0.562 $_{10.5}$	0.708 $_{7.0}$	0.708 $_{7.0}$	0.708 $_{7.0}$	0.000 $_{24.0}$
segmented reads	1.000 $_{10095.0}$	0.970 $_{9789.0}$	0.961 $_{9702.0}$	0.989 $_{9989.0}$	1.000 $_{10092.0}$
truncated reads	1.000 $_{0.0}$	0.030 $_{9789.0}$	0.039 $_{9699.0}$	0.116 $_{8917.0}$	0.000 $_{10092.0}$
min read length	0.951 $_{32.0}$	0.000 $_{647.0}$	0.304 $_{450.0}$	0.626 $_{242.0}$	0.998 $_{1.0}$
n50 read length	1.000 $_{13092.0}$	0.979 $_{12816.0}$	0.945 $_{12372.0}$	0.954 $_{12494.0}$	0.992 $_{12984.0}$
max read length	1.000 $_{104946.0}$	0.999 $_{104814.0}$	0.966 $_{101384.0}$	0.802 $_{84175.0}$	1.000 $_{104938.0}$
flye total length	0.985 $_{9829547.0}$	0.862 $_{8606597.0}$	0.899 $_{8970220.0}$	1.000 $_{9978966.0}$	0.926 $_{9239751.0}$
flye n50	1.000 $_{77313.0}$	0.991 $_{76623.0}$	0.740 $_{57190.0}$	0.939 $_{72620.0}$	0.991 $_{76618.0}$
flye mean coverage	0.936 $_{4.3}$	0.985 $_{4.6}$	0.906 $_{4.2}$	0.927 $_{4.3}$	1.000 $_{4.6}$
svim structural variants	1.000 $_{552.0}$	0.000 $_{0.0}$	0.000 $_{0.0}$	0.000 $_{0.0}$	0.000 $_{0.0}$
aggregated metric score	10.74	8.42	8.27	8.88	7.92

Table S10. Metrics for *S. aureus* DNA dataset.

Tool	Dorado	f5c R.	f5c E.	Uncalled4	Dynamont
median delta (Δμ)	0.263 $_{25.0}$	0.858 $_{81.5}$	0.858 $_{81.5}$	0.863 $_{82.0}$	1.000 $_{95.0}$
mad delta (Δσ)	0.923 $_{6.0}$	0.769 $_{5.0}$	0.769 $_{5.0}$	0.769 $_{5.0}$	1.000 $_{6.5}$
homogeneity	0.000 $_{11.0}$	0.364 $_{7.0}$	0.364 $_{7.0}$	0.364 $_{7.0}$	0.273 $_{8.0}$
segmented reads	1.000 $_{10044.0}$	0.985 $_{9892.0}$	0.951 $_{9556.0}$	0.966 $_{9699.0}$	1.000 $_{10044.0}$
truncated reads	1.000 $_{0.0}$	0.015 $_{9892.0}$	0.049 $_{9553.0}$	0.036 $_{9687.0}$	0.000 $_{10044.0}$
min read length	0.563 $_{90.0}$	0.000 $_{206.0}$	0.684 $_{65.0}$	0.665 $_{69.0}$	0.927 $_{15.0}$
n50 read length	0.998 $_{7717.0}$	0.990 $_{7659.0}$	0.737 $_{5704.0}$	0.912 $_{7057.0}$	1.000 $_{7735.0}$
max read length	1.000 $_{109657.0}$	1.000 $_{109644.0}$	0.565 $_{61954.0}$	0.861 $_{94377.0}$	1.000 $_{109649.0}$
flye total length	1.000 $_{3106880.0}$	0.971 $_{3017648.0}$	0.811 $_{2519367.0}$	0.847 $_{2630217.0}$	1.000 $_{3106909.0}$
flye n50	1.000 $_{2257641.0}$	0.961 $_{2170229.0}$	0.026 $_{59661.0}$	0.108 $_{242939.0}$	1.000 $_{2257665.0}$
flye mean coverage	0.787 $_{12.8}$	1.000 $_{16.2}$	0.735 $_{11.9}$	0.770 $_{12.5}$	0.787 $_{12.8}$
svim structural variants	1.000 $_{439.0}$	0.000 $_{0.0}$	0.000 $_{0.0}$	0.000 $_{0.0}$	0.000 $_{0.0}$
aggregated metric score	9.53	7.91	6.55	7.16	8.99

Table S11. Metrics for *P. anserina* DNA dataset.

Tool	Dorado	f5c R.	f5c E.	Uncalled4	Dynamont
median delta (Δμ)	0.280 $_{24.5}$	0.851 $_{74.5}$	0.857 $_{75.0}$	0.869 $_{76.0}$	1.000 $_{87.5}$
mad delta (Δσ)	0.917 $_{5.5}$	0.833 $_{5.0}$	0.833 $_{5.0}$	0.833 $_{5.0}$	1.000 $_{6.0}$
homogeneity	0.000 $_{10.5}$	0.333 $_{7.0}$	0.333 $_{7.0}$	0.333 $_{7.0}$	0.286 $_{7.5}$
segmented reads	1.000 $_{10045.0}$	0.965 $_{9693.0}$	0.965 $_{9693.0}$	0.998 $_{10028.0}$	1.000 $_{10045.0}$
truncated reads	1.000 $_{0.0}$	0.035 $_{9693.0}$	0.036 $_{9683.0}$	0.003 $_{10011.0}$	0.000 $_{10045.0}$
min read length	0.463 $_{124.0}$	0.000 $_{231.0}$	0.632 $_{85.0}$	0.597 $_{93.0}$	0.922 $_{18.0}$
n50 read length	1.000 $_{5560.0}$	0.985 $_{5474.0}$	0.924 $_{5137.0}$	0.930 $_{5171.0}$	0.999 $_{5553.0}$
max read length	1.000 $_{22177.0}$	0.970 $_{21520.0}$	0.931 $_{20636.0}$	0.912 $_{20228.0}$	1.000 $_{22169.0}$
flye total length	0.967 $_{2150191.0}$	0.830 $_{1845803.0}$	0.777 $_{1727726.0}$	1.000 $_{2222797.0}$	0.943 $_{2095390.0}$
flye n50	0.995 $_{11150.0}$	0.995 $_{11145.0}$	0.931 $_{10428.0}$	1.000 $_{11204.0}$	0.977 $_{10951.0}$
flye mean coverage	1.000 $_{19.5}$	0.639 $_{12.5}$	0.302 $_{5.9}$	0.308 $_{6.0}$	0.614 $_{12.0}$
svim structural variants	1.000 $_{1697.0}$	0.000 $_{0.0}$	0.000 $_{0.0}$	0.000 $_{0.0}$	0.000 $_{0.0}$
aggregated metric score	9.62	7.44	7.52	7.78	8.74
