# Supplementary material for: Dynamont: A comprehensive cross-species comparison of ONT segmentation tools
Source: Gigascience. 2026 Jan 19;15:giag005. doi: 10.1093/gigascience/giag005 (PMC13014473; doi:10.1093/gigascience/giag005)
Supplement: giag005_Reviewer_1_Report_Original_Submission — Ziyuan Wang -- 9/16/2025 [file giag005_reviewer_1_report_original_submission.pdf]

Reviewer Report

Title: Dynamont: A Comprehensive Cross-Species Comparison of ONT Segmentation Tools

Version: Original Submission **Date:** 9/16/2025

Reviewer name: Ziyuan Wang

Reviewer Comments to Author:

Spangenberg et, proposed Dynamont a tool to align Nanopore basedcalled bases to corresponding signal. The authors used their own HMM-based algorithm and compared them with differen tools. I like the idea and the presentation of this manuscript and the writing is quite good and clear. However, I still have some concerns related to modification reads segmentation and some stats.

1. The authors compared several tools even including Dorado --emit-moves. This part is very attractive since a lot of paper did not mention anything about ONT move tables. However, The authors should add some table or figure to show which tool relies on ONT kmer Model. https://github.com/nanoporetech/kmer_models. This will help readers better understand the relationship between signal segmentation, k-mer models, and tool performance.
2. The authors compared the performance in 12 ONT sequencing datasets which are very impressive. However, none of the datasets contain a lot of modifications. Even for the biological datasets. Nanopore sequencing has shown its potential in developing assay (PMC8896822, PMC7704922). It would be valuable to include benchmarking results on datasets containing substantial modifications, such as RNA002 (PMCID: PMC11735843), as well as RNA004 and R10.4.1 DNA datasets available from the Oxford Nanopore Open Data project.
3. ONT now provides highly accurate modification-aware basecalling models. It would benefit readers if the authors briefly discuss why continued investigation into nanopore signal segmentation remains important despite these advances, and what potential applications such segmentation could realize.
4. In the Background "high error rate" statement is not very accurate. The authors should mention that PMC11735843 and Dorado software latest model, their basecaller accuracy in modified reads is high.
5. For clarity and relevance, I suggest that the authors focus more on RNA004 and R10.4.1 DNA datasets, and consider moving the RNA002 results to the Supplementary Material. The authors should also state explicitly that only RNA004 and R10.4.1 flow cells are currently supported by ONT going forward.
6. For the forward-backward algorithm, I can follow the overall idea. However, I found Equation (2) particularly difficult to understand, and I recommend the authors revise this part to make it clearer. In previous work, many studies used a forward-only approach. Could the authors provide a biophysical justification for their choice of using the full forward-backward method in this context?

Methods

Are the methods appropriate to the aims of the study, are they well described, and are necessary controls included? Choose an item.

Conclusions

Are the conclusions adequately supported by the data shown? Choose an item.

Reporting Standards

Does the manuscript adhere to the journal's guidelines on minimum standards of reporting? Choose an item.

Choose an item.

Statistics

Are you able to assess all statistics in the manuscript, including the appropriateness of statistical tests used? Choose an item.

Quality of Written English

Please indicate the quality of language in the manuscript: Choose an item.

Declaration of Competing Interests

Please complete a declaration of competing interests, considering the following questions:

- Have you in the past five years received reimbursements, fees, funding, or salary from an organisation that may in any way gain or lose financially from the publication of this manuscript, either now or in the future?
- Do you hold any stocks or shares in an organisation that may in any way gain or lose financially from the publication of this manuscript, either now or in the future?
- Do you hold or are you currently applying for any patents relating to the content of the manuscript?
- Have you received reimbursements, fees, funding, or salary from an organization that holds or has applied for patents relating to the content of the manuscript?
- Do you have any other financial competing interests?
- Do you have any non-financial competing interests in relation to this paper?

If you can answer no to all of the above, write 'I declare that I have no competing interests' below. If your reply is yes to any, please give details below.

I declare that I have no competing interests

I agree to the open peer review policy of the journal. I understand that my name will be included on my report to the authors and, if the manuscript is accepted for publication, my named report including any attachments I upload will be posted on the website along with the authors' responses. I agree for my report to be made available under an Open Access Creative Commons CC-BY license (http://creativecommons.org/licenses/by/4.0/). I understand that any comments which I do not wish to

be included in my named report can be included as confidential comments to the editors, which will not be published.

Choose an item.

To further support our reviewers, we have joined with Publons, where you can gain additional credit to further highlight your hard work (see: https://publons.com/journal/530/gigascience). On publication of this paper, your review will be automatically added to Publons, you can then choose whether or not to claim your Publons credit. I understand this statement.

Yes Choose an item.
