# Supplementary material for: Dynamont: A comprehensive cross-species comparison of ONT segmentation tools
Source: Gigascience. 2026 Jan 19;15:giag005. doi: 10.1093/gigascience/giag005 (PMC13014473; doi:10.1093/gigascience/giag005)
Supplement: giag005_Reviewer_2_Report_Original_Submission — Daniel Depledge -- 9/25/2025 [file giag005_reviewer_2_report_original_submission.pdf]

Reviewer Report

Title: Dynamont: A Comprehensive Cross-Species Comparison of ONT Segmentation Tools

Version: Original Submission **Date:** 9/25/2025

Reviewer name: Daniel Depledge

Reviewer Comments to Author:

The manuscript by Spangenberg et al concerns segmentation of nanopore signal data. Specifically, the authors compare a range of existing segmentation tools with a new method, Dynamont, they have developed. They demonstrate that Dynamont generally outperforms all other dedicated signal segmentation tools when analyzing nanopore RNA datasets but results for DNA datasets are more mixed with no single tool outperforming the others. These are important findings as the study of RNA modifications, homopolymers, and poly(A) tails are generally reliant on high quality segmentation of signal. Importantly, the authors do an excellent job of showcasing the advantages and limitations of each tool across a range of datasets. The methods applied are well described and reproducible and the conclusions are well supported. The quality of writing is generally very good and all statistics used appear appropriate. As such, I only have a series of minor comments that I think would further strengthen this manuscript.

Introduction

* The translocation speeds listed should be noted as being approximate (as the authors note later, they are variable
* Check notations for RNA modifications - these should be written with superscript numbers and for pseudouridine, the Greek letter Psi. It should also be noted that recent Dorado versions can reportedly detect 2'O methylation.
* Check use of phrase 'timely uneven translocation'
* Check use of phrase 'reference mapping-independent'
* A short description on the difference between basecalling and signal segmentation, along with details on how Dorado can basecall without segmentation and why existing segmentation tools need basecalled reads as input would enhance the readability of this manuscript.
* The authors refer to a sampling rate of 4-5kHz but for the older chemistry / flowcells this was 3 kHz. Thus 3-5kHz would be a better description. Similarly, explicitly stating the translocation speed for the older chemistry would be useful to include.
* Figure 1 shows only 5 nucleotides within the nanopore but the right-hand side indicates kmers of size 9. It would be less confusing if 9 nucleotides were shown within the pore.
* It's not clear why f5c and uncalled4 are not segmenting at the left end of the traces in Figure 2. Could the authors comment on this.

Methods

* 'RNA reads from vero cells of nasopharyngeal swabs'. Vero cells are from African Green Monkeys and the swabs were performed on humans. I assume this should mean that virus obtained from swabs were grown in Vero cells prior to RNA isolation?

* 'Poly-A tailed' should be written as 'poly(A) tailed'
Results
* The claim that Dorado has less precise segmentation is not clear from Figure 2.
* The authors use the median/MAD delta (among other metrics) to assess segmentation tool performance. In this framework, larger signal differences near segment borders are rewarded, while smaller differences are penalized. This makes sense when adjacent segments have clearly distinct signal profiles. However, what happens when neighbouring segments are very similar? In such cases, one supposes an algorithm with lower accuracy that fails to detect the subtle border will not place a segmentation point, and therefore avoids receiving a low delta score. Conversely, a more precise algorithm that does detect the subtle border will place a segmentation point, but because the signal differences are small, it will be penalized with a low median/MAD delta. This could unfairly reduce its score, despite having segmented correctly. At the same time, failing to split two similar segments would increase within-segment variance, lowering the homogeneity score. Thus, a pattern of good delta scores combined with low homogeneity scores might signal a systematic bias in the evaluation of segmentation accuracy. This is particularly relevant because such a pattern is reported for Dynamont. Including some discussion or analysis of why this explanation can be ruled out would strengthen the manuscript.
* The IVT data are quite interesting in that Dynamont shows a significant performance increase compared to other tools. This is somewhat counterintuitive as one would expect all tools to handle the simplified (i.e. no RNA modifications) signals well. I suspect these results are thus driven by the fact that the IVT sample primarily comprises very short reads and that the strength of Dynamont is that it outperforms other tools on short read datasets (also evidenced by the CeVD data). However, it would be useful to include an additional IVT dataset that contains a wider range of read lengths (i.e. from a human cell line). There are several such datasets available from recently published manuscripts and this could help to better demonstrate the specific strengths of Dynamont.
Conclusion
* The utility of Dynamont would be strengthened by showcasing a specific example of how Dynamont improved segmentation would increase the sensitivity and/or specificity of, for instance, an analysis of RNA modifications when compared to existing tools.

Methods

Are the methods appropriate to the aims of the study, are they well described, and are necessary controls included? Choose an item.

Conclusions

Are the conclusions adequately supported by the data shown? Choose an item.

Reporting Standards

Does the manuscript adhere to the journal's guidelines on [minimum standards of reporting?]() Choose an item.

Choose an item.

Statistics

Are you able to assess all statistics in the manuscript, including the appropriateness of statistical tests used? Choose an item.

Quality of Written English

Please indicate the quality of language in the manuscript: Choose an item.

Declaration of Competing Interests

Please complete a declaration of competing interests, considering the following questions:

- Have you in the past five years received reimbursements, fees, funding, or salary from an organisation that may in any way gain or lose financially from the publication of this manuscript, either now or in the future?
- Do you hold any stocks or shares in an organisation that may in any way gain or lose financially from the publication of this manuscript, either now or in the future?
- Do you hold or are you currently applying for any patents relating to the content of the manuscript?
- Have you received reimbursements, fees, funding, or salary from an organization that holds or has applied for patents relating to the content of the manuscript?
- Do you have any other financial competing interests?
- Do you have any non-financial competing interests in relation to this paper?

If you can answer no to all of the above, write 'I declare that I have no competing interests' below. If your reply is yes to any, please give details below.

I declare that I have no competing interests

I agree to the open peer review policy of the journal. I understand that my name will be included on my report to the authors and, if the manuscript is accepted for publication, my named report including any attachments I upload will be posted on the website along with the authors' responses. I agree for my report to be made available under an Open Access Creative Commons CC-BY license (http://creativecommons.org/licenses/by/4.0/). I understand that any comments which I do not wish to be included in my named report can be included as confidential comments to the editors, which will not be published.

Choose an item.

To further support our reviewers, we have joined with Publons, where you can gain additional credit to further highlight your hard work (see: https://publons.com/journal/530/gigascience). On publication of this paper, your review will be automatically added to Publons, you can then choose whether or not to claim your Publons credit. I understand this statement.

Yes Choose an item.
